# Benefit-risk evaluation of Fuzheng Yiliu Decoction combined with chemotherapy for treating non-small cell lung cancer using multicriteria decision analysis

**DOI:** 10.3389/fonc.2025.1627904

**Published:** 2025-09-23

**Authors:** Dongjing Ma, Yingying Yang, Shenwen He, Keqin Gao, Jinbin Wang, Di Yan, Yuanchao Zhang, Jianjun Wu

**Affiliations:** ^1^ The Collaborative Innovation Center for Prevention and Control by Chinese Medicine on Diseases Related Northwestern Environment and Nutrition, Gansu University of Chinese Medicine, Lanzhou, Gansu, China; ^2^ School of Public Health, Gansu University of Chinese Medicine, Lanzhou, Gansu, China; ^3^ School of Finance, Lanzhou University of Finance and Economics, Lanzhou, Gansu, China

**Keywords:** Fuzheng Yiliu Decoction, non-small cell lung cancer, combined therapy, multicriteria decision analysis (MCDA), Traditional Chinese Medicine, clinical decision making

## Abstract

**Introduction:**

This study uses multi-criteria decision analysis (MCDA) to evaluate the benefits and risks of combining Fuzheng Yiliu Decoction with chemotherapy in treating non-small cell lung cancer (NSCLC). The aim is to improve clinical outcomes for NSCLC patients by integrating traditional Chinese medicine with conventional chemotherapy.

**Methods:**

A comprehensive literature search was conducted in PubMed, Web of Science, China National Knowledge Infrastructure (CNKI), China Biology Medicine Disc (CBM), Wanfang Databases, and China Science and Technology Journal Database (VIP Databases) to identify relevant studies on Fuzheng Yiliu Decoction combined with chemotherapy for NSCLC. Meta-analysis using RevMan 5.3 was performed to compare the effect sizes of the two treatment regimens. A MCDA model was developed to construct a value tree based on benefit-risk indicators. The benefit value, risk value, and benefit-risk ratio for both treatments were calculated using Hiview 3.2 software, followed by sensitivity analysis to assess result robustness. Monte Carlo simulations were performed using Oracle Crystal Ball 11.1 software to optimize the evaluation outcomes.

**Results:**

The literature search identified 6 randomized controlled trials (RCTs) comparing chemotherapy alone with chemotherapy combined with Fuzheng Yiliu Decoction. The MCDA model showed that the combination therapy had significantly higher benefit values (72) compared to chemotherapy alone (29). The risk value for combination therapy (56) was slightly higher than that of chemotherapy alone (24), but the overall benefit-risk value for combination therapy (68) was notably greater than chemotherapy alone (27). Monte Carlo simulations revealed a difference in total efficacy-risk values between the two treatments of 41 (95% CI: -16.59, 38.73). The probability that the combination therapy’s benefit-risk value exceeds that of chemotherapy alone was 81.83%.

**Discussion:**

These findings suggest that combining Fuzheng Yiliu Decoction with chemotherapy improves therapeutic efficacy and reduces chemotherapy’s adverse side effects, offering a promising treatment strategy for NSCLC. This study provides valuable insights into enhancing treatment strategies and clinical decision-making in managing NSCLC.

## 1 Introduction

According to the 2022 Global Cancer Statistics report, lung cancer is the most prevalent malignant tumor globally and the leading cause of cancer-related deaths (1, 2). Non-small cell lung cancer (NSCLC) represents the most common pathological subtype, accounting for approximately 85% to 90% of cases (3, 4). Despite advancements in surgical techniques, chemotherapy, radiotherapy, targeted therapy, and immunotherapy, the 5-year survival rate for NSCLC remains below 25%. Chemotherapy is the most commonly employed treatment in clinical practice and can effectively extend patient survival; however, its toxic side effects significantly impact patients’ quality of life (5, 6). The treatment of NSCLC continues to pose a considerable challenge in the field of oncology. Notably, traditional Chinese medicine(TCM) has gained significant attention and research as an adjunctive and alternative therapy for cancer, more and more people are recognizing its potential in enhancing the effectiveness of Western medicine treatments and alleviating their side effects (7).

In TCM, LUAD is classified under the categories of ‘pulmonary retention’ and ‘lung amassment (8),’ and is caused by a deficiency in Zheng Qi and dysfunction in the body’s immune system (9). Therefore, strategies such as medicinal treatments are necessary to strengthen this vital energy.

Fuzheng Yiliu Decoction(FZYLF) is a representative formula for tonifying the body’s Zheng Qi, is composed of several herbs, including Huang Qi, Tai zi Sheng, Dang Sheng, Bai Shao, Bai She, She Cao, Ban Zhi Lian, and Hu Zhang, with modifications made based on the patient’s condition. It is recognized for its potential to reduce postoperative cancer recurrence, enhance immune function, and prolong survival(R1-P1) (10–12). Current studies have confirmed that the Fuzheng Yiliu Decoction has a positive impact on various cancers, including lung cancer, glioma, stomach cancer, prostate cancer, colorectal cancer, and liver cancer (10, 13–15). Recent studies have highlighted the clinical efficacy of combining Fuzheng Yiliu Decoction with chemotherapy (16). However, there is a notable absence of systematic investigations assessing the use of Fuzheng Yiliu Decoction in conjunction with chemotherapy specifically for the treatment of NSCLC. Although some studies have performed basic comparative analyses of efficacy and adverse event rates derived from clinical randomized controlled trials (17), these investigations don’t offer a comprehensive evaluation of both the effectiveness and safety of this treatment. Consequently, the analysis of its benefits and risks remains incomplete.

A comprehensive and quantitative evaluation of the benefits and risks associated with combination therapy is crucial for determining the most effective treatment strategy. Currently, Benefit-risk assessment methods are divided into two main categories: qualitative and quantitative. Notably, MCDA is recognized as one of the most widely accepted and applicable quantitative approaches (18) (R2-P11).

This study aims to utilize both Meta-analysis and MCDA model to quantitatively and comprehensively assess the advantages and disadvantages of combining Fuzheng Yiliu Decoction with chemotherapy for the treatment of NSCLC. This approach will facilitate a holistic understanding of the treatment’s benefits and drawbacks, thereby providing valuable insights for clinical decision-making.

## 2 Materials and methods

### 2.1 Literature search

Utilizing databases such as PubMed, Web of Science, China National Knowledge Infrastructure (CNKI), China Biology Medicine Disc (CBM), and Wanfang Databases and China Science and Technology Journal Database(VIP Databases). The focus should be on identifying clinical controlled trials that investigate the combined therapy of Fuzheng Yiliu Decoction and platinum-based chemotherapy regimens for NSCLC, with a cut-off date of March 31, 2024. Relevant keywords for this search include Fuzheng Yiliu Decoction, cisplatin, NSCLC, lung adenocarcinoma, lung squamous cell carcinoma, and randomized controlled trial(RCT). The search strategy is shown in **Table 1**.

**Table 1:** Search strategy.

Data base	Search strategy
PubMed	(FuzhengYiliutang[Title/Abstract]) (R2-P4)
Web of Science	TS=(FuzhengYiliutang) AND (NSCLC OR non-small cell lung cancer) AND (Cisplatin-resistant)
CNKI	(FuzhengYiliutang) AND (NSCLC OR non-small cell lung cancer) AND (Cisplatin-resistant)
Wan Fang	(FuzhengYiliutang) AND (NSCLC OR non-small cell lung cancer) AND (Cisplatin-resistant)
VIP	(FuzhengYiliutang) AND (NSCLC OR non-small cell lung cancer) AND (Cisplatin-resistant)

#### 2.1.1 Literature management and data extraction

Use EndNote.20 software for literature management and create data extraction tables using Excel. Two researchers independently screened, provided, and cross reviewed the data. If there is a dispute, it can be decided through discussion or consultation with a third party. When screening materials, first consult the summary of the article title, and after removing obviously irrelevant materials, then consult the entire article to determine whether it is included. The extracted content includes the author, publication time, sample size, and intervention measures of the experimental group Intervention measures, outcome indicators, etc. for the control group.

#### 2.1.2 Quality evaluation

Two researchers used the bias risk assessment tool recommended by the Cochrane Handbook for evaluation, including random sequence generation, allocation scheme concealment, blinding of study subjects and researchers, blinding of outcome assessors, completeness of outcome data, selective reporting, and other sources of bias. The included studies were judged as low-risk, high-risk, and uncertain risk.(R1-P2,R2-P4/6).

#### 2.1.3 Inclusion criteria

Research type: Randomized Controlled Trial (RCT).

Research subjects: This study involves patients diagnosed with NSCLC.

Interventions: The experimental group will receive oral traditional Chinese medicine formulations in conjunction with chemotherapy, while the control group will undergo chemotherapy alone.

Outcome Measures: The efficacy of the treatment will be evaluated using several benefit indicators, including Karnofsky Performance Status (KPS) score, CA211, carcino embryonic antigen (CEA), and Cancer Fatigue Scale scores. List gastrointestinal adverse reactions as risk indicators.

#### 2.1.4 Exclusion criteria

Animal studies, systematic reviews/meta-analyses, expert reviews, conference papers, studies combining other cancers, inability to access full text, studies with interventions or control measures not meeting criteria.

### 2.2 MCDA model

#### 2.2.1 Construct evaluation index decision-making system

**Figure 1:**
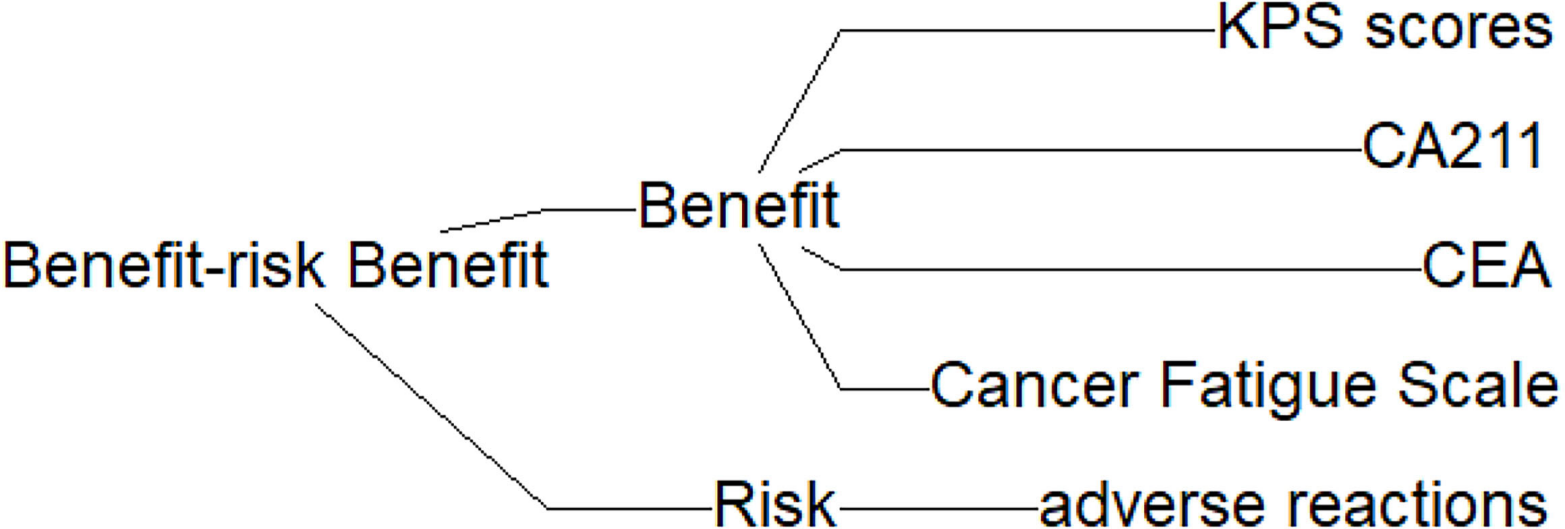
Decision tree of benefit-risk indicators for combined therapy of Fuzheng Yiliu Decoction in the treatment of NSCLC.

We propose the construction of an evaluation indicator decision tree that encompasses both efficacy and risk indicators. Efficacy indicators were defined as those that reflect the effectiveness and safety of medications in clinical controlled trials pertaining to NSCLC, as identified through literature searches. Examples of efficacy indicators include, KPS scores, CA211, CEA, and Cancer Fatigue Scale scores. Risk indicators is the incidence of gastrointestinal adverse reactions. Each indicator is represented visually in the form of an effects tree, as illustrated in **Figure 1**.

Due to the varying clinical significance of each efficacy and risk indicator, the establishment of attribute weights will directly impact the selection of decision-making schemes. This study employs a modified Delphi method (19) to construct a weighting framework: participating experts independently provide their opinions based on a standardized questionnaire designed by the research team, with unidirectional isolation maintained between experts throughout the process (communication is only with the researchers). After two rounds of expert consultation, it has been determined that 75% and 25% of the weight will be allocated to the benefit indicators and risk indicators respectively(R2-P1). To further refine the precision of weight allocation, the study introduces swing weighting method for dynamic calibration (20). The weight of the relatively important indicator is 100; other indicators are assigned values according to their importance in comparison (21). The values are assigned based on reported literature and expert opinions from clinical professionals. Additionally, we made appropriate adjustments based on the results of the meta-analysis. For indicators with high heterogeneity, we reduced their weight allocation accordingly. (R2-P2) Compares various parameters against the Cancer Fatigue Scale score, assigned the highest weight of 100%. The KPS scores is weighted at 80%, while the weights for CA211, CEA are set at 60%. The occurrence rate of gastrointestinal adverse reactions is assigned a weight of 100%. For further details, please refer to **Table 2**.

**Table 2:** Benefit-risk indicator weights, optimal values, and worst values for multicriteria decision analysis (MCDA)(R2-P15).

Classification	Weight %	Indicator	Weight %	Optimal value	Worst value
Benefit	75	KPS score	80	11	2
		CA211	60	6	3
		CEA	60	12	6
		Cancer Fatigue Scale score	100	18	0
Risk	25	gastrointestinal adverse reactions	100	0	1

#### 2.2.2 Data analysis

Integrate the benefit-risk indicator-related outcome data using the data processing software RevMan 5.3, For binary data, Incidence Rate(IR) is used, while for continuous variable data, mean difference (MD) is used (22) select the most appropriate model for all data analyses. Each effect variable provides its point estimate and 95% confidence interval (α=0.05).

#### 2.2.3 *Sores* (R2-P3/P8/P10)

The optimal and worst values for efficacy and risk indicators were systematically derived from the 95% confidence intervals (CIs) obtained through meta-analysis of clinical trial data (23). For efficacy outcomes, optimal=experimental group’s upper CI (best improvement), worst=control group’s lower CI (baseline). For risks, optimal=experimental group’s lower CI (minimal risk), worst=control group’s upper CI (maximal risk). which please refer to **Table 2**.

The single-attribute utility function (SAUF) (24) is utilized to address the issue of inconsistent data dimensions among different indicators, converting each indicator’s data into preference values ranging from 0 to 100. In this context, a higher preference value for efficacy indicators signifies greater efficacy, with a maximum value of 100 representing optimal efficacy. Conversely, a lower preference value for risk indicators indicates a higher associated risk, with a minimum value of 0 representing the worst risk (25). The optimal and worst values for each indicator are detailed in **Table 2**. The SAUF formula are as follows,


$$U_{benefit}=(U-U_{\min}/U_{\max}-U_{\min})\times 100$$

$$U_{risk}=(U_{\max}-U/U_{\max}-U_{\min})\times 100$$

*U* represents the actual value of the current indicator, *U*_min_represents the minimum observed value of this indicator across all candidate options, *U*_max_ represents the maximum observed value of this indicator across all candidate options.

#### 2.2.4 Benefit risk value calculation

Subsequently, we combine the weights and preference values of efficacy and risk indicators to calculate the respective efficacy values, risk values, and total efficacy-risk values using Hiview 3.2 for thetwo schemes(R2-P8). The calculation formula (26) is as follows, $U=\sum_{j=1}^{\text{n}}\varpi_{j}U_{ij}$ , ‘*U*ij’ represents the preference score of decision alternative ‘i’ on criterion ‘j’, while ω denotes the weight of the criterion.

#### 2.2.5 Sensitivity analysis

Given the subjective nature of assigning weights to indicators, it is essential to conduct a sensitivity analysis to verify the reasonableness of the assigned weights. Experience has demonstrated that a significant impact on evaluation results occurs when changes in relative weights exceed 20% (27). Our study applied Hiview 3.2 software to observe the impact of adjusting the relative weights of various indicators on the ranking of decision options (R2-P16).

#### 2.2.6 Monte Carlo simulation

Since the results of benefit risk assessment were point estimates in the meta-analysis, the data were uncertain (24). This study used Oracle Crystal Ball 11.1.3 software (Oracle, USA) to run Monte Carlo simulation. We perform an uncertainty analysis on the established model to mitigate data fluctuations (28). We assumed that the effect values followed a triangular distribution. A total of 10,000 individual simulations were performed to derive the efficacy, risk, and overall benefit-risk differences between the single-treatment group and the combination group, along with their respective 95% confidence intervals. Additionally, we calculated the probabilities of these differences appearing in the model results. By replacing point estimates with probability distributions, we were able to support decision-making and optimize the outcomes accordingly (23)(R2-P7/P9). This approach, utilizing MCDA and Monte Carlo simulations, allowed for a more robust evaluation of the treatment options.

## 3 Results

### 3.1 Include studies

**Figure 2:**
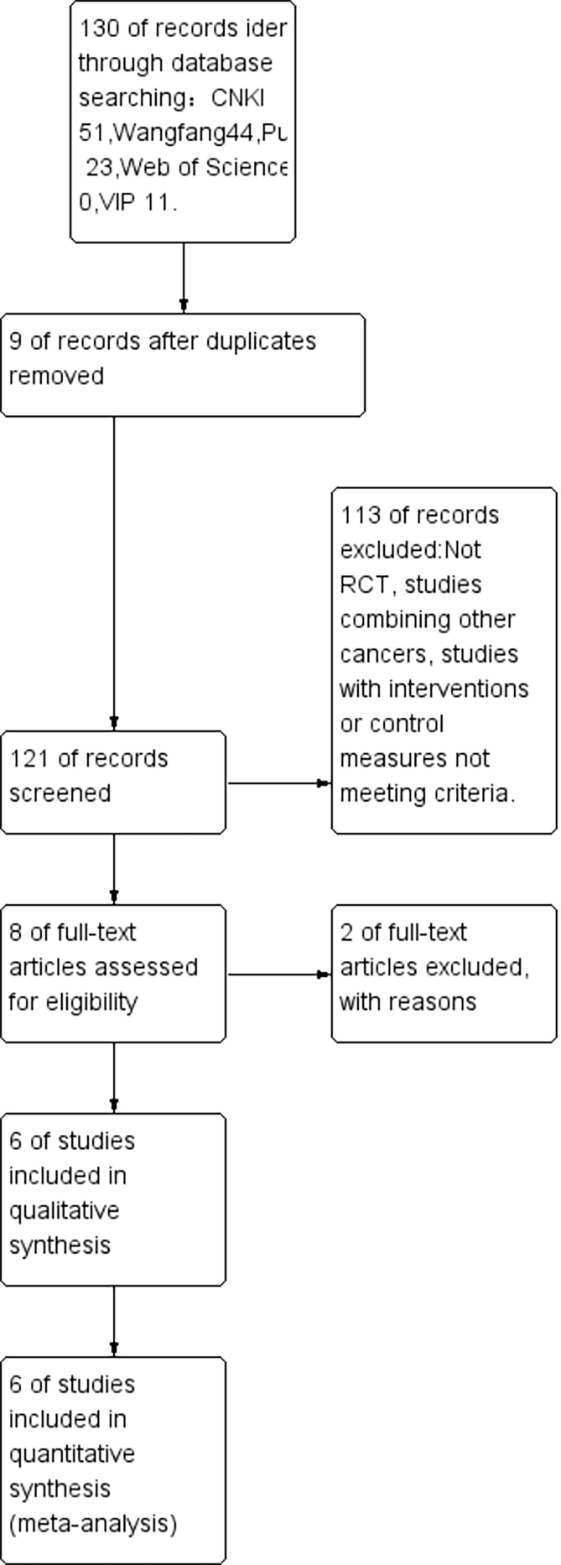
Literature screen.

**Figure 3:**
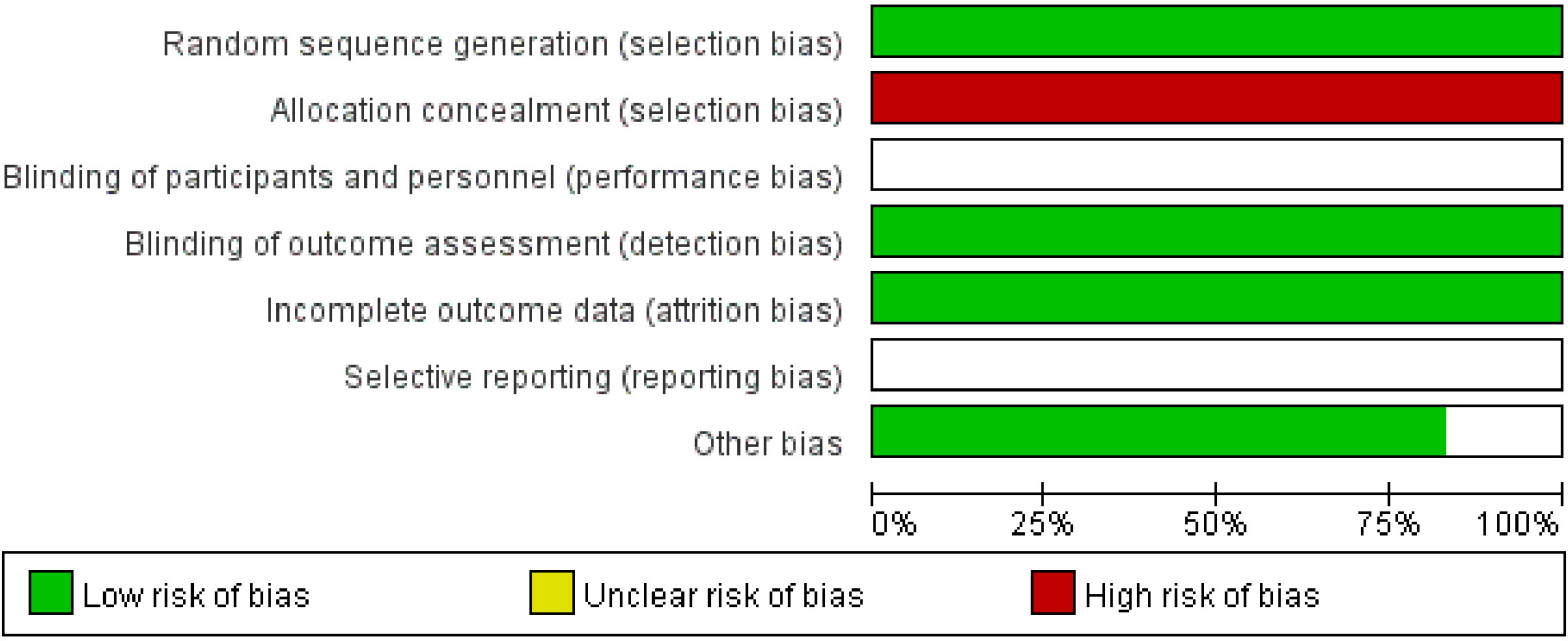
Risk of bias.

This study initially identified 130 articles through a comprehensive literature search. After applying the inclusion criteria and excluding irrelevant studies, a total of 6 RCTs were ultimately included in the analysis. The literature screening process is shown in **Figure 2**. Among the six included studies, all reported using randomization with only one specifying the random sequence generation method. Crucially, none described allocation concealment procedures, resulting in a high risk of selection bias. The implementation of blinding (of participants, personnel, or outcome assessors) was not mentioned in any study, leading to an unclear risk of performance and detection bias. All studies were unable to obtain a research plan and determine whether to selectively report the results. Regarding other potential biases, baseline characteristics were generally balanced between groups except for one study where baseline comparability was unclear due to insufficient reporting. The evaluation of literature quality is shown in **Figure 3** (R2-P4/P6). Details of the included literature are presented in **Table 3**.

**Table 3:** Basic information of included literature.

Literature	E\C	Control	Experimental	Outcome
ZhangWei (29)	45/45	chemotherapy	combined chemotherapy with Fuzheng Yiliu Decoction	④
Sun Ailin (30)	29/28	chemotherapy	combined chemotherapy with Fuzheng Yiliu Decoction	①②③⑤
Lv Pengqiang (31)	30/30	chemotherapy	combined chemotherapy with Fuzheng Yiliu Decoction	①②③⑤
Zhang Wei- wei (32)	20/20	chemotherapy	combined chemotherapy with Fuzheng Yiliu Decoction	①
Kong Xiangying (33)	30/30	chemotherapy	combined chemotherapy with Fuzheng Yiliu Decoction	④
Li Riliang (34)	24/24	chemotherapy	combined chemotherapy with Fuzheng Yiliu Decoction	①

①KPS sores ②CA211 ③ CEA ④ Cancer Fatigue Scale score ⑤ gastrointestinal adverse reactions (R2-P15).

### 3.2 Consolidation result

The efficacy of combined use of Fuzheng Yiliu and chemotherapy alone, The number of RCTs for benefit and risk indicators, the combined results of meta-analysis, and The P-value is shown in **Table 4**. The results indicate that the treatment strategy of combining chemotherapy with Fuzheng Yiliu Decoction improves patient outcomes and significantly reduces the incidence of adverse reactions compared to conventional chemotherapy in the control group for NSCLC.

**Table 4:** Merger results of Experimental and control of each indicator (R2-P8/P15).

Primary indicator	Secondary indicator	Experimental		Control	
		RCT	Combined results (95%CI,P<0.0001)	RCT	Combined results (95%CI,P<0.0001)
Benefit	KPS score	4	-9.38 (-10.91, -7.86)	4	-3.63 (-5.35, -1.92)
	CA211	2	4.98 (4.24, 5.72)	2	3.89 (3.19, 4.60)
	CEA	2	9.96 (8.68, 11.25)	2	7.58 (6.50, 8.67)
	Cancer Fatigue Scale score	2	14.58 [11.17, 17.98]	2	3.70 (0.29, 7.11)
Risk	gastrointestinal adverse reactions	2	0.44 [0.31,0.57]P=0.36	2	0.76 [0.62, 0.86]P<0.001

### 3.3 Comprehensive benefits

**Figure 4:**
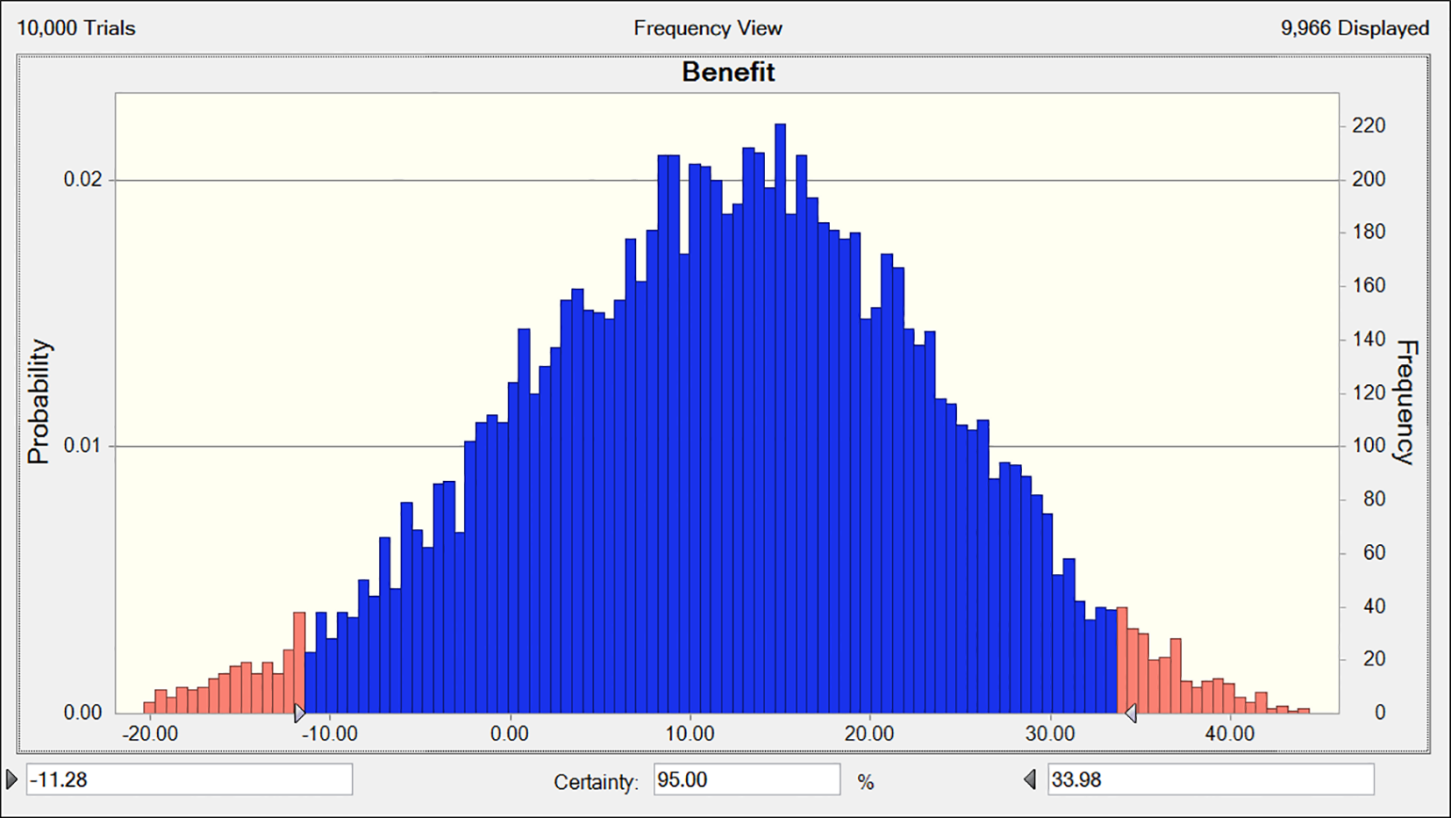
Differential benefits between experimental and control (R2-P14).

The efficacy values for combined chemotherapy with Fuzheng Yiliu Decoction and chemotherapy alone are72and 29, respectively, as illustrated in **Table 5**. These results indicate that the combination therapy of Fuzheng Yiliu Decoction and chemotherapy demonstrates superior efficacy in the treatment of NSCLC. Additionally, a Monte Carlo simulation was employed to analyze the difference in efficacy values, revealing a difference of 43 (95% CI -11.28, 33.98) as presented in **Figure 4**.

**Table 5:** Weight and weight score of each indicator for alone and combined (R2-P15).

Classification	Weight%	Indicator	Weight%	Benefit value/risk value		Relative weight %
				Experimental	Control	
Benefit	75	KPS score	80	82	18	20
		CA211	60	67	33	15
		CEA	60	75	25	15
		Cancer Fatigue Scale score	100	66	36	25
Overall benefit value				72	29	
Risk	25	gastrointestinal adverse reactions	100	56	24	25
Overall risk value				56	24	

### 3.4 Comprehensive risk

**Figure 5:**
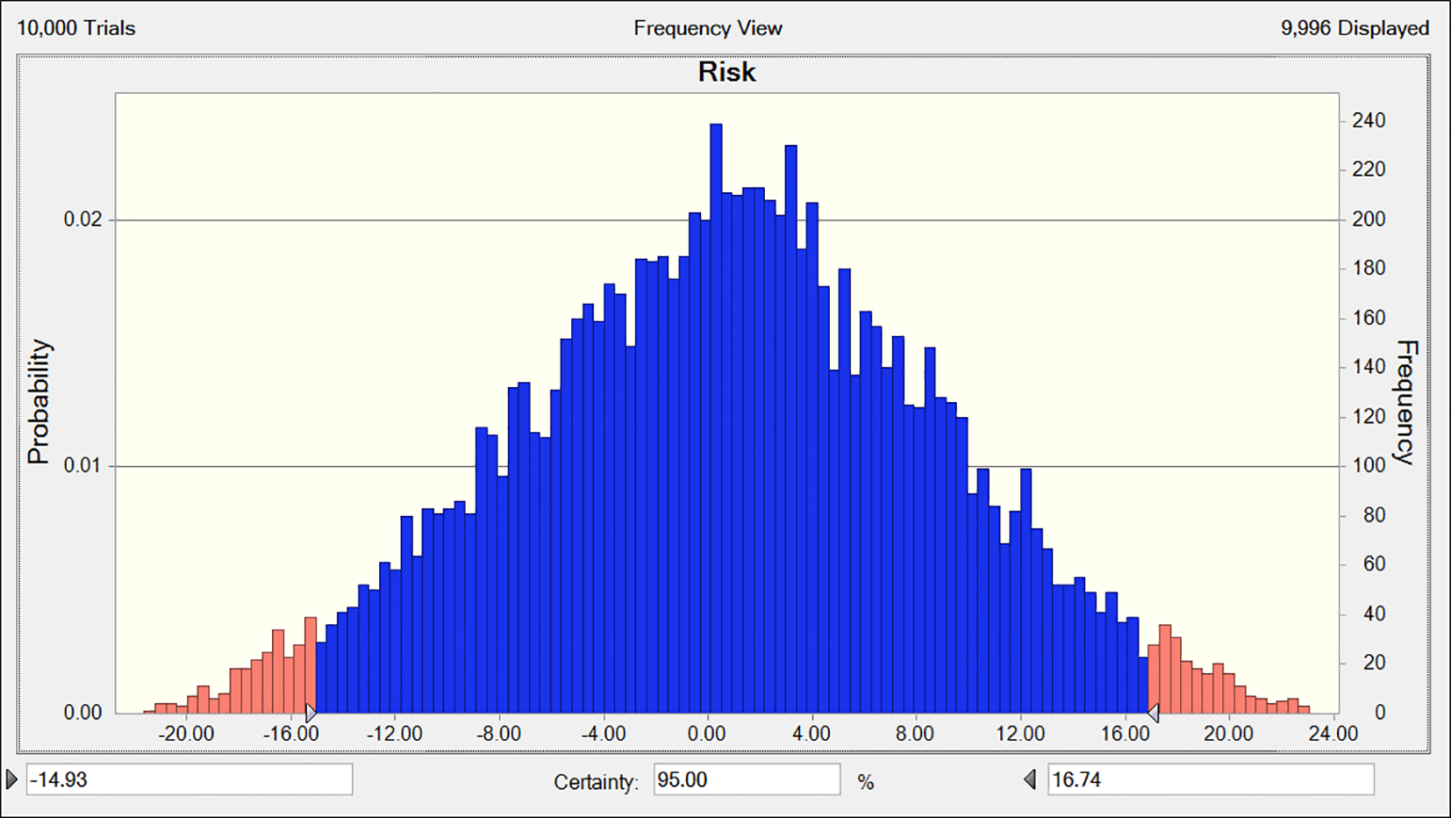
The deviation of risk between experimental and control (R2-P14).

The risk values for combined chemotherapy with Fuzheng Yiliu Decoction and chemotherapy alone are 56 and 24, respectively, as presented in **Table 5**. A Monte Carlo simulation reveals a difference in risk values of 32 (95% CI -14.93, 16.74) between the two treatment modalities, as illustrated in **Figure 5**.

### 3.2 Comprehensive benefits-risk

**Figure 6:**
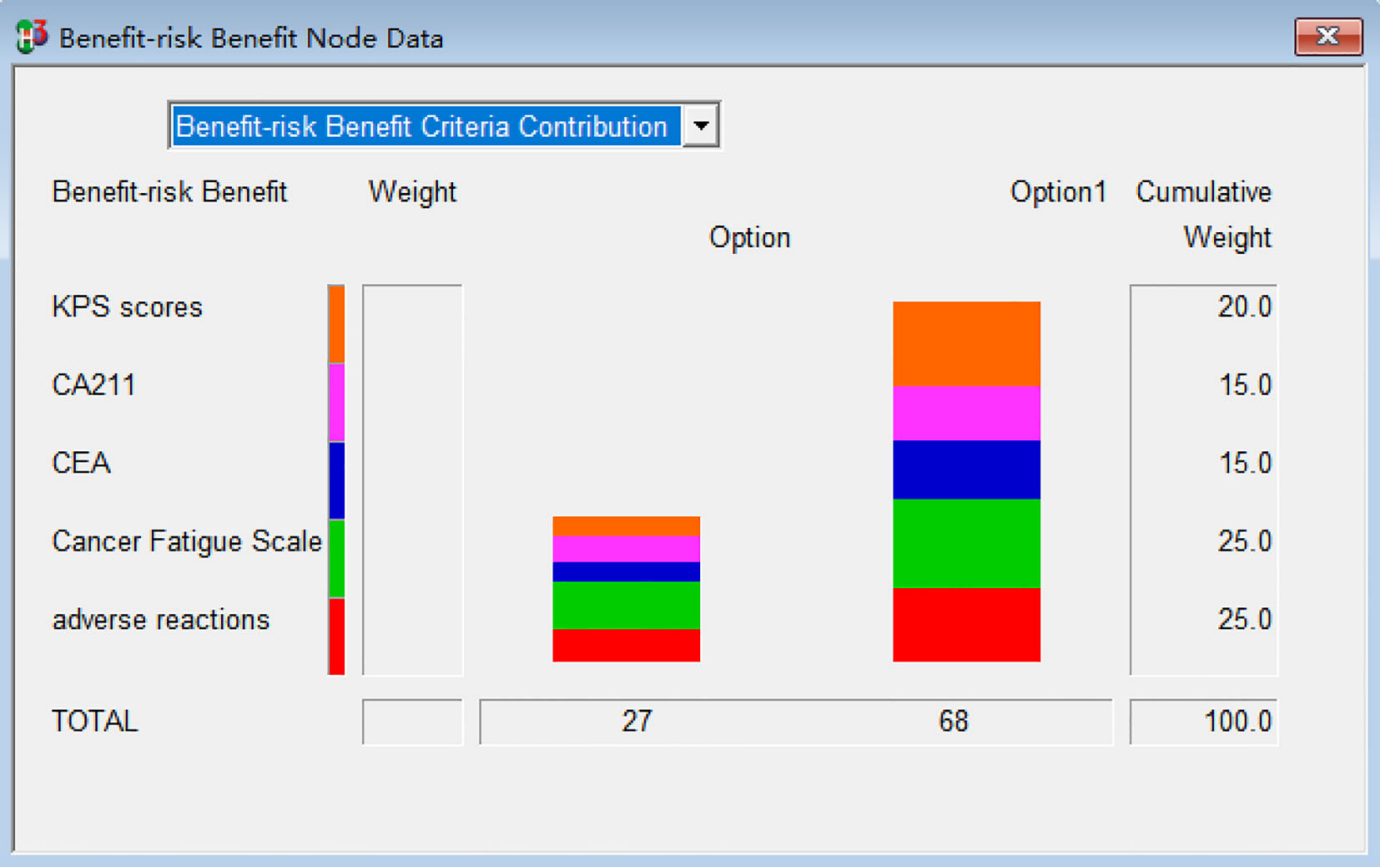
Total benefit-risk value of two regimens.

**Figure 7:**
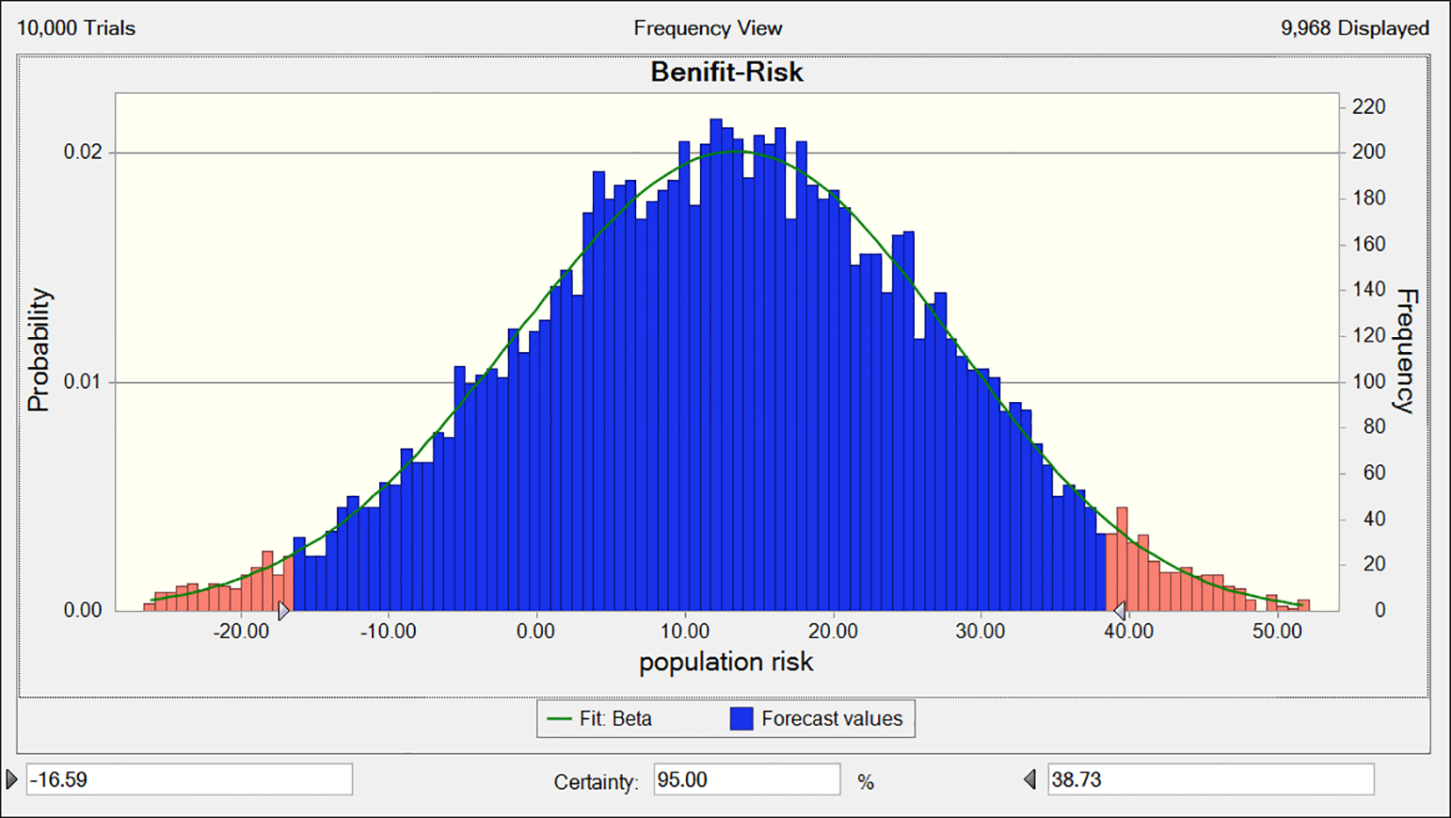
The deviation of benefit-risk between experimental and control (R2-P14).

**Figure 8:**
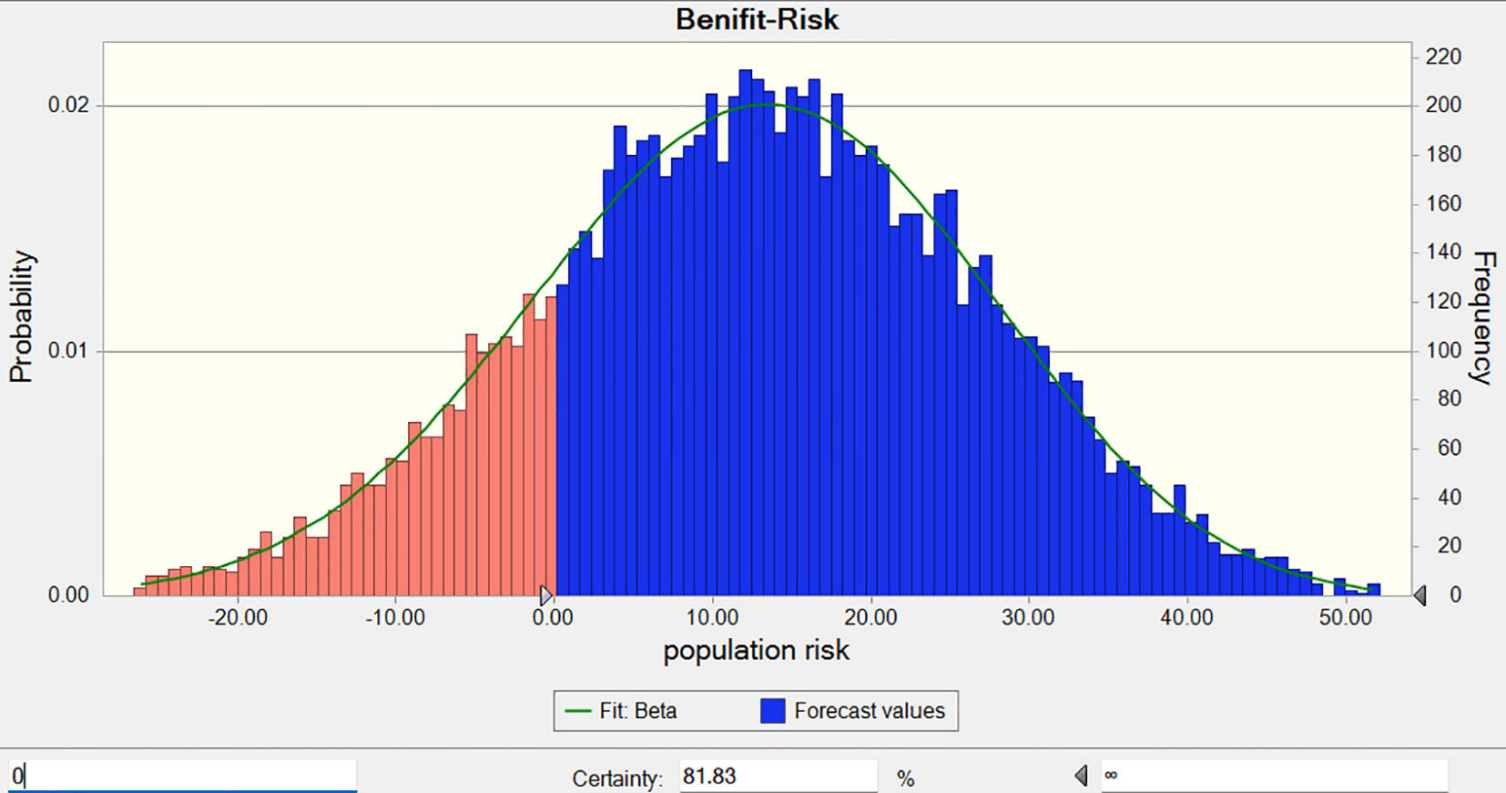
The probability of experimental being better than control (R2-P14).

The total benefits-risk values for the combined use of Fuzheng Yiliu Decoction and chemotherapy, compared to chemotherapy alone, are 68 and 27, respectively, as illustrated in **Table 5** and **Figure 6**. A Monte Carlo simulation reveals that the difference in total efficacy-risk values between the two treatment modalities is 41 (95% CI -16.59, 38.73), as depicted in **Figure 7**. Furthermore, the probability that the total benefit-risk value of the combination of Fuzheng Yiliu Decoction and chemotherapy for treating NSCLC exceeds that of chemotherapy alone is 81.83%, as shown in **Figure 8**.

### 3.3 Sensitivity analysis

**Figure 9:**
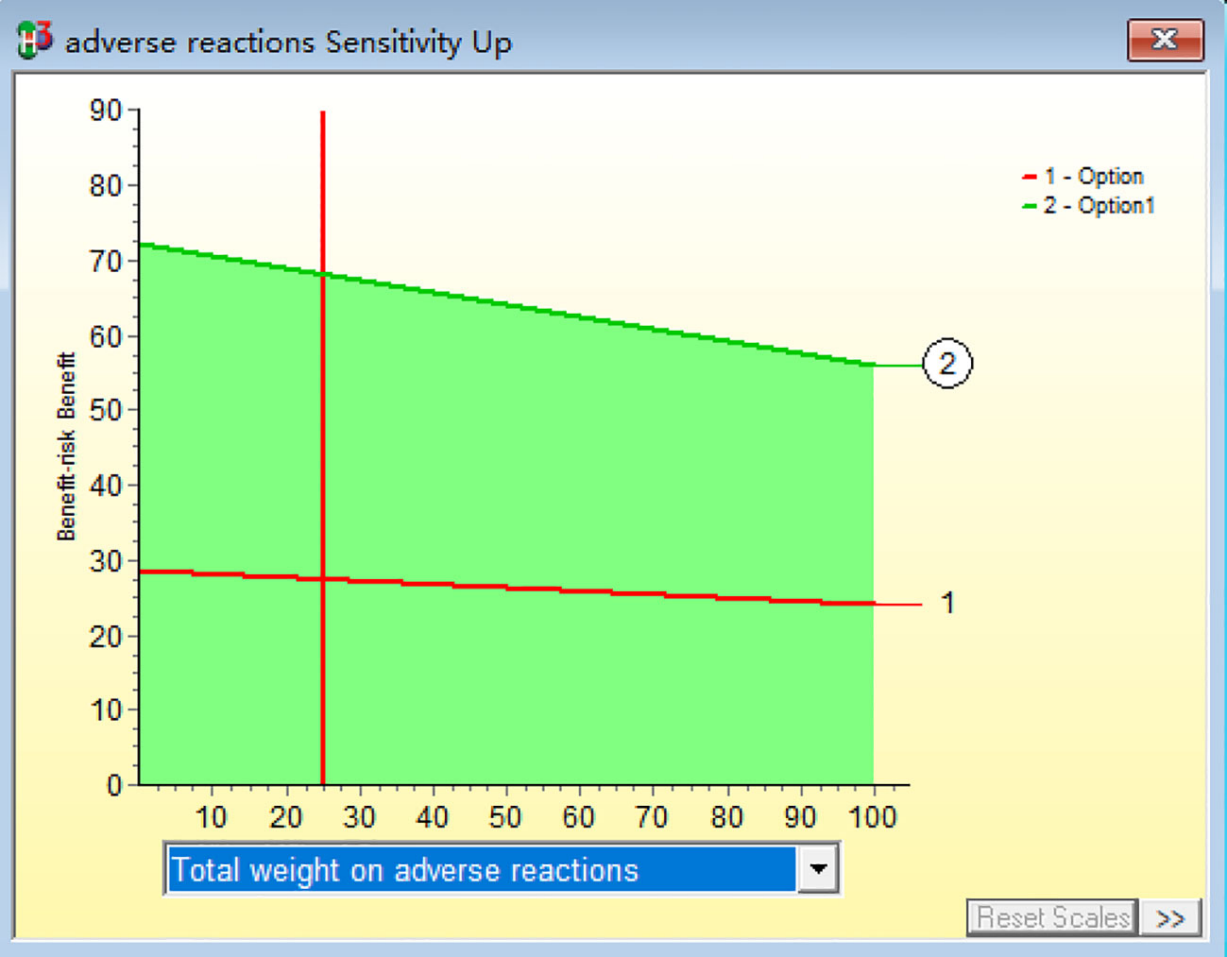
Sensitivity analysis of benefit-risk.

Within a 20% change in the weightings, the ranking of the overall risk-benefit assessment does not change, with the combined treatment group remaining ranked higher than the monotherapy group.(R2-P16)This finding indicates that the study outcomes are robust across varying weighting schemes, demonstrating the high stability of the benefit-risk evaluation model, as shown in **Figure 9**.

## 4 Discussion

### 4.1 MCDA

MCDA is a quantitative, structured decision-support framework that enables systematic value assessment through multi-dimensional evaluation criteria (35). By constructing decision matrices (incorporating alternative sets and attribute sets), assigning attribute weights, and evaluating alternative merits via standardized processes, this methodology ultimately identifies optimal decision alternatives (36). Since its conceptual development, MCDA has been widely adopted across diverse sectors including energy, environment, military, management, construction, and public governance (37). In healthcare decision-making, MCDA has emerged as a critical tool for medical policy formulation and benefit-risk assessment. While qualitative methods remain predominant in regulatory practice due to their operational simplicity, particularly under data-scarce conditions, their inherent subjectivity and lack of transparency often compromise result reproducibility and reliability (38). Global regulatory trends are increasingly shifting toward structured quantitative approaches to enhance decision transparency and patient engagement (39). MCDA demonstrates unique advantages in pharmaceutical benefit-risk evaluation through its transparency and adaptability, leading to formal adoption by multiple regulatory authorities worldwide (40).

Traditional quantitative benefit-risk methods in pharmacology, often termed “epidemiological evaluation,” include well-established metrics like Number Needed to Treat (NNT) and Number Needed to Harm (NNH). Though theoretically robust and widely accepted, these approaches primarily focus on clinical efficacy and adverse events, representing a narrow interpretation of benefit-risk assessment (38). China’s Center for Drug Evaluation (CDE) in the 2023 “Technical Guidelines for Benefit-Risk Assessment of New Drugs,” both explicitly recommend MCDA as a preferred quantitative tool for comprehensive benefit-risk evaluation.(R2-P12/19).

Notable progress has been made in integrating MCDA with traditional medicine systems. Recent studies have established methodological frameworks for MCDA application in Chinese medicine formulation decisions, including quantitative benefit-risk assessment of sinomenine preparations for rheumatoid arthritis treatment and comparative analysis of clinical outcomes among three commonly used Chinese medicine injections for hepatocellular carcinoma (23, 24). These innovations not only lay groundwork for standardized MCDA implementation in traditional medicine but also provide novel methodological support for enhancing scientific rigor and transparency in clinical decision-making within this field.(R2-P18).

### 4.2 Efficacy

Non-small cell lung cancer (NSCLC) is characterized by insidious onset and rapid progression, most of patients being diagnosed at an advanced stage. Its high recurrence rate and poor prognosis pose significant treatment challenges (41). Traditional Chinese Medicine (TCM) believes that the root cause of lung cancer lies in “deficiency of vital energy and internal accumulation of pathogenic factors. “In advanced lung cancer, patients not only face a decline in efficacy but also commonly experience progressive physical weakness, weight loss, and other signs of body function decline (30). TCM suggests that supporting the vital energy and strengthening the foundation should be incorporated into the treatment, based on the patient’s symptoms. Cancer-related fatigue occurs in 70%-100% of cancer patients, not only during antitumor treatment but also persisting for months or even years after the treatment ends, significantly affecting the quality of life (42). TCM clinical practice suggests that cancer-related fatigue in advanced NSCLC patients after chemotherapy is often related to spleen and stomach weakness and Qi and blood deficiency. By regulating the spleen and stomach functions and replenishing Qi and blood, fatigue symptoms can be effectively alleviated, and the body’s functional state can be restored, forming a beneficial therapeutic cycle of “supporting the vital energy to expel pathogenic factors” (43) (R2-P11). In this study, the combination therapy group demonstrated higher benefit scores compared to chemotherapy alone in both the KPS score and Cancer Fatigue Scale score. This comprehensive efficacy evaluation result indicates that the combination of Fuzheng Yiliu Decoction and chemotherapy significantly improves the Karnofsky Performance Status (KPS) score, alleviates cancer-related fatigue symptoms, enhances chemotherapy tolerance and completion rates, and ultimately achieves the goal of increasing efficacy while reducing toxicity.

Furthermore, it is currently believed that the combined use of tumor markers such as CEA, NSE, Cyfra 21-1, ProGRP, and SCC can improve the sensitivity and specificity of lung cancer diagnosis. Among them, the diagnosis of NSCLC mainly relies on elevated levels of CEA and Cyfra 21-1 (31) (R2-P11). The combination of Fuzheng Liu Fang and chemotherapy significantly reduces serum tumor markers such as CEA and CA211, and randomized controlled trials have consistently confirmed these findings.

The Monte Carlo simulation results reaffirmed our findings. The results show that the combination of Fuzheng Yiliao Decoction and chemotherapy is superior to chemotherapy alone, with more significant treatment effects. Notably, there are more significant improvements in therapeutic efficacy evaluation, cancer fatigue scale scores, and KPS scores, suggesting that the combination of Fuzheng Yiliao Decoction and chemotherapy can significantly improve the quality of life and prognosis of elderly patients with advanced NSCLC.

### 4.3 Risk

Chemotherapy, as the core treatment for advanced non-small cell lung cancer (NSCLC), primarily involves platinum-based doublet regimens (such as gemcitabine, vinorelbine, docetaxel, or paclitaxel combined with platinum drugs), which inhibit tumor cell proliferation through cytotoxic effects (44). However, while chemotherapy kills tumor cells, it also induces a pathological state of “further depletion of Zheng Qi”. After chemotherapy, the pathological state of “further depletion of Zheng qi” becomes further aggravated, usually accompanied by a series of toxic side effects, including nausea, vomiting, and appetite loss, which significantly affect the patient’s quality of life (32).

Given the risk of chemotherapy, clinical decision-making needs to strengthen the risk assessment system (R2-P11). This study evaluates the risks associated with two treatment strategies by utilizing gastrointestinal adverse reactions as risk indicator. The included randomized controlled trials (RCTs) indicates that compared to the combined approach of Fuzheng Yiliu Decoction with chemotherapy, chemotherapy alone has a greater propensity to induce significant gastrointestinal adverse reactions (such as nausea, vomiting, and anorexia). The comprehensive risk assessment results approve that chemotherapy alone demonstrated a lower score. In contrast, higher-scoring combined treatment approaches (such as Fuzheng Yiliu Decoction combined with chemotherapy) may better alleviate chemotherapy-induced side effects, enhance patient tolerability, and improve the overall safety profile.

### 4.4 Benefit risk

In clinical decision-making, it is crucial to comprehensively assess the benefits and risks of various treatment options (R2-P11). This study integrates meta-analysis and multi-criteria decision analysis to quantify the benefits and risks of the combined use of Fuzheng Yiliao Decoction and chemotherapy in the treatment of non-small cell lung cancer (NSCLC). During the model construction, weights were determined through two rounds of Delphi expert consultation, followed by refinement using the swing weighting method, and cross-validated using both expert experience and meta-analysis results. When statistical heterogeneity was high, the weight of certain indicators was appropriately reduced to avoid over-reliance on a single type of evidence. The final weight distribution was 75% for benefit indicators and 25% for risk indicators. The decision tree model was constructed using Hivew3.2 software, and the risk-benefit preference values were calculated. Monte Carlo simulations were used to output the probability differences between the two treatment decisions, thereby supporting and optimizing the decision-making process.

The final results of the model indicated that the benefit-risk ratio of combining Fuzheng Yiliao Decoction with chemotherapy was superior to chemotherapy alone in treating NSCLC. This finding suggests that the combination of Fuzheng Yiliao Decoction with chemotherapy can enhance the therapeutic effect of Western medicine, reduce the side effects of chemotherapy, prevent tumor recurrence and metastasis, and strengthen the patient’s ability to combat the disease. This integrated treatment not only improves the patient’s overall quality of life but also extends their lifespan. Furthermore, sensitivity analysis supports the appropriateness of the weight distribution of these indicators, thereby enhancing the accuracy and credibility of the research findings.

### 4.5 Bias

Despite the comprehensive literature review and the establishment of stringent inclusion and exclusion criteria, several limitations persist in this study. On one hand, the quality of the included studies is relatively low; most did not provide sufficient details regarding randomization methods, blinding procedures, or allocation concealment. Furthermore, the sample sizes were small, indicating a need for larger RCTs in the future to enhance the quality of the evidence(R2-P17). On other hand, In Multi-Criteria Decision Analysis (MCDA), weight assignment is a crucial step in evaluating and comparing different options. Common weighting methods include the Analytic Hierarchy Process (AHP), Delphi method, Swing Weighting method, and Discrete Choice Experiments (DCE), among others. Each of these methods has its own advantages and applicable scenarios, but they share the common characteristic of relying on the subjective judgment or preferences of stakeholders to determine the weights and priorities, which introduces certain inherent limitations (45)(R2-P12/19).

## 5 Conclusion

In conclusion, the integration of Fuzheng Yiliu Decoction with chemotherapy presents a more effective treatment regimen for non-small cell lung cancer (NSCLC) than chemotherapy alone. This combined approach not only enhances the quality of life for NSCLC patients but also effectively alleviates the toxic side effects typically associated with conventional treatments. Furthermore, this study utilizes real-world clinical data, and the multi-criteria decision analysis (MCDA) model provides a robust framework for benefit-risk evaluation. The conclusions were reached after several iterations of simulation, indicating the reliability of the findings. These results may offer valuable insights for clinical practice.

## Data Availability

The original contributions presented in the study are included in the article/**Supplementary Material**. Further inquiries can be directed to the corresponding author.
