# Supplementary material for: Benefit-risk evaluation of Fuzheng Yiliu Decoction combined with chemotherapy for treating non-small cell lung cancer using multicriteria decision analysis
Source: Front Oncol. 2025 Sep 23;15:1627904. doi: 10.3389/fonc.2025.1627904 (PMC12500661; doi:10.3389/fonc.2025.1627904)
Supplement: Supplementary file 2 [file Supplementaryfile2.zip › supplementary files/title page.docx]

**Title**

Benefit-risk evaluation of Fuzheng Yiliu Decoction combined with chemotherapy for treating non-small cell lung cancer using multicriteria decision analysis(MCDA)

**Running heading**

Benefit-risk evaluation of traditional Chinese medicine combined with chemotherapy for treating non-small cell lung cancer

**Author information**

The names of the authors:

Dongjing Ma^1,2^, Yingying Yang^2^, Di Yan^2^, Keqin Gao^2^, Shenwen He^2^, Jinbin Wang^2^, Yuanchao Zhang^3^*, Jianjun Wu^1,2^*

1 The Collaborative Innovation Center for Prevention and Control by Chinese Medicine on Diseases Related Northwestern Environment and Nutrition, Gansu University of Chinese Medicine, Lanzhou, 730000, Gansu, China

2 School of Public Health, Gansu University of Chinese Medicine, Lanzhou, 730000, Gansu, China

3 School of Finance, Lanzhou University of Finance and Economics, Lanzhou 730101, Gansu, China

Dongjing Ma and Yingying Yang have contributed equally and shared co-first authorship to this study.

Corresponding authors:

Jianjun Wu, E-mail address: wjj2118@126.com

Dongjing Ma (Orcid ID : 0000-0002-3222-9550)

**Abstract**

**Introduction** This study employs multi-criteria decision analysis (MCDA) models to evaluate the benefits and risks associated with the combination of Fuzheng Yiliu Decoction and chemotherapy for non-small cell lung cancer (NSCLC). The aim is to enhance the rational use of medications in clinical practice while also providing policy consulting and support services for traditional Chinese medicine in the prevention and management of chronic diseases at the grassroots level.

**Methods** A comprehensive literature search was conducted in PubMed, Web of Science, China National Knowledge Infrastructure (CNKI), China Biology Medicine Disc (CBM), Wanfang Databases, and China Science and Technology Journal Database (VIP Databases) to identify relevant studies on Fuzheng Yiliu Decoction combined with chemotherapy for NSCLC. Meta-analysis using RevMan 5.3 was performed to compare the effect sizes of the two treatment regimens. A MCDA model was developed to construct a value tree based on benefit-risk indicators. The benefit value, risk value, and benefit-risk ratio for both treatments were calculated using Hiview 3.2 software, followed by sensitivity analysis to assess result robustness. Monte Carlo simulations were performed using Oracle Crystal Ball 11.1 software to optimize the evaluation outcomes.

**Results** The literature search identified 6 randomized controlled trials (RCTs) comparing chemotherapy alone with chemotherapy combined with Fuzheng Yiliu Decoction. The MCDA model showed that the combination therapy had significantly higher benefit values (72) compared to chemotherapy alone (29). The risk value for combination therapy (56) was slightly higher than that of chemotherapy alone (24), but the overall benefit-risk value for combination therapy (68) was notably greater than chemotherapy alone (27). Monte Carlo simulations revealed a difference in total efficacy-risk values between the two treatments of 41 (95% CI: -16.59, 38.73). The probability that the combination therapy’s benefit-risk value exceeds that of chemotherapy alone was 81.83%.

**Discussion** These findings suggest that combining Fuzheng Yiliu Decoction with chemotherapy improves therapeutic efficacy and reduces chemotherapy’s adverse side effects, offering a promising treatment strategy for NSCLC. This study provides valuable insights into enhancing treatment strategies and clinical decision-making in managing NSCLC.

**Keywords** Fuzheng Yiliu Decoction，non-small cell lung cancer, Combined therapy, multicriteria decision analysis（MCDA）

**List of abbreviations**

| **Abbreviation.** | **Whole process.** |
| --- | --- |
| NSCLC | Non-small cell lung cancer |
| TCM | Traditional Chinese Medicine |
| MCDA | Multi-Criteria Decision Analysis |
| TOPSIS | Technique for Order of Preference by Similarity to Ideal Solution |
| CUI | Clinical Utility Index |
| CNKI | China National Knowledge Infrastructure |
| CBM | China Biology Medicine Disc |
| RCT | Randomized controlled trial |
| KPS | Karnofsky Performance Status |
| CEA | Carcino embryonic antigen |
| CI | Confidence intervals |
| BRA | Benefit-risk assessment |
| IPF | Idiopathic progressive pulmonary fibrosis |

**Declarations**

**Funding** This work was supported by the Open Fund of the Collaborative Innovation Center for Prevention and Control by Chinese Medicine on Diseases Related Northwestern Environment and Nutrition (ZYXT-24-09), 2022 Gansu Provincial Colleges and Universities Innovation Fund Project (2022B-119) and Gansu Provincial Natural Science Foundation Youth Science and Technology Fund (23JRRA1726).

**Competing interests** The authors declare that they have no known competing financial interests or personal relationships that could have appeared to influence the work reported in this paper.

**Availability of data and materials** Not applicable.

**Ethics approval and consent to participate** Not applicable.

**Consent for publication** Not applicable.

**Author contributions** YYY was responsible for writing the manuscript of the article, MDJ was responsible for reviewing and revising the manuscript, GKQ and WJB were responsible for checking and revising the format, YD and HSW were responsible for the arrangement and production of the icons, and ZYC and WJJ were responsible for the final review and revision.All authors approved the final version of the article.

**Acknowledgements** The authors extend their heartfelt gratitude to all participants for their
valuable cooperation and to their support.
