# Supplementary material for: Benefit-risk evaluation of Fuzheng Yiliu Decoction combined with chemotherapy for treating non-small cell lung cancer using multicriteria decision analysis
Source: Front Oncol. 2025 Sep 23;15:1627904. doi: 10.3389/fonc.2025.1627904 (PMC12500661; doi:10.3389/fonc.2025.1627904)
Supplement: Supplementary file 2 [file Supplementaryfile2.zip › supplementary files/Included literature.zip!/literature/纳入文献/1张伟.pdf]

rhG－CSF 及口服升白细胞药物对副作用的干预效果如表 2：

表 2 rhG－CSF 及口服升白细胞药物对副作用的干预效果

干预情况	干预例数(n)	一周有效率(n/%)	一月有效率(n/%)
rhG－CSF	39	34(87.2)	4(10.3)
升白细胞药物	42	3(7.1)	28(66.7)
p	－	＜0.05	＜0.05

从表 2 中可以看出，采用 rhG－CSF 干预，一周有效率为 87.2%，一月有效率为 10.3%。采用口服升白细胞药物干预，一周有效率为 7.1%，一月有效率为 66.7%，两组数据对比统计学差异显著(p＜0.05)。

3 讨论

干扰素治疗慢性病毒性肝炎，初期容易导致患者血液内 WBC 指标迅速下降，判断属于治疗所带来的骨髓抑制副作用[3]。治疗结束后，WBC 指标能够有所恢复。本文研究发现，长效组患者 WBC 转阴率为 50%，较常规组患者转阴率(26.8%)更高，认为长效干扰素用于治疗慢性病毒性感染，耐受性与依从性更强。

白细胞减少时，可采用 rhG－CSF 及口服升白细胞药物对病情加以干预，促进 Nec 新生细胞产生，减轻骨髓抑制副作用对患者预后的影响[4]。本文研究发现，采用 rhG－CSF 干预，一周有效率为 87.2%，一月有效率为 10.3%。采用口服升白细胞药物干预，一周有效率为 7.1%，一月有效率为 66.7%。两者对比统计学差异显著。rhG－CSF 起效更快，但药效不够持久，有研究表明，部分患者在用药 1 月后，白细胞又恢复到了原来的水平，且存在食欲下降等不良反应。相对于 rhG－CSF 而言，口服白细胞药物起效慢，但疗效稳定，且药物价格相对低廉。如发现患者在治疗过程中出现副作用，应首先考虑给予口服升白细胞药物治疗，若非必要，应避免采用 rhG－CSF 药物治疗，以降低患者用药费用。

结论

长效干扰素用于治疗慢性病毒性肝炎，疗效更好，转阴率更高，副作用更小。如发现副作用，可采用口服升白细胞药物干预，减轻患者用药经济负担，提高干预的长期疗效，提高患者治疗有效率。

参考文献

[1]林宗梅，王蓓蓓，林秀芳，陈春雷. 干扰素治疗慢性乙型肝炎的临床效果及影响因素分析[J]. 中国生化药物杂志，2014，02：109－110.

[2]扈晓宇，钟森，陈婧，李晖. 小柴胡汤联合干扰素治疗慢性乙型肝炎疗效与安全性的系统评价[J]. 中国循证医学杂志，2011，03：308－313.

[3]梁雪梅，赖观好. 干扰素治疗慢性乙型肝炎的教育指导[J]. 当代医学，2011，17：130－131.

[4]张艳. 聚乙二醇干扰素 α－2a 联合利巴韦林治疗慢性病毒性丙型肝炎的效果及护理[J]. 现代临床护理，2011，07：45－47.

冠心病患者 128 层螺旋 CT 冠状动脉成像斑块相关性研究

王升广　刘丽媛　刘艳梅

（河北邢台县医院　054000）

【摘要】 目的：探究冠心病患者 128 层螺旋 CT 冠状动脉成像斑块成分与狭窄的相关性。方法：选取 100 例冠心病患者为研究对象，给予 128 层螺旋 CT 冠状动脉检查，分析斑块成分与冠状动脉狭窄程度的相关性。结果：100 例患者中，70 例为不同程度的冠状动脉狭窄，硬斑块狭窄 6 例，软斑块狭窄 53 例，混合型斑块狭窄 11 例。结论：128 层螺旋 CT 可以有效检查出冠状动脉成像板块成分以及狭窄程度，对于冠心病的诊断和早期治疗具有重大意义。

【关键词】 冠心病；128 层螺旋 CT；冠状动脉狭窄程度

冠状动脉粥样硬化性心脏病简称为冠心病，是由于冠状动脉功能性或者器质性病变而导致的冠状动脉供血和心肌的需求量不平衡所导致的心肌损害。随着我国人们生活压力的加大以饮食习惯等改变使得冠心病患者在逐渐增加。近年研究表明，早期活动性的冠状动脉粥样硬化病变与存在潜在破裂性的斑块具有相关性[1]。因此早期发现易破裂的斑块对于早期治疗冠心病意义重大。本次我们采用 128 层螺旋 CT 冠状动脉成像的方法，初步探讨冠状动脉斑块的成分和狭窄程度的相关性。

1 资料与方法

1.1 资料

选择 2013 年 8 月至 2016 年 8 月期间我院收治的 100 例冠心病患者，男性患者 59 例，女性患者 41 例，年龄(58.6±11.0)岁，心率(70.0±12.0)次/min。纳入标准：自愿接受本次检查，并与我方签订知情协议；碘试显示无过敏。排除标准：肝肾功能异常、心律不齐、无法支配呼吸屏气患者。

1.2 方法

1.2.1 扫描方法

128 层螺旋 CT，仪器为飞利浦 CT 机，120KV，有效 MAS：176mA，旋转时间设置为 0.33s/r，采集 128mmX0.6mm，厚度为 3.0mm，间隔设置为 0.6mm。扫描范围：气管隆嵴下方 1cm 至膈下 2cm 左右，注射器为双筒压力型，肘前正中静脉以 5ml/s 的速度注射 370mg/ml 的碘帕醇 70ml，和 40ml 的生理盐水，增加扫描冠状动脉。

1.2.2 图像处理

由 2 名经验丰富的诊断医生对图片进行单独的阅读，根据获得的原始图像冠状动脉支显示情况，分别重建 40%、45%、50%、70%、75%、80%R－R 期间图像。并采用 GEAW4.2 工作站对图片进行三维重建，用容积再现、最大密度投影、曲面层面重建以及回顾心电门控技术显示冠状动脉树以及冠状动脉 1－13 段的分支情况[2]。

1.3 观察项目

统计狭窄情况，软斑块、硬斑块、混合性斑块数量等。

2 结果

100 例患者中，30 例无冠状动脉狭窄，比例为 30.0%，70 例为不同程度的冠状动脉狭窄比例为 70%，其中轻度狭窄 32 例(45.7%)，中度狭窄 21 例(30.0%)，重度狭窄 17 例(24.3%)。

70 例冠状动脉狭窄患者中硬斑块狭窄 6 例(8.5%)，其中轻度狭窄 3 例，中度狭窄 1 例，重度狭窄 2 例；软斑块狭窄 53 例((75.7%)，其中轻度狭窄 25 例，中度狭窄 15 例，重度狭窄 13 例；混合型斑块狭窄 11 例(15.8%)，其中轻度狭窄 4 例，中度狭窄 5 例，重度狭窄 2 例。

3 讨论

冠状动脉狭窄的病理基础是以动脉粥样硬化斑块破裂以及血栓的形成，随着病情的及不断恶化出现急性冠状动脉综合征比如心绞痛、急性心肌梗死等。研究表明早期和活动期的冠状动脉粥样硬化的病变和存在斑块的缺损情况息息相关[3]，而软斑块对于疾病的危害较大。因此治疗冠心病应从早发现冠状动脉易损斑块入手。动脉粥样硬化是累及体循环系统的动脉内膜疾病，其特征是动脉内膜斑块的形成，每个斑块的组成成分不同。冠状动脉粥样硬化性斑块的破裂和继发的血栓形成则是急性冠脉综合征的直接原因。而斑块破裂是最常见的斑块并发症类型，占致死性及急性心肌梗死和冠状动脉猝死的 70%[4－5]。

128 层螺旋 CT 冠状动脉造影可以准确的显示冠状动脉的影像，准确区分冠状动脉的软、硬斑块，可以快速判断冠状动脉的通畅性和狭窄程度[6－7]，具有微创性以及视角观察的优点，是评价冠状动脉病变的重要参考和筛选方法[8]。因此本次我们采用 128 层螺旋 CT 冠状动脉成像探究冠心病中斑块成分与狭窄的相关性，结果显示不同狭窄程度的冠状动脉其斑块的软、硬以及混合斑块的分布情况不一致，从而进一步说明斑块的缺损程度与冠状动脉的狭窄程度息息相关。

总而言之，128 层螺旋 CT 冠状动脉造影是一种安全、无创的血管检查方法，在检查时候近需要注入对比剂并在短时间内可以进行较大范围的扫描，但是受到一些技术等因素的影响，在实际工作中还需要做好检查的准备工作，尽量做到正确的评估，为患者的健康带来多一份保证。最后通过本次的临床探究我们一致认为：128 层螺旋 CT 可以有效检查出冠状动脉成像板块成分以及狭窄程度，对于冠心病的诊断和早期治疗具有重大意义。

参考文献

[1] 杨晓斌. 冠心病患者 64 层螺旋 CT 冠状动脉成像斑块成分与狭窄程度的相关性探讨[J]. 中国现代药物应用，2016，10(6)：37－38.

[2] 王昕，武蓉，石峰，等 . 64 层螺旋 CT 在冠状动脉狭窄诊断中的应用价值 . 中国中西医结合影像学杂志 ，2010，8(2)：108－110，113.

[3] 连舟洋 . MSCT 评价广州地区无症状自然人群冠状动脉斑块分布特征及其危险因素分析 . 南方医科大学 ，2010.

[4] 谢元亮，金朝林，张树桐. Meta 分析 64 层螺旋 CT 对冠状动脉狭窄或阻塞的诊断价值[J]. 中华老年心脑血管病杂志，2008，10(3)：173－174.

[5]曾少良，汤文善，金勤剑，等. 64 层螺旋 CT 冠状动脉成像斑块与冠心病相关性分析[J]. 中国现代医生，2013，51(20)：78－79.

[6] 薛松. 128 层螺旋 CT 在冠状动脉不同程度狭窄中的临床诊断价值[J]. 医药论坛杂志，2011，32(24)：8－9.

[7] 盛杰鑫，王西宾，薛斌，等. 128 层螺旋 CT 冠状动脉成像对诊断冠心病的价值[J]. 现代医卫生，2012，28(13)：1944－1946.

[8] 甄杰，高杰，张明. 冠状动脉粥样硬化斑块的影像学诊断比较及临床应用价值[J]. 华北国防医药，2009，21(4)：54－56.

晚期非小细胞肺癌患者化疗相关性疲乏的中医治疗分析

张伟

（涿州市中医医院　072750）

【摘要】 目的：出现化疗相关性疲乏的晚期非小细胞肺癌患者在通过中医方法进行治疗时所取得的临床成效。方法：择取我院 2012 年 09 月－2016 年 09 月入院就诊的晚期非小细胞肺癌化疗相关性疲乏之患者 45 例(观察组)，给予他们中医扶正抑瘤汤治疗。同时，选取同期的同病症患者 45 例(对照组)，仅给予他们单纯的西医化疗治疗。对两组的疲乏情况和临床疗效进行分析、对比和统计。结果：在治疗后癌性疲乏评分方面，观察组患者(24.86±10.28)同对照组患者(36.14±9.28)相比明显偏低；在临床治疗总有效率方面，两组患者(95.56%，71.11%)差异也存在明显可比性(P＜0.05)，存在一定的统计学意义。结论：晚期非小细胞肺癌患者在进行化疗相关性疲乏的诊治时，通过采用扶正抑瘤的治疗成效显著，在改善患者疲乏症状的同时，使临床治疗效果明显提升，应在临床大力推广应用。

【关键词】 晚期非小细胞肺癌；化疗；相关性疲乏；扶正抑瘤汤；临床效果

目前，化疗是晚期非小细胞肺癌治疗的一种主要方法，但与此同时，随着化疗的不断开展，许多患者都容易出现躯体、认知、情感等方面的疲乏，不仅对临床治疗造成一定的影响，也给患者的生命健康以及生存质量造成较大的损害[1－2]。因此，必须加强对化疗相关性疲乏的临床治疗。下面，文章随机抽选于 2012 年 09 月－2016 年 09 月在我院接受治疗的晚期非小细胞肺癌化疗相关性疲乏之患者 45 例作为观察组，并另抽选患者 45 例作为对照组。分别进行中医治疗以及单纯西医化疗治疗。现将详细情况汇报如下。

1 资料与方法

1.1 一般资料 抽选我院内科 2012 年 09 月－2016 年 09 月期间收治的患有化疗相关性疲乏之症的晚期非小细胞肺癌患者 45 例(观察组)。患者的年龄约是 44 岁至 79 岁之间，平均年龄 61.5±4.5 岁；女性和男性患者分别是 26 人和 19 人；病程时间约是 5 个月至 10 个月之间，平均病程时间约 6.6±1.2 个月。另选取 45 例同时期

万方数据

的同病症患者(对照组),其年龄约是44岁至79岁之间,平均年龄约62.0±4.7岁;女性和男性患者分别是25人和20人;病程时间约是5个月至10个月之间,平均病程时间约6.8±1.0个月。经过甄别诊断,两组患者各方面的基本资料对比均无明显差异,不存在统计学意义(P>0.05)。

1.2 方法 给予对照组单纯西医方法治疗。即对患者进行TP方案进行化疗治疗,连续治疗6个星期(3个星期为一周期)。观察组的治疗方法是TP化疗+扶正抑瘤汤治疗。主要方法是:患者在进行TP化疗期间服用中药扶正抑瘤汤,具体药方是:白花蛇舌草、生薏苡仁、党参、半枝莲、炒谷芽、茯苓、虎杖、黄芪、砂仁、枳壳、白术、绿萼梅、厚朴。冷水煎煮,1剂/d,2次/d(早、晚服用),连续用药6个星期。

1.3 观察项目 (1)观察两组治疗前后的躯体、认知、情感疲乏评分;(2)对比两组的临床治疗情况。

1.4 疗效评价 (1)显效,即患者经过治疗后,肿瘤病情稳定,躯体、认知、情感等方面的疲乏症状基本消失或明显减轻,心理状况良好,生活质量明显改善;(2)有效,即患者经过治疗后,肿瘤病情基本稳定,其各方面疲乏症状有所减轻,心理状况较好,生活质量有所改善;(3)无效,即患者经过治疗后,肿瘤病情有恶化趋势,各方面疲乏症状无减轻,心理状况较差,生活质量无改善[3]。

1.5 统计学分析 采用SPSS17.0系统软件统计分析资料;T检验表述计量资料,X^2检验表述计数资料;当比较差异P<0.05时,则结果具有统计学意义。

2 结果

2.1 两组的相关性疲乏评分分析

治疗前的癌性疲乏评分方面,两组患者的评分均较高,水平基本持平(P>0.05),不存在统计学意义。治疗后的躯体疲乏、认知疲乏、情感疲乏以及总癌性疲乏评分方面,观察组患者的评分有明显下降,且两组之间的差异对比性较大(P<0.05),存在一定的统计学意义。详见表1。

表1 两组患者的癌性疲乏评分比较

组别		对照组	观察组	T值	P值
例数(n)		45	45	—	—
躯体疲乏	治疗前	17.70±4.98	17.72±4.79	0.08114	>0.05
	治疗后	16.74±4.97	12.06±5.12	4.81624	<0.05
认知疲乏	治疗前	10.53±2.07	10.61±2.20	0.10772	>0.05
	治疗后	9.50±3.81	6.41±2.04	4.53538	<0.05
情感疲乏	治疗前	11.64±4.06	11.67±4.12	0.05031	>0.05
	治疗后	10.20±3.05	6.98±2.53	4.59930	<0.05
总评分	治疗前	39.85±10.62	39.94±9.95	0.10578	>0.05
	治疗后	36.14±9.28	24.86±10.28	6.10207	<0.05

2.2 两组的临床治疗效果分析

统计学分析显示,观察组60例患者中,痊愈患者31例(约占51.67%),其临床治疗总有效率为95.0%(57/60);对照组30例患者中,痊愈患者6例(约占20.0%),其临床治疗总有效率为70.0%(21/30)。观察组明显高于对照组(P<0.05),存在一定的统计学意义。详见表2。

表2 两组的临床疗效比较

组别	对照组	观察组	X^2值	P值
例数(n)	45	45	—	—
显效(n,%)	21(46.67)	37(82.22)	5.85639	<0.05
有效(n,%)	11(23.91)	6(13.33)	3.72857	>0.05
无效(n,%)	13(28.89)	2(4.44)	4.53174	<0.05
总有效率(n,%)	32(71.11)	43(95.56)	4.53174	<0.05

3 讨论

癌性疲乏是肿瘤患者在化疗过程中以及化疗后经常出现的一种并发症,据有关部门不完全统计,它的发病率高达70%-100%,对患者生活质量和生命健康有严重不良影响[3]。经过大量临床研究显示,癌性疲乏的发病机制主要同化疗造成的胃肠道反应、肝功能损害以及骨髓抑制等有关,但目前西医尚没有较佳的临床治疗方案[4]。中医临床认为,出现化疗癌性疲乏的晚期非小细胞肺癌患者多属于脾胃虚弱、气血不足等导致的,因此在临床治疗时应该以健胃健脾、补虚益气为主。扶正抑瘤汤中的白花蛇舌草、党参、生薏苡仁、炒谷芽、茯苓、黄芪、白术等经过混合联用,能够起到有效的健胃、消食、健脾、益气、补虚、固本等作用,从而有效缓解患者因化疗造成的不良反应[5-6]。本文通过探究晚期非小细胞肺癌化疗相关性疲乏患者通过扶正抑瘤汤治疗后取得的临床成效,研究数据显示,服用扶正抑瘤汤患者癌性疲乏总评分(24.86±10.28)和临床总疗效(95.56%)均明显优于单纯NP化疗患者(36.14±9.28,71.11%),综上所述,晚期非小细胞肺癌化疗相关性疲乏患者通过中医治疗后可以取得显著地临床效果,在明显减轻患者躯体、认知、情感等疲乏程度的同时,使患者生活质量明显改善,应广泛推广应用。

参考文献:

[1]成娅婷,田成旺,张铁军等.中药治疗非小细胞肺癌的临床应用及作用机制研究进展[J].药物评价研究,2016,39(02):293-299.

[2]孔祥应,赵明丹,黄桂军等.扶正抑瘤汤治疗晚期非小细胞肺癌患者化疗相关性疲乏的临床观察[J].北方药学,2016,13(06):85-86.

小柴胡汤加减治疗功能性消化不良的临床观察

陈海艳

(北京市昌平区南邵社区卫生服务中心 102200)

【摘要】 目的:探讨小柴胡汤加减治疗功能性消化不良的临床观察。**方法:**选取我院2015年1月-2016年1月的患有功能性消化不良的76例患者,将其随机分为对照组和观察组,对其分别采用常规西药和小柴胡汤加减治疗,评价治疗效果。**结果:**治疗后,观察组和对照组的治疗总有效率分别为92.1%,89.5%,差异不明显(P>0.05),无统计学意义。随访一个月后,观察组患者的治疗总有效率为89.5%,对照组患者的治疗总有效率为73.7%,存在显著差异(P<0.05),有统计学意义。**结论:**小柴胡汤加减治疗功能性消化不良的临床效果显著,减轻患者的临床症状,缓解患者的病情,可在临床上广泛使用。

【关键词】 小柴胡汤;功能性消化不良;治疗效果

功能性消化不良是临床上常见的一种非器质性疾病,属于消化系统疾病的一种,其临床症状有饱胀、食欲不振、呕吐、嗳气、上腹痛、恶心等,影响患者的身体健康。临床上现代医学对于该病的治疗主要采用常规西药治疗,长期临床实践发现其临床效果不明显,中医认为功能性消化不良属于"胃脘痛"、"痞满"等范畴,本次研究采用小柴胡汤加减治疗,随着患者的病情进行药物剂量以及次数的调整,针对性的治疗疾病,取得有效的临床效果,以下是本次研究报告。

1 资料与方法

1.1 一般资料

选取我院2015年1月-2016年1月的患有功能性消化不良的76例患者作为研究对象,其中男性患者40例,女性患者36例,所有患者均符合功能性消化不良的诊断标准,即根据《中药新药临床研究指导原则》对患者采用内窥镜检查,均无胃及十二指肠、肿瘤以及糜烂,无食管炎,均自愿参加本文的研究调查。排除标准:精神病、糖尿病、肝胆器质性疾病、结缔组织疾病以及腹部手术史的患者。对照组中男性21例,女性17例,年龄26-49岁,平均年龄(36.2±5.1)岁,病程1-4个月,平均病程(1.9±0.2)个月,体重45-82Kg,平均体重为(52.3±6.1)Kg,观察组中男性22例,女性16例,年龄25-47岁,平均年龄(36.5±5.8)岁,病程1-3个月,平均病程(1.8±0.4)个月,体重46-83Kg,平均体重为(53.4±5.7)Kg,对照组和观察组患者的一般资料没有差异(P>0.05),可进行比较研究。

1.2 方法

对照组患者采用常规西药治疗,给患者餐前0.5h口服多潘立酮10mg/次,3次/d,同时服用雷尼替丁200mg/次,3次/d,谷维素30mg/次,3次/d。观察组采用小柴胡汤加减治疗,药物组成:柴胡、半夏、白芍各15g,白术20g,黄芩、党参、枳实、红枣、桔梗各10g,炙甘草6g,生姜2片,每天水煎服1剂,分早晚服用。两组均连续治疗1个月,并随访1个月,观察其临床效果。

1.3 评价指标

显效:患者的上腹痛、饱胀、食欲不振等临床症状全部消失;有效:患者的上腹痛、饱胀、食欲不振等临床症状有所缓解;无效:临床症状没有改变甚至加重。总有效率=治愈率+好转率。

1.4 统计学方法

采用SPSS19.0统计学软件对本文中的数据进行分析,组间率对比采取X^2检验,计数资料采用%表示,以P<0.05表示结果差异明显,具有统计学意义。

2 结果

治疗后,观察组和对照组的治疗总有效率分别为92.1%,89.5%,差异不明显(P>0.05),无统计学意义。随访一个月后,观察组患者的治疗总有效率为89.5%,对照组患者的治疗总有效率为73.7%,存在显著差异(P<0.05),有统计学意义。

表1 两组患者随访一个月的临床疗效比较[n(%)]

组别	例数	显效	有效	无效	总有效率
观察组	38	24(63.2)	10(26.3)	4(10.5)	34(89.5)
对照组	38	16(42.1)	12(31.6)	10(26.3)	28(73.7)
X^2	--	9.31	1058	8.07	8.07
P	--	<0.05	<0.05	<0.05	<0.05

3 讨论

功能性消化不良是一种临床综合征,其临床表现有上腹痛、恶心呕吐、嗳气、饱胀等,对患者的身体造成伤害,如果不及时治疗,病情一直反复出现,影响患者的身体健康。功能性消化不良的发病机制尚不明确,西医临床认为该病的病理基础是胃肠道动力障碍,并且与精神以及应激因素有关[1]。因此,临床上治疗该病采用的是多潘立酮,具有改善患者胃动力的功效,雷尼替丁具有抑酸作用可有效缓解患者的临床症状,谷维素具有调节患者的神经、抵抗焦虑以及缓解患者的紧张情绪的作用。但是长期临床发现,其治疗效果并不明显。传统中医认为该病属于胃脘痛、痞满等范畴[2],治疗的关键在于气机引起的功能性紊乱郁滞,患者受到外邪侵袭或者是内伤七情,均可引起肝胆气机郁结不畅,对患者的肠胃纳运功能有影响,从而导致患者中焦,气机不畅,胃失和降继而引起功能性消化不良。因此,中医上认为治疗该病首先应从疏利肝胆着手,本文中采用的治疗方法是中医八法中的"和法",方药是小柴胡汤加减,柴胡和黄芩并用具有舒畅气机的作用,党参、白术、红枣、炙甘草并用有益气健脾和中的功效,桔梗和枳实具有调达升降的作用,生姜和半夏具有辛散开结、和胃降逆之功,诸药联合使用具有开郁散结、舒畅气机、健脾和胃的功效[3]。同时也可根据患者的病情变化加减药物的剂量,使药物能够发挥最大的作用,促进患者病情的康复。本文中对功能性消化不良的76例患者采用常规西药和小柴胡汤加减治疗后,观察组和对照组的治疗总有效率分别为92.1%,89.5%,差异不明显(P>0.05),无统计学意义。随访一个月后,观察组患者的治疗总有效率为89.5%,对照组患者的治疗总有效率为73.7%,存在显著差异(P<0.05),有统计学意义,说明采用小柴胡汤加减治疗功能性消化不良的患者,远期疗效较好,能够明显改善患者的肠道,提高患者的临床效果和生活质量。

综上所述,小柴胡汤加减治疗功能性消化不良的临床效果显著,减轻患者的临床症状,缓解患者的病情,促进其早日康复,可在临床上广泛使用。

参考文献

[1]聂琼芳.小柴胡汤加减治疗功能性消化不良38例临床观察[J].时珍国医国药,2016,16(5):410-410.

[2]李密.小柴胡汤加减治疗功能性消化不良46例体会[J].中外女性健康研究,2016,10(18):27,34.

[3]陈亚平,章浩军,罗颖等.小柴胡汤加减治疗功能性消化不良临床观察[J].中医药通报,2015,5(4):52-53.

万方数据
