# Supplementary material for: Benefit-risk evaluation of Fuzheng Yiliu Decoction combined with chemotherapy for treating non-small cell lung cancer using multicriteria decision analysis
Source: Front Oncol. 2025 Sep 23;15:1627904. doi: 10.3389/fonc.2025.1627904 (PMC12500661; doi:10.3389/fonc.2025.1627904)
Supplement: Supplementary file 2 [file Supplementaryfile2.zip › supplementary files/Included literature.zip!/literature/纳入文献/3孙艾琳.pdf]

学校代码：10228
学　　号：201622289

硕士学位论文

（专业型硕士学位论文）

扶正抑瘤汤治疗气虚血瘀型非小细胞肺癌化疗患者的临床观察

CLINICAL OBSERVATION OF FUZHENG YILIU DECOCTION ON CHEMOTHERAPY OF NON-SMALL CELL LUNG CANCER WITH QI DEFICIENCY AND BLOOD STASIS

专　　业：中西医结合临床
研究生：孙艾琳
导　　师：刘松江　教授

黑龙江中医药大学

中国知网　https://www.cnki.net

目 录

缩略词表 1
中文摘要 2
Abstract 4
前　言 7
文献综述 8
1.肺癌的现 代医学研究进展 8
1.1 外科手术治疗（surgical treatment） 8
1.2 化学治疗（Chemotherapy） 8
1.3 放射治疗（Radiotherapy） 9
1.4 靶向治疗（Targeted therapy） 9
1.5 其他治疗方法 9
2.肺癌的中医药学研究进展 10
2.1 古代中医学对肺癌病名的阐述 10
2.2 肺癌的病因病机 10
2.3 肺癌的辨证分型 11
2.4 肺癌的中医治疗 13
2.5 肺癌的中西医结合治疗 14
临床观察 18
1.研究资料 18
1.1 病例来源 18
1.2 诊断标准 18
1.3.纳入标准 19
1.4.排除标准 19
1.5 脱落、剔除标准 20
2.研究对象及方法 20
2.1 研究对象 20
2.2 研究方法 21
3.观察指标 22
3.1 疗效性指标 22

中国知网 https://www.cnki.net

3.2 疗效评价标准 22
4.统计学方法 23
结　果 24
1.两组病例治疗前后血清 CEA、CA211 的比较结果 24
2.两组病例治疗前后中医证候积分疗效比较 25
3.两组病例治疗前后 KPS 评分比较 26
4.两组病例治疗前后不良反应比较 27
讨　论 28
1.立法依据 28
2.扶正抑瘤汤对气虚血瘀型非小细胞肺癌作用的探讨 28
2.1 方药组成 28
2.2 组方特点 29
2.3 现代药理研究 30
3.疗效分析 33
3.1 对血清肿瘤标志物 CEA、CA211 变化的分析 33
3.2 对中医证候改善的分析 33
3.3 对 KPS 评分改善的分析 34
3.4 对毒副反应的分析 34
4.问题与展望 34
结　论 36
致　谢 37
参考文献 38
附　录 41
原创性声明 46
使用授权声明 46
个人简历 47

中国知网 https://www.cnki.net

缩略词表

缩略词	英文名称	中文名称
CEA	Carcino-embryonic antigen	癌胚抗原 CEA
CA211	carbohydrate antigen211	糖类抗原 211
CA125	carbohydrate antigen125	糖类抗原 125
CA153	carbohydrate antigen153	糖类抗原 153
CREA	creatinine	肌酐
GPT	glutamic-pyruvic transaminase	谷丙转氨酶
HB	hemoglobin	血红蛋白
NSCLC	nonsmall-cell lung cancer	非小细胞肺癌
PLT	platelet	血小板
WBC	white blood cell	白细胞

中国知网 https://www.cnki.net

中文摘要

目的：

本研究是以中医药理论为指导，通过临床对照试验研究，观察扶正抑瘤汤对气虚血瘀型非小细胞肺癌化疗患者在抑制肿瘤生长、改善临床症状等方面产生的影响，以期以统计学方法了解其在这些方面存在的积极作用是否具有统计学意义，进而为该方剂广泛应用于非小细胞肺癌的临床治疗提供更多更详实的理论依据。

方法：

选取符合纳入标准的气虚血瘀型非小细胞肺癌患者 60 例，随机分为对照组 30 例和治疗组 30 例，对照组采取 DP（多西他赛+顺铂）方案化疗，化疗 4 周期，治疗组采用 DP 方案化疗 4 周期，同时口服扶正抑瘤汤，21 天为 1 周期，连续治疗 4 周期后，对比两组患者血清 CEA、CA211、中医证候评分、生存质量评分、相关毒副反应及安全指标（血常规、肝功、肾功等）的变化情况。使用 SPSS 软件进行统计学数据处理。

结果：

治疗期间因脱落 3 例，共收集符合标准病例 57 例。

1、扶正抑瘤汤联合化疗与单纯化疗后患者的 CEA、CA211 水平均较治疗前降低，差异具有统计学意义（$P<0.05$）；且治疗组患者的 CEA、CA211 降低水平高于对照组，差异具有统计学意义（$P<0.05$）；

2、扶正抑瘤汤联合化疗与单纯化疗后患者的中医证候均较治疗前改善，差异具有统计学意义（$P<0.05$）；且治疗组患者的中医证候缓解水平高于对照组，差异具有统计学意义（$P<0.05$）；在 KPS 评分上，治疗组和对照组在治疗后均有所改善，且治疗组的改善程度明显高于对照组，差异均具有统计学意义（$P<0.05$）；

3、扶正抑瘤汤联合化疗与单纯化疗后患者在白细胞、血小板、血红蛋白下降方面，治疗组明显轻于对照组，差异具有统计学意义（$P<0.05$）；对胃肠道反应治疗后治疗组明显优于对照组，差异具有统计学意义（$P<0.05$）；在肝肾功能方面，治疗后治疗组与对照组相比，差异不具有统计学意义（$P>0.05$）。

中国知网 https://www.cnki.net

结论：

1、扶正抑瘤汤联合化疗能明显降低气虚血瘀型非小细胞肺癌患者血清肿瘤标志物 CEA、CA211 的水平；

2、扶正抑瘤汤能够改善气虚血瘀型非小细胞肺癌化疗患者的中医证候，并提高其生存质量；

3、扶正抑瘤汤能够改善气虚血瘀型非小细胞肺癌化疗患者的白细胞、血小板、血红蛋白计数的下降及消化道不良反应，对肝肾功能未见明显影响。

关键词： 非小细胞肺癌

气虚血瘀型

多西他赛+顺铂

扶正抑瘤汤

中国知网 https://www.cnki.net

Abstract

Objective:

This study is guided by the theory of traditional Chinese medicine, through clinical controlled experimental study, to observe the effect of Fuzheng Yiliu Decoction on inhibiting tumor growth and improving clinical symptoms in patients with non-small cell lung cancer of Qi deficiency and blood stasis type, so as to find out whether the positive effect of Fuzheng Yiliu Decoction in these aspects has statistical significance, and then to make the prescription widely used in non-small cell lung cancer. The clinical treatment of small cell lung cancer provides more detailed theoretical basis.

Method:

Sixty patients with non-small cell lung cancer of Qi deficiency and blood stasis type who met the inclusion criteria were randomly divided into control group (30 cases) and treatment group (30 cases). The control group received DP (docetaxel + cisplatin) regimen chemotherapy for 4 cycles. The treatment group received DP regimen chemotherapy for 4 cycles, while the treatment group received Fu Zheng Yi Tumor Decoction orally for 21 days as a cycle. After 4 cycles of continuous treatment, the changes of serum CEA and CA125 in the treatment group were compared. The changes of condition, quality of life score, TCM syndrome score, safety index (coagulation, blood routine, liver function, kidney function, etc.). SPSS software was used for statistical data processing.

Results:

During the period of treatment, there were 3 cases of abscission, and 57 cases met the criteria were collected.

1. The levels of CEA and CA211 in patients treated with Fuzheng Yiliu Decoction combined with chemotherapy and chemotherapy alone were lower than those before treatment, with statistical significance ($P < 0.05$); and the levels of CEA and CA211 in patients treated with Fuzheng Yiliu Decoction combined with chemotherapy were higher than those in control group, with

中国知网 https://www.cnki.net

statistical significance ($P < 0.05$).

2. The TCM syndromes of patients treated with Fuzheng Yiliu Decoction combined with chemotherapy and chemotherapy alone were improved compared with those before treatment, with statistical significance ($P < 0.05$); and the TCM syndromes relief level of patients treated with Fuzheng Yiliu Decoction combined with chemotherapy was higher than that of the control group, with statistical significance ($P < 0.05$); In KPS score, both the treatment group and the control group improved after treatment, and the treatment group improved. The degree was significantly higher than that of the control group, and the difference was statistically significant ($P < 0.05$).

3. The decrease of leukocyte, platelet and hemoglobin in the treatment group was significantly lighter than that in the control group ($P < 0.05$); the gastrointestinal reaction in the treatment group was significantly better than that in the control group ($P < 0.05$); the liver and kidney function in the treatment group was better than that in the control group ($P < 0.05$). The difference was not statistically significant ($P > 0.05$).

Conclusion:

1. Fuzheng Yiliu Decoction combined with chemotherapy can significantly reduce the levels of serum tumor markers CEA and CA211 in patients with non-small cell lung cancer of Qi deficiency and blood stasis type.

2. Fuzheng Yiliu Decoction can improve the TCM syndromes and quality of life of patients with non-small cell lung cancer of Qi deficiency and blood stasis type undergoing chemotherapy.

3. Fuzheng Yiliu Decoction can improve the decrease of leukocyte, platelet and hemoglobin counts and adverse reactions of digestive tract in patients with non-small cell lung cancer of Qi deficiency and blood stasis type, and has no significant effect on liver and kidney function.

Key words: Non-small cell lung cancer

Qi deficiency and blood stasis

中国知网 https://www.cnki.net

Docetaxel plus cisplatin

Fuzheng Yiliu Decoction

中国知网 https://www.cnki.net

前 言

原发性支气管肺癌简称肺癌，发病率和死亡率很高，是对人的健康和生命威胁最大的常见恶性肿瘤之一。随着人类社会的进步和科技的不断发展，人们的生存环境和生活习惯也受到巨大的影响，与此同时，癌症的发生与生活环境和生活方式密切相关[1]。其中严重的人口老龄化，环境恶化加剧，高吸烟率是肺癌发生的危险因素[2]。仅以中国为例，根据 2017 年全国肿瘤登记中心收集汇总的 2014 年登记资料进行分析整理显示，肺癌的发病率和死亡率位居全国首位，每年发病约 78.1 万例，死亡约 62.6 万例[3]。不仅是在中国，肺癌在许多国家的发病率和死亡率都显著增加，严重威胁着人类的生命健康。因此，有必要对肺癌的特性和治疗方案进行总结，并对其进行更深层次的探讨和研究。

肺癌包括非小细胞肺癌（non-small cell lung cancer, NSCLC）和小细胞肺癌（small cell lung cancer, SCLC）两大种类。NSCLC 主要为鳞癌、腺癌，占全部肺癌的 70%（鳞癌在肺癌中占 30%，腺癌占 40%），其发病隐匿，超过 40%的患者在治疗后易复发，预后较差，5 年生存率不到 15%，如无抗肿瘤治疗，平均生存期仅为 7～15 个月。临床上目前治疗肺癌的最主要方法包括手术治疗、化学药物治疗（化疗）和放射治疗（放疗）。化疗是晚期 NSCLC（ⅢB 期、Ⅳ期）的有效治疗手段，可根据病情需要，结合放疗、介入治疗、靶向治疗等综合治疗。临床上常选择 DP 为主要化疗方案，患者生存期得到明显延长，但化疗带来的毒副反应对患者生存质量产生一定的影响。所以，改善化疗所产生的毒副反应具有重要的现实意义。

运用中西医结合手段治疗恶性肿瘤已经成为一种新的趋势，中医以整体观念和辨证论治为理论基础与西医治疗方法相结合，进一步加强西医的治疗效果，防止肿瘤的复发和转移，减轻西医治疗带来的毒副反应，提高患者的抗病能力，改善患者的生存质量，延长患者生命期限。

本研究采用 DP 方案化疗结合扶正抑瘤汤治疗气虚血瘀型非小细胞肺癌，观察其对患者血清 CEA、CA211 变化情况、中医证候评分、生存质量评分、相关毒副反应及安全指标（血常规、肝功、肾功等）的变化情况。并进一步研究本方剂的有效性，为临床运用提供可靠的实验依据。

中国知网 https://www.cnki.net

文献综述

1.肺癌的现代医学研究进展

1.1 外科手术治疗（surgical treatment）

外科手术作为一种根治性疗法，是肺癌的首选治疗方式，是最有可能治愈肺癌的手段。非小细胞肺癌的手术适应症为临床Ⅰ、Ⅱ期以及可以完全切除的ⅢA 期的患者，为疾病的早期阶段。对于Ⅰ、Ⅱ期患者还可行胸腔镜下切除手术，它具有创伤小、恢复快的特点。对于部分ⅢA 期患者可在手术前通过化疗使肿瘤缩小，提高手术切除治愈率。对于部分ⅢB 期和Ⅳ期孤立性转移病灶（脑、肾上腺及肺内转移等）患者，也可予姑息性手术切除治疗或以手术为主的综合治疗。另外对于通过检查未能确诊的且疑似Ⅰ期或Ⅱ期肺癌的患者，可以考虑进行手术探查帮助确诊及治疗[4]。

1.2 化学治疗（Chemotherapy）

外科手术是肺癌患者首选治疗方法，但多数患者在诊断为肺癌时已经属于中晚期，存在远处转移的情况，错过了手术的时机。这种情况下最合适的治疗手段则为化疗。根据肺癌患者的病理类型选择合适的化疗方案，清除癌细胞，抑制疾病的进一步发展。对于可以实行手术的患者，化疗具有在术前缩小病灶，提高手术疗效的作用。非小细胞肺癌的具体化疗方案如下：

一线化疗：目前标准的一线化疗方案为含铂类的两药方案，第 3 代化疗药物主要为紫杉醇、培美曲塞、吉西他滨等，可分别与铂类药物组合成为两药方案。此方案可有效的缓解疾病，提高患者的生存质量，延长患者的生命期限。但铂类药物具有较强的骨髓抑制、肾毒性等毒副反应。因此也可以将化疗新药与非铂类药物组合使用，如吉西他滨+多西他赛的联合疗效较好、毒性较低[5]。

二线化疗：此种化疗方案可用于经过一线化疗后疾病未见好转且有恶化倾向的患者，主要运用多西他赛、培美曲塞、吉西他滨等化疗新药。ⅠA 期患者，手术后不需要进行化疗；对于多数具有复发转移倾向的ⅠB 期，以及Ⅱ~Ⅲ期 NSCLC 患者，可进行术后化疗。化疗方案以含铂类方案为主，开始时间一般在术后 3~4 周，通常需要完成 2~6 个周期[6]。

中国知网 https://www.cnki.net

1.3 放射治疗（Radiotherapy）

放射治疗作为治疗肺癌的有效局部治疗手段之一，主要包括常规分割/加速超分割放射治疗、三维适形放射治疗、全脑预防性照射治疗等。广泛应用于不能手术的患者，通过放射线对肿瘤病灶进行照射来破坏和消除癌细胞，防止其发生进一步生长、增殖和转移，治疗效果和远期生存情况与外科大致相似。

Crabtree T 等[7]将Ⅰ期非小细胞肺癌患者随机分为手术切除组及放疗组，对两组患者进行观察比较后发现，放疗组的肿瘤复发情况和远期生存情况与手术切除组基本相似，无统计学意义（$P>0.05$）。Palma D[8]等选取平均年龄为 75 岁的早期非小细胞肺癌患者共 9093 例随机分为手术切除组和放疗组，对比两组在生存率方面大致相等。

综上所述，对于肺癌早期拒绝外科手术治疗的患者、发生转移错过手术时机的患者以及年龄较大且心肺功能较差的患者可以进行放射治疗，并有可能达到与外科手术切除相类似的治疗效果。

1.4 靶向治疗（Targeted therapy）

目前分子靶向治疗作为适合个体化的精准治疗已经成为治疗非小细胞肺癌的新兴治疗手段。由于肿瘤组织细胞和正常组织细胞在分子细胞生物学方面具有一定程度的不同，因此可以从基因水平分析能够作为靶点的调控分子、细胞受体等信号传导进行治疗。

现已经批准上市的治疗非小细胞肺癌的分子靶向药物有 5 类，分别为针对表皮生长因子受体（Epidermal growthfactor receptor, EGFR）的酪氨酸激酶抑制剂（TKI）、针对间变性淋巴瘤激酶（Anaplastic lymphoma kinase, ALK）抑制剂、针对人血管内皮生长因子（VEGF）的靶向药物、针对人血管内皮生长因子受体 2（VEGFR 2）的靶向药物、免疫治疗药物。

由于分子靶向治疗不仅为单一靶点治疗还包括多靶点治疗。通过大量实验研究发现，多靶点治疗与单一靶点治疗相比可以在降低对正常组织损伤程度的情况下更精准的作用于肿瘤组织，大大降低了药物的毒副反应，且安全性和耐受性较好。

1.5 其他治疗方法

除了上述几种主要治疗手段，还包括有介入治疗、热疗、消融治疗，

中国知网 https://www.cnki.net

支持疗法等，目前这些治疗方法虽然疗效一般，并且还处在研究当中，但可作为非小细胞肺癌综合治疗的一部分，发挥重要作用。

2.肺癌的中医药学研究进展

2.1 古代中医学对肺癌病名的阐述

祖国医学源远流长、博大精深，流传至今，经过无数中医人的努力和付出，历久弥新，是我国劳动人民智慧的结晶。尤其是在癌症治疗方面，考虑到西医的治疗手段，化疗的适应证范围和毒副作用，中医在癌症治疗上的重要性更加凸显出来。

由于命名的不同，在古代一些典籍中，虽然没有“肺癌”这一说法，但是依据证候特点的差异，同样能够找到诸多类似“肺癌”的记载。《难经•论五脏积病》有云：“肺之积名曰息贲……久不已，令人洒淅寒热，喘咳，发肺痈。”《济生方卷四》论述：“息贲之状，在右胁下，覆大如杯，喘息奔溢，是为肺积；诊其脉浮而毛，其色白，其病气逆，背痛少气，喜忘目瞑……皮中时痛，或如虱啄，或如针刺。”《素问•奇病论》则提到“病胁下满气逆，二三岁不已，是为何病？岐伯曰：病名曰息积……药不能独治也。”依据上文记载所提出的类似肺癌的病名，可以了解到古代医家对肺癌已经有了初步的认识。《素问•咳论》云“肺咳之状，咳而喘息有音，甚则唾血……”描述了咳嗽、咳血的症状；《景岳全书•虚损》曰：“劳嗽、声哑，声不能出或喘息气促者，此肺脏败也，必死。”则为肺癌疾病危急之状。因此可将肺癌归属于“肺积”、“息贲”、“息积”、“咳嗽”、“咳血”等范畴。

2.2 肺癌的病因病机

关于肺癌的病因目前仍未得到明确的阐述。中医学认为肺癌的病因多为正气内虚、饮食及情志等的不调导致肺脏及除肺脏以外的肝、脾、肾、胃等脏腑功能下降，并有气血阴阳失于平衡协调，从而机体抗病能力发生障碍，各种邪气单独或兼夹侵袭机体所致。现代医家对于肺癌病因病机的观点各有其侧重点。

郁仁存[9]教授认为发生肺癌的原因主要在于正气虚损，邪毒结聚于内。病理因素可概括为邪毒袭肺、痰湿内结、正气亏虚三点。其中患病的主要内在原因为脏腑阴阳失调，正气虚损，并有肺、脾、肾三脏功能失调导致

中国知网 https://www.cnki.net

肺气亏虚。吸烟是导致肺癌发生的主要外因之一，长期吸烟则郁热于内，热邪灼伤津液，阴液耗损，致肺阴亏虚，气阴两虚，抗病能力减弱，外邪乘虚而入，邪气留滞于体内，阻滞气机，气滞则血液运行不畅，日久而成肿块。孙桂芝[10]教授认为肺癌致病的原因主要是正气亏虚，肺气不足，邪毒袭肺，致使肺失宣降，逐渐形成肿块。长时间吸烟患者，热盛伤津、耗损阴液，则气阴两虚，肺气宣降失司，外邪侵袭而使气血运行受阻，结聚于身体局部而成肿块。刘嘉湘[11]教授认为，中医从整体观出发来认识肺癌的发病机制，它是一种可发展为全身症状的肺部疾病。因此，发生肺癌的主要原因是由于气血阴阳失于平衡协调，使脏腑功能逐渐退化，机体抵抗外邪侵袭的能力下降，外邪毒气侵袭于肺，邪留胸中，肺气宣降失于平衡，气滞而血液运行受阻，津液失于输布，凝聚成痰，痰瘀互结于胸中，日久形成肺部肿块。刘丽坤[12]认为正气亏虚是肺癌发生、复发和转移的最关键因素，其中正气亏虚的本质在于肺阴不足，贯穿于肺癌疾病的早、中、晚各期，最主要的病理产物则为邪毒内结、痰瘀互阻。周岱翰[13]教授结合中医经典古籍对肿瘤的论述和现代医学对肿瘤的研究进展，将恶性肿瘤的病机总结为虚、瘀、痰、毒四种。李佩文[14]教授则认为，日益严重的环境污染问题是导致肺癌发生率不断上升的主要原因之一，毒邪蕴结于肺内致使肺气宣降失司，气滞血瘀，气血与毒邪相互搏结郁积于体内，日久则发为肺癌。

2.3 肺癌的辨证分型

肺癌是一种表现于局部的全身性疾病，由于患者存在体质、年龄、病因病机、病理类型及临床分期等的不同则表现出不同的临床症状与体征，因此辨证分型也有所差异。关于肺癌的中医辨证分型尚未得到完全统一，各医家依据自身的临床经验总结出了不同的辨证分型。

孙桂芝[15]教授强调辨病与辨证相结合，辨证分型主要为以下 3 种：①肺燥津伤证，本证的病机为肺气郁闭于内，宣降功能失常，兼有痰热和瘀血。同时，燥热伤及肺阴，导致气阴两虚。该证型的临床表现主要为：干咳无痰，痰少而黏，不易咯出，痰中带血，甚则胸痛或胸闷气短，鼻咽口舌干燥，尿少，大便干结，舌质红，少苔，苔色薄黄质干燥，脉细涩。②痰瘀互结，火毒内聚证，本证的病机主要特点为热象、痰毒表现更为突出。

中国知网 https://www.cnki.net

该证型的临床表现主要为：咳嗽，咳黄色黏稠样痰，或痰中有血丝，严重者出现吐血、衄血，或胸痛，情绪急躁易怒，或有心中烦躁不安，发热，面红目赤，口渴而咽干，喜冷饮，睡眠差，尿黄，大便干结，舌质红，苔黄腻而厚，脉数。③肺肾阴虚证，本证的病机为患者素体阴虚，燥热内结，同时兼有痰、瘀、热、毒等邪气。该证型的临床表现主要为：干咳少痰或无痰，痰中有血丝，严重者出现大咯血，低热，午后或夜间潮热，骨蒸潮热，盗汗，心烦失眠，口燥咽干，小便短赤，大便秘结，舌红少苔，脉细数。

郁仁存[16]教授通过多年的临床实践，将非小细胞肺癌的辨证分型总结为以下 4 种：①阴虚毒热证：咳嗽咳痰，痰少而黏腻，或痰中带血丝，心中烦躁，胸中疼痛，少气，或有低热，口燥咽干，声音嘶哑，盗汗，小便短赤，大便干结，舌质红苔黄，脉细数。②痰湿蕴肺证：咳嗽声重，痰多而黏腻，胸中憋闷，纳呆呕恶，神疲乏力，肢体重着，大便溏泻，舌质较暗或淡胖娇嫩，苔白腻，脉滑数。此证型多见于手术后或化疗后的非小细胞肺癌患者。③气滞毒瘀证：咳嗽气急而短促，痰中带血，色暗红，胸部刺痛，口干便干，唇色暗紫，舌质淡青或青紫有瘀点瘀斑，苔黄而薄，脉弦细而涩。此证型多见于非小细胞肺癌的中晚期。④肺肾两虚证：喘咳气急，动则尤甚，痰少，咳出无力，声音嘶哑，胸腹满闷，神疲乏力，腰膝酸软，盗汗，舌淡红少苔，脉沉细无力，尺脉弱。此证型多见于晚期非小细胞肺癌患者。

胡小梅[17]等将非小细胞肺癌总结为以下证型：气虚型、气虚血瘀型、气虚兼气滞型、气虚痰湿型、气虚痰热型、血瘀型、血瘀兼痰湿型、气滞血瘀型、痰湿型、阴虚型、阳虚型、气阴两虚型。其中包括以气虚和血瘀为主的兼证较多，多为虚实夹杂。李本达[18]对 100 例非小细胞肺癌患者化疗前后的临床表现进行分析，观察患者在不同阶段的证候变化情况，将非小细胞肺癌的证型总结如下：气滞血瘀型、气虚血瘀型、血瘀型、气虚痰热型、气虚痰湿型、气阴两虚型。朴炳奎[19]教授将肺癌主要分为以下 5 种类型：气滞血瘀型、痰湿瘀阻型、肺气不足型、阴虚内热型、气阴两虚型。周仲瑛[20]教授总结，肺癌的本虚以阴虚、气阴两虚为主，标实则以气滞、血瘀、痰凝、毒聚为主，为虚实夹杂。王笑民[21-22]教授对 108 例非小细胞

中国知网 https://www.cnki.net

肺癌患者进行分析后发现多数患者以气虚血瘀型为主。

2.4 肺癌的中医治疗

2.4.1 中药汤剂

西医在治疗肺癌时多采用手术、化学治疗、放射治疗、生物治疗、靶向治疗等治疗方法，而中医药根据肺癌患者不同的中医证型应用相应的治疗方法以及中药汤剂。近年来，各医家运用中药汤剂治疗肺癌方面也取得了良好的疗效。

胡艳等[23]将 60 例非小细胞肺癌患者进行随机分组，其中对照组予以常规治疗，治疗组在此基础上口服中药汤剂益气活血方。治疗组在降低患者血液高凝状态方面的作用较对照组强，并且延长了中晚期非小细胞肺癌患者的生存期，提高了患者的生存质量。刘嘉湘等[24]分析 310 例肺癌患者的中医证型后发现最主要的证型为阴虚内热型和气阴两虚型，并经过多年的研究及临床经验研制了益肺抗瘤饮（药物组成主要为黄芪、重楼、北沙参、石上柏、天冬、女贞子等），该中药汤剂具有清热散结，养阴益气等功效，经过实验研究后认为其在改善化疗症状及提高免疫力等方面发挥了积极的作用。孙桂芝[25]教授认为，肺癌中晚期患者多为肺肾阴虚证型，其中阴虚内热型给予百合固金汤合千金苇茎汤加减；气虚痰阻型给予黄芪建中汤加减；气血瘀滞型给予逍遥散合桃红四物汤加减；肺肾两虚型给予麦味地黄丸或参芪地黄汤加减，均能得到良好的疗效。林洪生[26]教授将非小细胞肺癌分为对照组化疗患者 10 例，治疗组 25 例，治疗组予以中药复方肺瘤平Ⅱ号（以白花蛇舌草、三七、黄芪、重楼、西洋参、桃仁等药物为主）治疗，结果显示治疗组患者的 1 年生存率较对照组高 18%。朴炳奎[27]教授认为肺癌晚期患者的中医证型主要为气阴两虚，热毒内蕴，因此以益气养阴、清热解毒为功效的益肺清化颗粒（主要有黄芪、杏仁、党参、桔梗、败酱草、沙参、白花蛇舌草等组成）的使用，减轻了肺癌晚期患者的临床症状、有效的提高了患者的生存质量。周岱翰[28]认为非小细胞肺癌主要包括以下 4 种类型：肺郁痰瘀型、阴虚痰热型、脾虚痰湿型、气阴两虚型，分别拟定不同的中药汤剂。肺郁痰瘀型，药物组成包括：桔梗、苦杏仁、浙贝母、生胆南星、生半夏、薏苡仁、夏枯草、桃仁、仙鹤草、瓜蒌、鱼腥草、田七、壁虎；脾虚痰湿型药物组成包括：党参、白术、茯苓、猪

中国知网 https://www.cnki.net

苓、桔梗、浙贝母、生胆南星、生半夏、薏苡仁、瓜蒌、壁虎；阴虚痰热型药物组成包括：桔梗、浙贝母、生地黄、薏苡仁、夏枯草、仙鹤草、壁虎、猪苓、鳖甲、沙参、麦门冬；气阴两虚型药物组成包括：西洋参、猪苓、桔梗、浙贝母、薏苡仁、瓜蒌、壁虎、麦冬、百合、沙参、仙鹤草、党参、五味子。

2.4.2 中成药制剂

中成药制剂是以中医药理论为指导，在传统中药制剂方法的基础上的不断加工与探索。近年来，越来越多治疗方便，疗效佳的中成药制剂广泛应用于肺癌的治疗中，并获得了良好的效果。

姜福胜等[29]对康莱特注射液进行了实验研究，将非小细胞肺癌患者随机分为对照组和治疗组，治疗组在对照组的基础上给予康莱特注射液（薏苡仁提取物），结果发现治疗组的卡氏评分较对照组高，并且具有一定程度的减毒增效作用。邹波峰等[30]将非小细胞肺癌患者随机分为对照组和治疗组各 30 例，对照组给予西医化学治疗，治疗组在对照组的基础上给予艾迪注射液治疗，结果显示两组在对比病变发展率时具有显著的差异，艾迪注射液在延缓疾病的发展及提高患者的生存质量方面发挥了重要的作用。邵海燕等[31]在进行对比实验后研究发现复方斑蝥胶囊可以改善躯体、心理、社会和认知维度，使毒副反应低于西药，提高患者的免疫力及生存率。田华琴等[32]将非小细胞肺癌患者随机分为对照组和治疗组，对照组予以西药制剂，治疗组在对照组的基础上给予鸦胆子油乳注射液，实验研究结果显示，鸦胆子油具有保护细胞免疫功能、降低西药制剂产生的毒副反应、提高患者生活质量的作用。文淑珍等[33]在对消癌平口服液进行对照实验研究后发现其不仅能缓解患者的临床症状，提高生活质量，并且在防止消化道不良反应发生方面发挥了极大的作用。

2.5 肺癌的中西医结合治疗

现代医学在治疗肺癌方面主要采用手术、化疗、放疗、分子靶向治疗等手段，在控制肿瘤的复发和转移方面发挥了优势，但现代医学在治疗肿瘤时出现的毒副反应对患者的生存质量造成了一定的影响，在各医家长期的实验研究及临床经验中发现中医治疗具有较好的减毒增效作用，因此运用中西医结合手段治疗肺癌已经成为一种趋势。

中国知网 https://www.cnki.net

2.5.1 中药配合手术

中医认为，肺癌患者在手术后会出现气血不足的表现，且抗病力减弱。而中医药在扶助人体正气，补益气血方面具有明显的优势，可促进患者的恢复，改善患者的临床症状，提高机体免疫力。较常用到的中药汤剂包括：人参养荣汤、八珍汤、补中益气汤等。

俞杞泉等[34]选取手术后的非小细胞肺癌患者共142例随机分为对照组和治疗组，对照组给予常规治疗，治疗组在常规治疗的基础上给予中药汤剂治疗，结果显示治疗组在中医证候、疼痛、咳痰量方面改善更加明显，较对照组有效率高，具有统计学意义。朱丽华等[35]通过研究显示非小细胞肺癌术后患者在服用中药治疗后生存期较仅进行常规治疗的患者生存期明显延长，无瘤生存率明显升高，均具有统计学意义。因此，中医药对于肺癌术后患者具有明显的保护作用。术前可使用四君子汤、归脾汤等中药汤剂以补益气血，提高耐受能力；术后可使用八珍汤、补中益气汤等促进患者疾病的恢复，防止复发转移。寿伟臻[36]等研究发现，晚期非小细胞肺癌术后患者在常规治疗的基础上给予金宁方加味辅助治疗，可有效的控制血清肿瘤标志物的数值，改善患者的毒副反应，抑制肿瘤的复发和转移，提高生存质量，延长生存期限。陈慧[37]等在临床中观察到非小细胞肺癌术后患者会出现一定程度的并发症，通过中医辨病与辨证相结合理论研究后认为其证型主要为气血不足证、气阴两虚证、脾胃虚弱证等，因此在术后应注重这三方面的治疗，分别采用补益气血，益气养阴，调理脾胃等治疗原则以防止肿瘤的复发转移。

2.5.2 中药配合化疗

化疗是治疗肺癌最主要的手段，具有规范的治疗方案，可在术前或者术后进行化疗，有效的抑制了肿瘤细胞的生长，防止其术后复发转移。但化疗在杀死癌细胞的同时也产生了恶心、呕吐、食欲减退、脱发、骨髓抑制、肝肾毒性等一系列毒副反应，造成了患者的不适感，严重影响了患者的生存质量。由于中药在减轻化疗毒副反应方面具有明显的优势，可有效的减轻患者的临床症状，因此中药配合化疗的治疗手段起到了一定的减毒增效作用。侯恩存[38]等将晚期 NSCLC 患者随机分为对照组 31 例，采取单纯 NP 方案化疗，治疗组 33 例，采取中西医结合治疗。根据辨证分型的不

中国知网 https://www.cnki.net

同使用相应的中药方剂，实验结果显示两组在对比患者的体重变化、中医证候评分方面发现两组具有显著差异，尤其是治疗组可以明显的减轻化疗的毒副反应，提高患者的生存质量。

李道阳[39]将非小细胞肺癌患者分为单纯化疗组 61 例，中药自拟扶正抗癌方联合化疗组 63 例，药物组成主要为黄芪、人参、白花蛇舌草、半枝莲、薏苡仁、仙鹤草、黄精、大黄、猪苓等。两组对比观察后发现中药联合化疗组有效的降低了化疗的毒副反应，并且完全缓解或部分缓解的比率较单纯化疗组高 14%。何邦志[40]等对肺癌胸腔积液患者分为对照组和治疗组，对照组进行抽胸水及胸腔内注射化疗药物，治疗组采取化疗的同时联合苓桂术甘汤加味治疗，结果显示治疗组的总有效率明显高于对照组。中药联合化疗在消退胸腔积液、缓解不适症状方面具有更好的治疗效果。周岱翰[41]将非小细胞肺癌患者随机分为三组，中医组通过辨证后按照不同的证型分别给予鹤蟾片、参一胶囊口服治疗；西医组采用 NP 或 VP 方案化疗；中西医结合组则在西医化疗组的基础上口服中药治疗。90 天后三组对比观察发现，中西医结合组肿瘤大小变化率最大、稳定率最高，并且在解决咳嗽、食欲差、恶心、呕吐等化疗毒副反应方面具有明显的优势。刘莉等[42]将老年晚期非小细胞肺癌患者 120 例随机分为对照组和治疗组，对照组采用的化疗方案为多西紫杉醇+奥沙利铂，治疗组给予中药汤剂口服和中成药制剂艾迪注射液联合治疗，中药汤剂以自制扶正解毒汤剂加味，药物组成以黄芪、白术、鸡血藤、女贞子、白花蛇舌草等为主，并随不同的症状进行加减。连续治疗 6 个月后显示治疗组在控制患者病情方面占有更大的优势。寿伟臻等[36]将非小细胞肺癌患者进行对照组和化疗组对比治疗，对照组予以 TP 方案单纯化疗，治疗组给予金宁芳加味联合 TP 方案化疗。研究结果显示中药联合化疗可有效降低血清肿瘤标志物 CA-125 数值，1 年内复发转移率明显低于对照组。

2.5.3 中药配合放疗

肺癌患者在进行放疗后出现以肺燥津伤、阴虚内热为主的一系列症状。进过多年的研究发现，中药联合放疗可有效的缓解放疗后产生的热毒对组织的损伤，具有清热生津，益气养阴的重要作用。

孙太振等[43]将 48 例肺癌放疗后出现放射性肺炎的患者随机分为对照

中国知网 https://www.cnki.net

组和治疗组，对照组给予西药抗生素、糖皮质激素、维生素等常规治疗，中医将放射性肺炎分为三期，每一期的证型各不相同，初期表现为气滞血瘀，肺燥伤阴为主，治疗给予行气活血，润肺养阴中药；中期表现为热毒伤肺，痰浊阻肺为主，治以清热解毒、润肺化痰；后期则多表现出虚证，以气血阴液亏虚为主，治以益气补血养阴为主。研究结果表明在有效率方面治疗组高于对照组，有统计学意义，在进展率方面，治疗组明显低于对照组，有显著的统计学意义。蔡凯等[44]发现晚期非小细胞肺癌患者放疗后主要表现为咽干、咽痛、口干等一系列气阴两虚的临床症状，经过分组实验研究后发现益气养阴中药汤剂可有效的降低放射性肺炎及食管炎的发生率，提高机体抗病能力，改善患者的生存质量。邓生明等[45]采用单纯放疗治疗肺癌，并与天龙消瘤方联合放疗进行对比研究，结果显示中药联合放疗组有效率及生存率均高于对照组，治疗效果更佳。

2.5.4 中药配合分子靶向治疗

分子靶向治疗作为治疗恶性肿瘤的新手段比较于放、化疗具有更好的治疗效果，可根据基因检测结果应用与靶点相对应的药物进行治疗。同放、化疗一样，分子靶向治疗也有一定的副作用，比如皮疹、腹泻等异常的体征及症状。

高启龙等[46]将晚期非小细胞肺癌患者分为单纯化疗及分子靶向治疗为对照组和在对照组的基础上联合中药益气散结方为治疗组，对比研究后发现，治疗组在稳定病情、延长患者生存期方面明显优于对照组（$P<0.05$）。

郭鹏等[47]认为肺癌患者在使用靶向药物特罗凯后引起的皮疹属于中医的“药毒”，根据皮疹的特征、患者自身的体质、症状、舌脉等可分为血虚、血热、湿热、风热 4 种不同的辨证分型，在治疗时则相应的给予补血、凉血、清热化湿、疏风清热药物。通过研究后发现中药在治疗放疗后皮疹方面效果明显。

中国知网 https://www.cnki.net

临床观察

1. 研究资料

1.1 病例来源

本临床研究收集 2018 年 01 月-2018 年 10 月于黑龙江中医药大学附属第一医院肿瘤科病房诊断和治疗且符合标准的中晚期非小细胞肺癌患者 60 例，随机分为对照组和治疗组各 30 例，对照组采用常规单纯化疗，治疗组在常规化疗的基础上予以口服中药汤剂治疗。

1.2 诊断标准

1.2.1 西医诊断标准

参照我国卫生健康委员会颁布的《原发性肺癌诊疗规范》（2018 年版）肺癌制定标准。

1.2.1.1 临床表现

（1）症状及体征：①原发肿瘤本身局部生长引起的症状，包括咳嗽、咯血、呼吸困难、发热、喘鸣等。②原发肿瘤侵犯邻近器官、结构引起的症状，包括胸腔积液，声音嘶哑，吞咽困难，心包积液，上腔静脉综合征等。③肿瘤远处转移引起的症状，最常见的为头痛、恶心、呕吐等中枢神经系统症状；骨转移则出现剧烈而持久且进行性加重的疼痛症状。④肺癌的肺外表现，常见于异位内分泌、骨关节代谢异常、神经肌肉传导障碍等。

（2）血清肿瘤标志物检测：目前认为 CEA、NSE、Cyfra21-1、ProGRP、SCC 等肿瘤标志物的联合使用，可提高肺癌诊断的敏感度和特异度。其中 NSCLC 的诊断主要依靠 CEA、Cyfra21-1 和 SCC 水平的升高。

（3）影像学检查：①X 线：对早期肺癌的诊断起辅助作用，结果显示疑似肺癌时应及时行 CT 检查。②胸部 CT：是肺癌早期诊断、判断临床分期及观察疗效的重要手段。③MRI：常用于判断胸壁、纵隔、肺门大血管的受侵及淋巴结肿大情况；可判定肿瘤复发及脑、脊髓转移情况。⑤骨核素扫描：主要用于判断骨转移情况，可通过 CT、MRI 等检查进行验证。⑥超声：主要用于锁骨上区淋巴结及肾脏、肝脏等脏器转移情况的观察，对判定肿瘤分期情况起辅助作用。

1.2.1.2 病理组织学诊断

通过对组织标本进行活检后进行病理检查判断是否存在肿瘤及肿瘤类

中国知网 https://www.cnki.net

型，病理诊断的亚型分类适用于肺癌晚期不能手术的患者，可结合免疫组化染色应用于诊断形态学不典型的病例。对于非特殊类型诊断的使用应尽量避免。

1.2.1.3 其他诊断

除上述诊断方法以外还可通过支气管镜、纵隔镜、胸腔镜检查，超声支气管穿刺活检术和开胸肺活检术及痰脱落细胞学检查对肺癌进行诊断。

1.2.1.4 分期标准

参照国际肺癌研究协会 2017 年第 8 版 TNM 分期标准（见附表 1）。

1.2.2 中医诊断标准

肺癌气虚血瘀证中医诊断标准，参照国家药品监督管理局 2002 年《中药新药临床研究指导原则》：

主症：咳嗽，咯痰，痰中带血，胸痛，气喘。

次症：神疲乏力，气短懒言，形体消瘦，纳呆食少，面色晦暗，心悸，自汗。

舌脉象：舌黯淡或紫暗，舌体胖大，边有齿痕，苔少，薄白，脉细涩。

1.3.纳入标准

①符合非小细胞肺癌西医诊断标准，临床分期为Ⅲb、Ⅳ期，年龄在 18-75 岁之间者；

②符合肺癌气虚血瘀型中医诊断标准；

③肿瘤标志物 CEA、CA211 水平高于正常值；

④预计生存期≥10 个月；

⑤KPS 评分≥60 分；

⑥各脏器功能未见明显异常，且临床指标符合化疗要求者；

⑦入组前两个月内未接受放、化疗及口服中药治疗者；

⑧自愿入组治疗，有较好依从性者；

⑨本临床研究已通过伦理委员会审查批准。

1.4.排除标准

①不符合入组标准者；

②有严重的肝、肾、造血系统、心脑血管等合并症及有精神异常者；

③有严重感染性疾病，尚未得到良好控制；或慢性化脓性炎症，且伤

中国知网 https://www.cnki.net

口未愈合者；

④孕期、哺乳期妇女和儿童；

⑤合并其他部位恶性肿瘤且未治愈者；

⑥对化疗药物或中药有过敏反应及对口服中药适应性较差者。

1.5 脱落、剔除标准

①实验过程中对化疗药物或中药发生严重过敏反应或不能耐受者；

②出现严重并发症或合并症，对实验的有效性造成影响者；

③未按规定服药或自行口服其他药物，影响疗效判断者；

④实验过程中疾病突然恶化死亡者；

⑤因其他各种原因不能完成全部治疗过程，退出实验者；

符合以上任何一条者，属脱落、剔除标准，如实记录原因。因无效而自行脱落者应计入疗效分析;发生不良反应者应记入不良反应统计;其它各种原因脱落、剔除病例不计入统计学分析。

2.研究对象及方法

2.1 研究对象

本次研究选取 60 例符合上述标准的非小细胞肺癌患者,按随机数字表法随机分为治疗组和对照组均为 30 例。在试验结束时，除去脱落病例，则列入最终统计分析的病例治疗组和对照组分别为29例和28例。使用 SPSS25.0 软件对两组患者的性别、年龄、病理类型及临床分期进行统计学处理，各组一般资料比较见表 1~表4:

2.1.1 两组患者性别比较情况

表 1　两组患者性别比较（例）

组别	男患者	女患者	合计例数
治疗组	18	11	29
对照组	15	13	28

由上表分析可以得出，两组患者的性别情况符合卡方检验，χ^2=0.422，p=0.516＞0.05，无显著差异，不具有统计学意义，具有可比性。

中国知网　https://www.cnki.net

2.1.2 两组患者年龄比较情况

表 2　两组患者年龄比较（例）

组别	18-35（岁）	36-55（岁）	56-75（岁）	合计例数
治疗组	4	10	15	29
对照组	3	13	12	28

由上表分析可以得出，两组患者年龄分化情况经卡方检验，χ^2=0.859,p=0.654＞0.05，不具有统计学意义，具有可比性。

2.1.3 两组患者病理类型比较情况

表 3　两组患者病理类型比较（例）

组别	腺癌	鳞癌	合计例数
治疗组	15	14	29
对照组	17	11	28

上表两组数据经过卡方检验χ^2=0.468,p=0.494＞0.05，不具有统计学意义，具有可比性。

2.1.4 两组患者临床分期比较情况

表 4　两组患者临床分期比较（例）

组别	Ⅲb 期	Ⅳ期	合计例数
治疗组	17	12	29
对照组	20	8	28

上表两组数据经过卡方检验χ^2=1.026,p=0.311＞0.05，不具有统计学意义，具有可比性。

2.2 研究方法

对照组（DP 方案化疗）：30 例。给予 DP 方案（多西他赛+顺铂）化疗。

具体方案：多西他赛 75mg/m^2 (扬子江药业集团有限公司，国药准字 H20178011），溶于 5%葡萄糖注射液 250ml 稀释，静脉滴注，第 1 天给药；顺铂 75mg/m^2（齐鲁制药有限公司，国药准字 H20143124），溶于 0.9%氯化钠注射液 250ml 稀释，静脉滴注，第 1 天给药；用药前 1 天开始口服地塞米松 8mg/d（广东华南药业集团有限公司，国药准字 H13020038），分 2

中国知网　https://www.cnki.net

次口服，连用 3 天；21 天为 1 个化疗周期，共化疗 4 周期。

治疗组（DP 方案化疗+扶正抑瘤汤）：30 例，化疗方案同对照组，同时口服扶正抑瘤汤。

组成：黄芪 40g、太子参 15g、白术（炒）15g、茯苓 15g、桔梗 15g、苦杏仁 15g、浙贝母 20g、山慈菇 15g、白花蛇舌草 30g、半枝莲 30g、薏苡仁（生）30g、当归 15g、地龙 15g、鸡血藤 25g、焦山楂 20g、鸡内金 20g、香附 10g、甘草 5g 等组成。

煎服方法：中药由黑龙江中医药大学附属第一医院抓取，每日 1 剂，水煎 300ml 服，早晚 2 次空腹温服，21 天为 1 周期，连续治疗 4 周期。

3.观察指标

3.1 疗效性指标

观察方法：分别在化疗前及化疗联合口服中药 4 周期后进行疗效评价，实验完成后比较各组患者的血清肿瘤标志物 CEA、CA211、中医证候积分、KPS 评分、安全性指标（血常规、肝功、肾功）及临床毒副反应等变化情况。

3.2 疗效评价标准

3.2.1 中医证候（见附表 2）

参照《中药新药临床研究指导原则》患者临床表现的评分标准：无症状计 0 分、轻度计 1 分、中度计 2 分、重度计 3 分，计算公式（莫尼地平法）：积分比例=[(治疗前积分-治疗后积分)/治疗前积分]×100%。比较各组患者治疗前后的积分差值来进行疗效评价，疗效判定标准划分为以下 3 种：

显效（显著改善）：积分在治疗后较治疗前减少≥70%

有效（部分改善）：积分在治疗后较治疗前减少≥30%

无效（未见明显改变）：积分在治疗后较治疗前减少<30%

症状改善包括显著改善和部分改善。

3.2.2 KPS 评分（见附表 3）

参照卡氏（Karnofsky）评分标准进行评定：治疗后较治疗前增加≥10 分以上者为提高；治疗后较治疗前增加或减少＜10 分者为稳定；治疗后较治疗前减少≥10 分以上者为降低。

中国知网 https://www.cnki.net

3.2.3 安全性指标（见附表 4）

观察治疗前后两组患者不良反应情况，包括：血常规（白细胞、血小板、血红蛋白）、肝肾功能（谷丙转氨酶、肌酐）、消化道不良反应（恶心、呕吐、腹泻）。不良反应分度参照 WHO 抗肿瘤药急性及亚急性毒性反应分度标准进行判定，分为 0 度、Ⅰ度、Ⅱ度、Ⅲ度及Ⅳ度共 5 级。

4.统计学方法

统计学处理采用 SPSS25.0 统计软件分析，计量资料采用均数±标准差（$\bar{\chi}$±s）进行描述；计数资料采用频数及百分比进行描述；服从正态分布的分数据，两组间均值的比较采用 t 检验；不服从正态分布分数据，采用秩和检验；两组计数资料构成比之间的比较采用χ^2检验，检验水准 α=0.05，所有结果均以 P<0.05 为标准，认为其差别有统计学意义。

中国知网 https://www.cnki.net

结　果

本临床观察共有60例符合纳入标准的患者，实验过程中共脱落3例，其中治疗组1例，对照组2例，未脱落、剔除的患者口服相应的药物进行治疗，并记录数据。治疗后对两组患者的血清CEA、CA211、临床主要症状的改善情况、KPS评分、安全性指标及毒副反应等情况进行观察记录，整体上依从性较好。

1.两组病例治疗前后血清CEA、CA211的比较结果

表5　治疗前后两组患者CEA情况

组别	时间	CEA
治疗组	治疗前	17.51±3.02
	治疗后	8.66±2.00
对照组	治疗前	18.07±2.42
	治疗后	11.46±1.77

根据治疗前后两组患者CEA情况，将治疗组与对照组进行统计学分析，采用独立样本t检验，治疗组和对照组疗前比较，得出t=0.768，p=0.444>0.05，说明不具有统计学意义；治疗组和对照组治疗前后分别采用配对t检验，p=0.000，p=0.000均小于0.05，疗效确切。治疗后两组比较，p=0.000<0.05，有统计学意义，说明经过治疗后，治疗组比对照组的疗效更明显。

表6　治疗前后两组患者CA211情况

组别	时间	CA211
治疗组	治疗前	10.00±1.60
	治疗后	5.90±1.46
对照组	治疗前	10.80±1.61
	治疗后	7.49±1.17

根据治疗前后两组患者CA211情况，将治疗组与对照组进行统计学分析，采用独立样本t检验，治疗组和对照组疗前比较，得出t=0.768，p=0.444>0.05，说明不具有统计学意义；治疗组和对照组治疗前后分别采用配对t检验，p=0.000，p=0.000均小于0.05，疗效确切。治疗后两组比较p=0.000<0.05，有统计学意义，说明经过治疗后，治疗组比对照组的疗

中国知网　https://www.cnki.net

效更明显。

2.两组病例治疗前后中医证候积分疗效比较

表 7　治疗前后两组患者中医症状疗效比较

组别	例数	显效	有效	无效	总有效率	
治疗组	29	10 (34.48%)	15 (51.72%)	4 （13.79%）	86.20%	Z=1.952 P=0.001
对照组	28	4 (13.79%)	14 （50.00%）	10 (35.71%)	64.29%	

由上表可知，经秩和检验 $p<0.05$，治疗组与对照组疗效构成比有显著差异。

表 8　治疗前后两组患者中医症状评分

组别	症状	治疗前	治疗后	疗前总积分	疗后总积分
治疗组	咳嗽	2.45±0.51	1.00±0.76	20.34±1.42	8.90±3.02
	咳痰	2.55±0.51	1.03±0.63		
	咯血	2.59±0.50	1.10±0.67		
	气短	2.48±0.51	0.97±0.68		
	乏力	2.45±0.51	1.17±0.76		
	胸痛	2.59±0.50	1.24±0.69		
	食欲不振	2.69±0.47	1.17±0.71		
	便秘	2.55±0.51	1.21±0.73		
对照组	咳嗽	2.50±0.51	1.82±0.61	20.57±1.35	11.89±3.53
	咳痰	2.54±0.51	1.39±0.88		
	咯血	2.50±0.51	1.57±0.63		
	气短	2.71±0.46	1.61±0.74		
	乏力	2.64±0.49	1.39±0.69		
	胸痛	2.50±0.51	1.21±0.57		
	食欲不振	2.57±0.50	1.36±0.78		
	便秘	2.61±0.50	1.54±0.84		

中国知网 https://www.cnki.net

进一步由上表治疗组、对照组疗前总积分采用独立样本 t 检验，t=-0.618,p=0.539>0.05，疗前两组中医证候积分无显著差异。治疗组疗前疗后采用配对样本 t 检验，p=0.000＜0.05,对照组疗前疗后采用配对样本 t 检验，p=0.000＜0.05，治疗组和对照组疗效确切。治疗组、对照组疗后总积分采用独立样本 t 检验，得出 t=3.449，p=0.001＜0.05，具有统计学意义，治疗组中医证候疗效优于对照组。

3.两组病例治疗前后 KPS 评分比较

表 9　治疗前后两组患者 KPS 评分情况

组别	时间	KPS 评分
治疗组	治疗前	72.76±7.51
	治疗后	83.45±4.83
对照组	治疗前	71.03±8.17
	治疗后	77.24±8.82

根据治疗前后两组患者 KPS 评分情况，将治疗组与对照组进行统计学分析，采用独立样本 t 检验，治疗组和对照组疗前比较，得出 t=0.426，p=0.803>0.05，说明不具有统计学意义；治疗组和对照组治疗前后分别采用配对 t 检验，p=0.000，p=0.000 均小于 0.05，疗效确切。治疗后两组比较 p=0.001<0.05，有统计学意义，说明经过治疗后，治疗组比对照组的疗效更明显。

中国知网 https://www.cnki.net

4.两组病例治疗前后不良反应比较

表 10　两组不良反应统计

		不良反应						
		白细胞下降	血小板下降	血红蛋白下降	谷丙转氨酶	肌酐	恶心、呕吐	腹泻
对照组	0 度	7	8	7	15	15	8	12
治疗组		20	20	20	22	23	23	23
对照组	Ⅰ度	12	10	11	7	6	12	9
治疗组		6	5	5	5	4	4	4
对照组	Ⅱ度	6	7	7	4	5	6	5
治疗组		2	2	2	1	1	1	1
对照组	Ⅲ度	3	3	2	2	2	2	2
治疗组		1	2	2	1	1	1	1
对照组	Ⅳ度	0	0	1	0	0	0	0
治疗组		0	0	0	0	0	0	0

与对照组白细胞下降相比，经 χ^2 检验，$\chi^2=11.245$，$p=0.01<0.05$

与对照组血小板下降相比，经 χ^2 检验，$\chi^2=9.773$，$p=0.021<0.05$

与对照组血红蛋白下降相比，经 χ^2 检验，$\chi^2=12.273$，$p=0.015<0.05$

与对照组恶心、呕吐情况相比，经 χ^2 检验，$\chi^2=15.150$，$p=0.002<0.05$

与对照组腹泻情况相比，经 χ^2 检验，$\chi^2=8.365$，$p=0.039<0.05$

中国知网　https://www.cnki.net

讨 论

1.立法依据

中国古代医学文献中虽然对“肺癌”这一病名没有确切的记载，但可根据发病特点及病因病机总结出与肺癌相似的病名，可将其归属于“肺积”“息贲”“肺花疮”“咳嗽”“胸痛”“喘证”“虚劳”等范畴。肺积与息贲症状多相类似，如《难经·五十六难》指出：“肺之积，名曰息贲，在右胁下，覆大如杯。久不已，令人洒淅寒热，喘咳，发肺壅。”“肺花疮”在症状及预后差等表现上与肺癌均有许多相似之处，清代的《青囊秘决》论述：“人有久咳之后，肺管损伤，皮肤黄瘦，咽喉雌哑……人以为肺中痈也，谁知是肺痿而生疮乎？此等之症，不易解救。”“咳嗽”“喘证”“胸痛”三个病名是对肺癌的临床症状的描述，其中咳嗽是肺癌最主要也是最明显的症状，多表现为持续不解的干咳和刺激性呛咳。喘证及胸痛则多为肺癌并发症。《素问·玉机真藏论篇》曰：“大骨枯槁，大肉陷下……肉痛引肩项。”描述了疾病末期极度虚劳之象，与肺癌晚期患者出现的各种恶病质大致相似。

中医古籍中对于肺癌病因病机的记载不尽相同，其中《医宗必读》论述：“积之成也，正气不足，而后邪气踞之”。《外证医案》曰：“正气虚则成岩”。故可言之，肺癌的发生主要是由于素体正气亏虚，六淫邪气趁虚而入，饮食不洁、情志不畅，而阻滞气机，气为血之帅，气行则血行，气机不畅则血液运行受阻，而成血瘀，瘀毒交结而成肿块，发为肺癌，成为肺癌发生的最根本病机。

非小细胞肺癌的证型种类较多，尚未完全统一。本研究旨在研究非小细胞肺癌的气虚血瘀型，采取益气活血的治疗原则，将临床经验方剂扶正抑瘤汤联合化疗与单纯化疗的患者进行对比来证实中药汤剂在治疗非小细胞肺癌和减轻化疗毒副反应方面具有明显的优势。

2.扶正抑瘤汤对气虚血瘀型非小细胞肺癌作用机制的探讨

2.1 方药组成

该方药的主要功用为益气补虚、活血化瘀，为防止出现患者出血的现象应注意活血药物的用量，重在补虚。药物组成包括：黄芪、太子参、白术（炒）、茯苓、桔梗、苦杏仁、浙贝母、山慈菇、白花蛇舌草、半枝莲、

中国知网 https://www.cnki.net

薏苡仁（生）、当归、地龙、鸡血藤、焦山楂、鸡内金、香附、甘草。

2.2 组方特点

黄芪味甘，性微温，归脾肺经，功效以补气升阳，益卫固表为主，用于肺气虚弱、咳喘气短等；太子参味甘微苦，性平，归脾肺经，功效为补气生津，用于气虚肺燥之咳嗽；白术味苦甘，性温，归脾胃经，功效以补气健脾，燥湿利水为主，燥湿利水宜生用，补气健脾宜炒用，本方则应用炒白术，用于脾气虚弱，食少神疲；茯苓味甘淡，性平，归心脾肾经，功效为利水渗湿，健脾安神，常与白术同用以健脾补中，治疗脾胃虚弱、神疲乏力。上述四味药共同使用旨在补肺脾之气，补益身之元气。

桔梗味苦辛，性平，归肺经，功效为宣肺祛痰，利咽，用于肺气不宣的咳嗽痰多，胸闷不畅；苦杏仁味苦，性微温，归肺、大肠经，功效为止咳平喘，润肠通便，降肺气之中兼有宣肺之功而用于止咳平喘，为治咳喘之要药；浙贝母味苦，性寒，归肺心经，功效为清热化痰，开郁散结，用于痰热郁肺之咳嗽；山慈菇味甘微辛，性寒，归肝胃经，功效为清热解毒、消痈散结，可广泛应用于消散徵瘕痞块和抗肿瘤。上述四味药共同使用旨在化痰止咳平喘，清热散结，抗肿瘤。

白花蛇舌草味微苦甘，性寒，归胃、大肠、小肠经，功效为清热解毒，利湿通淋；半枝莲味辛苦，性寒，归肺肝肾经，功效为清热解毒，化瘀利尿；薏苡仁（生）味甘淡，性微寒，归脾胃肺经，功效为利水渗湿，健脾，除痹，清热排脓。上述三味药同用具有很强的抑制肿瘤生长、增殖及转移的作用。

当归味甘辛，性温，归肝心脾经，功效为补血，活血，调经，止痛，润肠，具有活血通络而不伤血的特性；地龙味咸，性寒，归肝脾膀胱经，功效为清热熄风，通络，平喘，利尿，用于气虚血滞，长于通行经络；鸡血藤味苦甘，性温，归肝经，功效为行血补血，调经，舒筋活络，用于血虚血瘀之证。上述三位药均为活血之品，共同发挥活血化瘀而不伤正的作用特点。

山楂味酸甘，性微温，归脾胃肝经，功效为消食化积，行气散瘀，本方剂应用的是焦山楂，用于止泻止痢；鸡内金味甘，性平，归脾、胃、小肠、膀胱经，功效为消食健胃，涩精止遗，用于食滞症。上述两味药共同

中国知网 https://www.cnki.net

用于肺癌患者主要在于具有较强的健运脾胃的功能，对于久病、经过放化疗等食欲较差的患者效果尤为明显。香附味辛、微苦、微甘，性平，归肝、脾、三焦经，功效为疏肝理气，调经止痛，为疏肝解郁，行气止痛之要药。甘草味甘，性平，归心肺脾胃经，具有调和药性、益气补中的功效。

2.3 现代药理研究

黄芪：药用黄芪为黄芪和内蒙黄芪的干燥根，主要成分为黄芪多糖、皂苷类及黄酮类等。除此以外，还包括多种氨基酸、少量的微量元素及叶酸等。现代药理研究发现黄芪可增强细胞的免疫功能，促进抗体的生成；促进细胞凋亡，可抑制肿瘤细胞的生长、增殖等，使其分化进而凋亡；提高机体清除可产生肿瘤细胞并促使肿瘤细胞代谢活跃的自由基的能力；抑制肿瘤新生血管的生成，防止肿瘤的复发和转移；减轻化疗后出现的各种不良反应，使生存质量得到保证和提高。另外，黄芪中的微量元素硒可促进肿瘤细胞的分化，进而诱导癌细胞凋亡。

太子参：石竹科植物孩儿参的干燥块根，主要成分为环肽类、皂苷类、氨基酸类、挥发油类、多糖类等。太子参可通过对抗环磷酰胺改善 T、B 淋巴细胞转化功能、白细胞吞噬功能和增加外周血白细胞计数来增强机体的免疫功能；其提取物具有抗氧化性，清除机体的自由基以发挥抗肿瘤的作用；太子参含有的肌醇-甲醚有较强的镇咳作用，可缓解肺癌的咳嗽症状。

白术：菊科植物白术的干燥根茎，主要成分为挥发油、内酯类化合物、多糖等。具有抑制肿瘤细胞增殖的作用；能刺激免疫细胞，使脾脏淋巴细胞增殖，进而有效促进人体外周血淋巴细胞增殖，提高人体的免疫调节作用；可增强人体消化系统功能，调节胃肠运动；保护肝肾功能；并具有一定的抗炎镇痛作用。

茯苓：多孔菌科真菌茯苓的干燥菌核，主要成分为茯苓多糖、茯苓素、蛋白质和脂肪酸等，其中茯苓多糖具有较强的药理作用，并得到广泛应用。大量的动物实验研究表明茯苓可通过激活对抗肿瘤的免疫系统、抑制肿瘤细胞 DNA、RNA 的合成、增加肿瘤细胞膜上的唾液（SA）含量、增强肝脏 SOD 活性而清除氧自由基四个途径来增强人体的免疫功能来实现抑制肿瘤生长及增殖的作用；同时既能增强人体的细胞免疫又可增强体液免疫，改善骨髓抑制情况和减轻化疗引起的毒副反应，如茯苓具有一定的保肝降

中国知网 https://www.cnki.net

酶的作用，对于肿瘤患者化疗后出现的肝功能损害具有良好的修复及保护作用。

桔梗：桔梗科植物桔梗的干燥根，主要成分为三萜皂苷类、黄酮类、酚酸类、脂肪酸以及微量元素。其中桔梗皂苷类具有较强的抗肿瘤作用，主要表现在对肿瘤细胞的增殖、迁移、黏附、侵袭及病理性血管生成的抑制作用，以促进肿瘤细胞的凋亡和自噬；还可通过增强免疫调节来达到抗肿瘤的作用；除此之外，桔梗还具有抗氧化、抗炎、抗肺损伤、止咳平喘和保肝降酶等作用。

苦杏仁：蔷薇科植物杏的干燥成熟种子，其成分主要为苦杏仁苷、苦杏仁酶、苦杏仁苷酶、多种维生素及矿物质元素等。大量动物实验研究表明苦杏仁苷可杀死癌细胞，发挥抗肿瘤的作用，尤其对于不能手术的肺癌患者具有缓解作用；也可以通过抗氧化性清除自由基，增强巨噬细胞的吞噬功能来调节免疫达到抗肿瘤的作用；除此之外，苦杏仁还具有止咳平喘、抗炎镇痛的作用，并对消化系统、循环系统及泌尿系统的部分疾病有一定的缓解作用。

浙贝母：百合科植物浙贝母的干燥鳞茎，主要成分为多种生物碱、浙贝甲素、浙贝乙素等。在呼吸系统方面，可通过松弛支气管平滑肌来镇咳、并可祛痰，对消除炎症作用明显；在消化系统方面，具有消炎止痛及抑制幽门螺杆菌等作用；还具有较好的抗肿瘤和逆转癌细胞耐药的作用。

山慈菇：兰科植物杜鹃花、独蒜兰和云南独蒜兰的干燥假鳞茎，其中主流品种是杜鹃花，其所含成分较多，以菲类化合物、联苄类化合物、简单芳香化合物及其苷类、糖及糖苷类化合物、萜类及甾体类化合物为主。可以直接清除癌细胞，对肿瘤细胞的增殖、侵袭、转移及新生血管的生成具有抑制作用，以诱导细胞凋亡，提高机体免疫力，从而起到抗肿瘤的作用。通过实验研究证明山慈菇还具有增强骨髓造血功能的作用，对于肿瘤患者化疗后产生的骨髓抑制具有治疗作用。

白花蛇舌草：茜草科耳草属植物白花蛇舌草的干燥全草，主要成分为蒽醌类、环烯醚萜苷类、黄酮类、挥发油类、微量元素、多糖类化合物等。抗肿瘤是其最主要的作用，体现在预防肿瘤发生、防止肿瘤的复发和转移等多方面；通过激活巨噬细胞、T 细胞等生物活性以提高机体免疫功能；

中国知网 https://www.cnki.net

同时还具有抗菌消炎和抗氧化的作用。

半枝莲：为唇形科黄芩属植物的干燥全草，以黄酮类、多糖、萜类、挥发油等为主要成分，具有提高机体免疫力，抑制肿瘤新生血管的生成、抗焦油致突变作用，以预防癌症的发生、防止癌细胞复发和转移；对呼吸系统、泌尿系统、消化系统等疾病具有一定的治疗作用。

薏苡仁：禾本科植物薏苡的干燥成熟种仁，主要成分为淀粉类、蛋白质类、脂肪酸及酯类、多酚类等，薏苡仁具有较好的调节免疫的功能，现今广泛应用于抗肿瘤的康莱特注射液主要成分为薏苡仁的提取物薏苡仁油，且对肺癌的作用尤其明显，除此之外还具有抗炎抗菌和止泻等作用。

当归：伞形科植物当归的干燥根，主要成分包括挥发油、多糖、氨基酸、有机酸和黄酮等。当归多糖能刺激血红蛋白、红细胞及造血干细胞的生成，使细胞快速分化，促进造血功能；当归内酯具有增强巨噬细胞的吞噬能力和淋巴细胞的转化能力的作用，从而提高机体的免疫力；除此之外，还具有保肝护肾、抗肿瘤，改善肺损伤等功能。

地龙：钜蚓科动物威廉环毛蚓、栉盲环毛蚓、通俗环毛蚓、参环毛蚓的干燥全体，主要成分为氨基酸类化合物、核苷类化合物、二肽类化合物、有机酸类化合物、无机元素、蛋白质类化合物等。可通过增强机体免疫力，提高巨噬细胞吞噬能力，拮抗化疗和放疗的免疫抑制等来抑制肿瘤生长；可通过抑制血小板聚集来抗凝血、抗血栓的形成；同时具有平喘、解热镇痛的作用。

鸡血藤：豆科植物密花豆的干燥藤茎，主要成分为黄酮类、酚酸类、甾醇类、三萜类、蒽醌类及微量元素等。具有一定的增加血细胞、升高血红蛋白等调节血液系统的作用；在抗肿瘤方面主要分为体内和体外两方面，体内主要为提高免疫、对抗肿瘤生长和减毒增效，体外可促进癌细胞凋亡和自噬以干扰其周期，防止癌细胞转移；鸡血藤的黄酮类成分具有保肝降酶的作用。

焦山楂：蔷薇科山楂属植物山里红或山楂的干燥成熟果实，主要成分为黄酮类 、三萜类、有机酸等，具有保护心血管系统的作用；在消化系统方面，可有效的促进消化酶的分泌和调节胃肠功能；也具有抗氧化和抗肿瘤的作用。

中国知网 https://www.cnki.net

鸡内金：脊索动物门鸟纲雉科动物鸡的干燥沙囊角质内壁，即鸡的内胃膜，主要成分为蛋白质、氨基酸、多糖和微量元素等。具有调节胃肠蠕动和消化液的分泌、提高消化酶活性、保护肠道的作用，可降低血糖、血脂和改善血液的高凝状态。

香附：莎草科植物莎草的干燥根茎，主要成分为挥发油类、黄酮类、生物碱类、糖类以及三萜类等，可通过造成癌细胞毒性使其凋亡发挥抗肿瘤的作用；除此之外，具有降血糖、抗血小板聚集、镇静催眠和解热镇痛抗炎等作用。

甘草：豆科植物甘草、胀果甘草或光果甘草的干燥根及根茎，主要成分为三萜类化合物甘草酸及其盐类、黄酮类化合物、甘草多糖等。在调节免疫、抗肿瘤、保肝、抗病毒等方面具有较好的疗效。

3.疗效分析

3.1 对血清肿瘤标志物 CEA、CA211 变化的分析

血清肿瘤标志物 CEA、CA211 指标的变化可有效的说明非小细胞肺癌患者疾病的进展情况。治疗前分别对两个指标的治疗组和对照组进行统计学检验，得到的结果均为 $P>0.05$，具有可比性。治疗后，治疗组和对照组的患者血清肿瘤标志物 CEA、CA211 水平均比治疗前有明显下降，且治疗组与对照组相比降低更加明显，均具有统计学意义（$P<0.05$）。说明治疗组在对照组常规化疗的基础上予以口服中药汤剂扶正抑瘤汤在降低非小细胞肺癌患者血清肿瘤标志物 CEA、CA211 方面与单纯化疗相比疗效更佳显著，尤其是方药中白花蛇舌草、半枝莲、薏苡仁的方药组成具有明显的抑制肿瘤生长、防止疾病发展的作用。

3.2 对中医证候改善的分析

本研究对中医证候分析显示，治疗后治疗组显效 10 例，有效 15 例，无效 4 例，总有效率 86.20%；对照组显效 4 例，有效 14 例，无效 10 例，总有效率 64.29%。经过统计学对数据进行分析检验后显示，治疗后治疗组和对照组的患者咳嗽、咳痰、咯血、气短、乏力、胸痛、食欲不振、便秘等症状均比治疗前有明显好转，且治疗组与对照组相比疗效更加明显，均具有显著统计学意义（$P<0.05$）。

扶正抑瘤汤以益气补血、活血化瘀为治疗原则，方中桔梗、苦杏仁、

中国知网 https://www.cnki.net

浙贝母、山慈菇可调节肺气宣发与肃降，止咳化痰，有效减轻咳嗽、咳痰症状；黄芪、太子参、炒白术、茯苓、当归具有补益气血作用，可有效改善气短、乏力不适症状；地龙、鸡血藤、香附通过行气活血缓解胸痛症状；焦山楂、鸡内金调理脾胃作用明显，以缓解食欲不振、便秘等消化系统不适症状。

3.3 对 KPS 评分改善的分析

通过本研究对 KPS 评分的分析显示，治疗前，治疗组和对照组比较不具有统计学意义（P>0.05）；治疗后，治疗组和对照组的患者 KPS 评分比治疗前有明显改善，且治疗组与对照组相比好转更加明显，均具有统计学意义（P<0.05）。说明扶正抑瘤汤在平衡气血阴阳，协调脏腑功能上疗效显著。从而缓解了患者的不适症状及体征以达到改善患者生存质量的目标。

3.4 对毒副反应的分析

西医化疗是抑制肿瘤生长、增殖、转移的重要治疗手段，但会引起骨髓抑制、恶心、呕吐、腹泻等不良反应。本研究对不良反应进行统计分析显示在白细胞、血小板、血红蛋白下降方面，治疗组明显轻于对照组，具有统计学意义（P<0.05），对恶心、呕吐、腹泻胃肠道反应的治疗治疗组明显优于对照组，具有统计学意义（P<0.05），在肝肾功能方面治疗后治疗组优于对照组，但不具有统计学意义（P>0.05）。

扶正抑瘤汤中黄芪、太子参、炒白术、当归具有补益气血的作用，可在一定程度上刺激白细胞及血红蛋白生成，以提高机体的免疫力和提高细胞的造血功能；地龙、鸡血藤具有活血化瘀功效，有抑制血小板聚集，防止癌栓形成的作用，以防止疾病的进一步发展；炒白术、茯苓、焦山楂、鸡内金、甘草能调节胃肠功能，对防治恶心、呕吐、腹泻等化疗后的毒副反应疗效明显。总而言之，扶正抑瘤汤能有效的预防骨髓抑制、恶心、呕吐等化疗后的毒副反应，对肝肾功能未见不良影响。

4.问题与展望

通过对气虚血瘀型非小细胞肺癌患者进行常规化疗与化疗联合扶正抑瘤汤后的治疗效果对比观察到口服中药汤剂联合化疗在降低肿瘤标志物、改善中医证候及患者生存质量和改善不良反应方面疗效显著。但由于条件的限制，本次临床研究样本量较小，观察时间短，并且未对患者进行远期

中国知网 https://www.cnki.net

疗效的随访记录，在今后的研究中应进一步扩大患者的样本量，增加实验结果的可信度，同时做好随访工作，完善随访机制以便了解患者远期疗效情况。在中药方剂组成方面需要对现代药理研究的学习和探索更加深入，为扶正抑瘤汤治疗气虚血瘀型非小细胞肺癌化疗患者的疗效分析提供有力证明。

中国知网 https://www.cnki.net

结　论

1、扶正抑瘤汤联合化疗能明显降低气虚血瘀型非小细胞肺癌患者血清肿瘤标志物 CEA、CA211 的水平；

2、扶正抑瘤汤能够改善气虚血瘀型非小细胞肺癌化疗患者的中医证候，并提高其生存质量；

3、扶正抑瘤汤能够改善气虚血瘀型非小细胞肺癌化疗患者的白细胞、血小板、血红蛋白计数的下降及消化道不良反应，对肝肾功能未见明显影响。

中国知网　https://www.cnki.net

致 谢

2011年9月满怀憧憬的来到黑龙江中医药大学,开启了我的医学之路,就此与母校结下了八年的缘分。本科五年始终不忘初心，竭尽全力学习专业知识，成功考取了硕士研究生。如今时光飞逝，如白驹过隙，研究生三年的生活也接近尾声。回首这三年，自己何其幸运，在良师益友的陪伴下成长、成熟。

感谢我的导师刘松江老师,老师如父亲一般关心着我们的学习和生活。在学习上尤其感谢老师给我们在门诊跟师的机会，通过两年多在门诊的学习，接触了上千例肿瘤病例，不仅为本次课题研究提供了非常大的帮助，也在老师的无私授予下对各种肿瘤疾病的诊治有了更深入的学习和研究，收获颇丰。在生活上，老师和师母更是让我们感受到有如父母一样的温暖，我们的师门是一个非常有凝聚力又有爱的大家庭，有任务一起完成，遇到问题一起解决，成功的喜悦一起分享。

感谢我的师兄师姐、师弟师妹和同届的姐妹们，三年的相处我们结下了深厚的情谊。感谢李雨师兄、王鹏师兄、闫珺师姐和庞雪莹师姐及其他各位师兄师姐在实习期间对我在专业知识上的耐心讲解和科研上的分享启发，让我深知理论与实践结合的重要性。感谢我的师弟师妹们三年中对我的支持和包容，感恩于大家的和谐相处和团结一心，一起出色的完成了师门内各项工作。感谢同届的小伙伴三年的陪伴，那些在一起学习、工作和娱乐的日子都会成为我最美好的记忆。

感谢我的父母二十几年来对我的养育，他们尊重我的想法，支持我的选择，不求回报的无私给予激励着我不断前行，不敢懈怠。希望可以用我专业的医学知识为他们的健康保驾护航，用我出色的成绩回报他们多年的默默付出。

感谢我的室友，一起生活的三年让我们彼此了解熟悉，学习和生活上对我的帮助都会铭记于心，经历的点点滴滴都是回忆。三年的时光，还有许多擦肩而过给予我帮助的老师和同学,对你们的感谢虽然不能一一表达,但都已经埋藏心中。

最后，感谢各位审评论文和答辩指导的教授和老师们，你们辛苦了！

中国知网 https://www.cnki.net

参考文献

[1] 吴菲，林国桢，张晋昕．我国恶性肿瘤发病现状及趋势[J]．中国肿瘤，2012,21(02):81-85.

[2] 陈万青，张思维，邹小农．中国肺癌发病死亡的估计和流行趋势研究[J]．中国肺癌杂志，2010,13(05):488-493.

[3] 陈万青，孙可欣，郑荣寿，等．2014 年中国分地区恶性肿瘤发病和死亡分析[J]．中国肿瘤，2018,27(01):1-14.

[4] Ettinger DS, Akerly W, Bepler G, et al. Non-small cell lung cancer[J]. Natl Compr Cancer Net, 2010(7):740-801.

[5] Delbaldo C, Michiels S, Rolland E, et al. WITHDRAWN:second or third additional chemotherapy drug for non-small cell lung cancer in patients with advanced disease[J]. Cochrane Database Syst Rev, 2012(4):CD004569.

[6] 杨林，赵晓红，杨云梅．非小细胞肺癌治疗的新进展[J]．中华危重症医学杂志(电子版)，2017,10(06):416-420.

[7] Crabtree T, Denlinger C, Meyers B. Stereotactic body radiation-therapy versus surgical resection for stage I non-small cell lung cancer[J]. J Thorac Cardiovasc Surg, 2010,2(140):377-386.

[8] Palma D, Visser O, Lagerwaard F. Treatment of stage I NSCLC in elderly patients: A population-based matched-pair comparison of stereotactic radiotherapy versus surgery[J]. Radiother Oncol, 2011,101(2):240-244.

[9] 郁仁存．郁仁存中西医结合肿瘤学[M]．北京:中国协和医科大学出版社，2008.

[10] 于彬，顾恪波，王逊，等.孙桂芝治疗恶性肿瘤学术思想浅析[J].北京中医药，2017, 36(10):909-912.

[11] 顾军花，刘嘉湘.刘嘉湘教授“扶正治癌”理论核心及运用方法[J].中国中西医结合杂志，2017, 37(04):495-499.

[12] 刘丽坤．肺癌的病机及治法探讨[J]．中国中医基础医学杂志．2004,10(5):75-76.

[13] 王雄文，林龙，李佩华，等．周岱翰诊治肿瘤的中医学术思想探讨[J]．广州中医药大学学报，2015,32(04):762-764.

[14] 赵炜．李佩文．治疗肺癌的经验[J]．北京中医，2002,21(6):329.

[15] 王振华，孙桂芝．孙桂芝治疗肺癌经验[J]．辽宁中医杂志，2010,37(05):791-793.

中国知网 https://www.cnki.net

[16]梁姗姗，张玉，张青．郁仁存治疗非小细胞肺癌经验[J]．中医学报，2018,33(02):200-203.

[17]胡小梅，张培彤，杨宗艳，等．中晚期非小细胞肺癌患者中医证型分布规律研究[J]．中国肿瘤，2007，16(1):51-53.

[18]李本达．中晚期非小细胞肺癌化疗前后中医证型变化规律研究[D]．北京:北京中医药大学，2009.

[19]胡皓，蔡小平.朴炳奎治疗肺癌经验探讨[J].中国民间疗法，2017，25(05):10-12.

[20]张磊．国医大师周仲瑛治疗肺癌角药撷菁[J]．上海中医药杂志，2013,47(11):1-2.

[21]王笑民．郁仁存肿瘤临证带教实录[M].北京:北京科学技术出版社，2016:18.

[22]李晓娟，陈滨海，杨雪静，等．气虚血瘀证支气管肺癌患者“瘀毒”病机的临床研究[J].中华中医药杂志，2019,34(02):773-777.

[23]胡艳，侯安继，周莉，等．益气活血方对中晚期非小细胞肺癌高凝状态作用的临床研究[J]．辽宁中医杂志，2011,38(12):2330-2332.

[24]刘嘉湘，施志明，李和根，等．益肺抗瘤饮治疗271例非小细胞肺癌临床观察[J].上海中医药杂志，2001,(2):4-6.

[25]何立丽，孙桂芝．孙桂芝治疗肺癌经验[J]．北京中医药，2009,28(4):263-264.

[26]石玉春． 林洪生教授运用中医药治疗肺癌经验[J].中医药导报，2016，22(20):17-19.

[27]郑红刚，花宝金，朴炳奎．朴炳奎辨治肺癌学术思想与经验探析[J]．中医杂志，2010，51(4):304-305.

[28]周岱翰．益气除痰法延长非小细胞肺癌中位生存的作用[J]．中医杂志，2005,46(8):602.

[29]姜福胜，魏博，张广亮，等．康莱特注射液联合化疗治疗非小细胞肺癌 62 例临床观察[J]．临床医药实践，2010,19(3B):345-346.

[30]邹波峰，李康，王沈玉．艾迪注射液配合化疗治疗非小细胞肺癌的临床观察[J].实用肿瘤学杂志，2007,21(3):268- 269.

[31]邵海燕，陈君峰．复方斑蝥胶囊对晚期肺癌维持治疗效果的前瞻性研究[J]．中华中医药学刊，2014,32(3):686-688.

[32]田华琴，余寿益，王斌．鸦胆子油乳剂对非小细胞肺癌患者细胞免疫功能及生活质量的影响[J]．中国中西医结合杂志，2007,27(2):157-159.

中国知网 https://www.cnki.net

[33] 文淑珍，谭成乔．消癌平联合多西紫杉醇治疗晚期非小细胞肺癌的疗效研究[J]．中国医药指南, 2014,12(35):277-278.

[34] 俞杞泉，张嗣博，柳涛．术后化痰方干预非小细胞肺癌术后围手术期的多中心随机双盲对照研究[J]．上海中医药大学学报, 2014,28(2):23-25.

[35] 朱丽华，李和根，史美育，等．非小细胞肺癌根治术后无瘤生存期影响因素分析及中药干预效果评价[J]．上海中医药杂志, 2013,47(2):11.

[36] 寿伟臻，俞杞泉，朱正洪，等．金宁方加味对Ⅲ期非小细胞肺癌患者术后抗复发转移临床评价[J]．上海中医药杂志, 2014, 48(8):30-33.

[37] 李菁，陈慧．陈慧治疗肺癌术后经验[J]．实用中医药杂志, 2013,29(8):678.

[38] 侯恩存，王新，练祖平，等．中西医结合治疗Ⅲ、Ⅳ期非小细胞肺癌 33 例[J]．中医研究, 2007,20(2):33- 35.

[39] 李道阳，欧成茂，李国定，等．扶正排毒抗癌方对非小细胞肺癌化疗增效减毒作用的临床观察[J]．中国中西医结合杂志, 2000,20(3):208-209.

[40] 徐文姬，费玉明，何邦志．中西医结合治疗恶性肿瘤胸腔积液疗效观察[J]．现代中西医结合杂志, 2007,16(1):50.

[41] 周岱翰．中西医结合治疗非小细胞肺癌近期疗效观察[J]．中国中西医结合杂志, 2005,25(12):1061-1065.

[42] 刘莉，徐凯．中药与化疗治疗老年晚期非小细胞肺癌 60 例[J]．中国中医药现代远程教育, 2014,12(1):55-56.

[43] 孙太振，朱霞，杨峰．中西医结合治疗放射性肺炎 48 例临床观察[J]．中原医刊, 2006,33(22):45-46.

[44] 蔡凯，刘俊波，黄常江，等．益气养阴中药对三维适形放疗局部晚期非小细胞肺癌老年患者生活质量及免疫功能的影响[J]．西部中医药, 2012,25(11):1.

[45] 邓生明，林小清，张好平．天龙消瘤方配合 125 I 粒子植入治疗肺癌疗效观察[J]．陕西中医, 2011,32(8):939-940.

[46] 高启龙，杨峰，刘怀民，等．益气散结攻毒方治疗化疗及靶向药物无效晚期非小细胞肺癌 35 例[J]．中医研究, 2014,27(3):25.

[47] 郭鹏，王荔源．特罗凯相关药物性皮疹肺癌患者的中医体质因素特点分析[J]．内蒙古中医药, 2012,31(4):141-142.

中国知网 https://www.cnki.net

附 录

附表-1 国际肺癌协会（IASLC）2017 年第 8 版肺癌分期标准

TNM 分期标准

T 分期：

TX：未发现原发肿瘤，或者通过痰细胞学或支气管灌洗发现癌细胞，但影像学及支气管镜无法发现。

T0：无原发肿瘤的证据。

Tis：原位癌。

T1：肿瘤最大直径≤3cm，周围包绕肺组织及脏层胸膜，支气管镜见肿瘤侵及叶支气管，未侵及主支气管。

T1a：肿瘤最大径≤1cm；

T1b：肿瘤最大径>1cm，≤2cm；

T1c：肿瘤最大径>2cm，≤3cm。

T2：肿瘤最大径>3cm，≤5cm；侵犯主支气管（不常见的表浅扩散型肿瘤，不论体积大小，侵犯限于支气管壁时，虽可能侵犯主支气管，仍为 T1），但未侵及隆突；侵及脏胸膜；有阻塞性肺炎或者部分肺不张。符合以上任何一个条件即归为 T2。

T2a：肿瘤最大径>3cm，≤4cm；

T2b：肿瘤最大径>4cm，≤5cm。

T3：肿瘤最大径>5cm，≤7cm。直接侵犯以下任何一个器官，包括：胸壁（包含肺上沟瘤）、膈神经、心包；全肺肺不张肺炎；同一肺叶出现孤立性癌结节。符合以上任何一个条件即归为 T3。

T4：肿瘤最大径>7cm，无论大小，侵及以下任何一个器官，包括：纵隔、心脏、大血管、隆突、喉返神经、主气管、食管、椎体、膈肌；同侧不同肺叶内孤立癌结节。

N 分期：

NX：区域淋巴结无法评估。

N0：无区域淋巴结转移。

N1：同侧支气管周围及（或）同侧肺门淋巴结以及肺内淋巴结有转移，包括直接侵犯而累及的。

中国知网 https://www.cnki.net

N2:同侧纵隔内及（或）隆突下淋巴结转移。

N3：对侧纵隔、对侧肺门、同侧或对侧前斜角肌及锁骨上淋巴结转移。

M 分期：

MX：远处转移不能被判定。

M0：没有远处转移。

M1：远处转移。

M1a：局限于胸腔内，包括胸膜播散（恶性胸腔积液、心包积液或胸膜结节）以及对侧肺叶出现癌结节（许多肺癌胸腔积液是由肿瘤引起的，少数患者胸液多次细胞学检查阴性，既不是血性也不是渗液，如果各种因素和临床判断认为渗液和肿瘤无关，那么不应该把胸腔积液纳入分期因素）。

M1b：远处器官单发转移灶为 M1b；

M1c：多个或单个器官多出转移为 M1c。

中国知网 https://www.cnki.net

附表-2 中医证候积分标准

症状	无（0分）	轻（1分）	中（2分）	重（3分）
咳嗽	无	偶尔咳嗽	间断咳嗽，需药物治疗	咳嗽频作，影响睡眠，依赖药物
咳痰	无	量少易咳	量较多，用力可咯出	量多，粘稠难咯
咯血	无	痰中有血丝	痰中带血	咯血
气短	无	稍感气短	动则气短	气短明显，不动也喘
乏力	无	可坚持体力劳动	勉强坚持日常工作	无法坚持日常工作
胸痛	无	偶有隐痛	疼痛频发	剧痛难忍
食欲不振	无	食量不减，但觉乏味	食量减少 1/3	食量减少 1/2 以上
便秘	无	大便干结，每日一行	大便秘结，两日一行	大便艰难，数日一行

中国知网 https://www.cnki.net

附表-3 卡诺夫斯基（Karnofsky）健康状况评分表

体力状况	记分
身体无任何不适症状及体征，一切正常	100
可以进行正常活动，但有轻微症状或体征	90
勉强可以维持正常活动，有部分症状或体征出现	80
可以生活自理，但无法维持正常生活和工作	70
大部分生活可以自理，偶尔有需要他人帮助	60
生活常需他人照料，且经常需要医疗护理	50
生活无法自理，并且需要他人特别照顾和帮助	40
生活能力严重失去，需住院，但尚不危及生命	30
病重，需要住院以及积极的支持治疗	20
生命垂危，濒临死亡	10
死亡	0

中国知网 https://www.cnki.net

附表-4 WHO 化疗急性和亚急性毒副反应分度标准

观察指标	0 度	Ⅰ度	Ⅱ度	Ⅲ度	Ⅳ度
白细胞（$\times10^9$/L）	≥4.0	3.0～3.9	2.0～2.9	1.0～1.9	＜1.0
血小板（$\times10^9$/L）	≥100	75～99	50～74	25～49	＜25
血红蛋白（g/L）	≥100	95～109	80～94	65～79	＜65
谷丙转氨酶	≤1.25×N	1.26～2.50×N	2.6～5.0×N	5.1～10.0×N	>10×N
肌酐	≤1.25×N	1.26～2.50×N	1.26～2.50×N	5.1～10.0×N	>10×N
恶心呕吐	无	恶心	暂时性呕吐	呕吐，需治疗	难控制的呕吐
腹泻	无	短暂（＜2 天）	能忍受（>2 天）	不能忍受，需治疗	血性腹泻

中国知网 https://www.cnki.net

中国知网 https://www.cnki.net

中国知网 https://www.cnki.net
