# Supplementary material for: Benefit-risk evaluation of Fuzheng Yiliu Decoction combined with chemotherapy for treating non-small cell lung cancer using multicriteria decision analysis
Source: Front Oncol. 2025 Sep 23;15:1627904. doi: 10.3389/fonc.2025.1627904 (PMC12500661; doi:10.3389/fonc.2025.1627904)
Supplement: Supplementary file 2 [file Supplementaryfile2.zip › supplementary files/Included literature.zip!/literature/纳入文献/5吕鹏强.pdf]

分类号： 密级：

硕 士 研 究 生 学 位 论 文

论文题目（中文） 裴氏扶正抑瘤汤配合化疗治疗老年晚期非小细胞肺癌临床观察

论文题目（外文） Clinical Study on Advanced Non-small Cell Lung Cancer Treated by pei shi fu zheng yi liu tang Combined with Chemotherapy

研 究 生 姓 名 吕鹏强

学 科、专 业 中西医结合临床

研 究 方 向 中西医结合防治肿瘤

导师姓名、职称 蒲朝晖主任医师

论 文 工 作

起 止 年 月 2015 年 02 月 至 2017 年 03 月

论文提交日期 2017 年 03 月

论文答辩日期 2017 年 05 月

学位授予日期 2017 年 06 月

(C)1994-2022 China Academic Journal Electronic Publishing House. All rights reserved. http://www.cnki.net

独创性声明

本人声明所呈交的学位论文是本人在导师的指导下进行的研究工作及取得的研究成果。学位论文中凡应用他人已经发表或未发表的成果、数据、观点等，均已明确注明出处。除文中已经注明引用的内容外，不包含任何其他个人或集体已经发表或撰写过的科研成果。对本研究做出贡献的个人或集体均已在文中做了明确的说明并表示谢意。

论文作者签名：[illegible]　　签字日期：2017 年 6 月 1 日

学位论文版权使用授权书

本学位论文作者完全了解甘肃中医药大学有关保留、使用学位论文的规定，本人同意学校保存或向国家有关部门或机构送交论文的纸质版或电子版，允许论文被查阅或借阅。本人授权甘肃中医药大学可以将学位论文的全部内容或部分内容编入有关数据库进行检索，可以采用任何复制手段保存、汇编学位论文。

（保密的学位论文在解密后适用本授权书）

学位论文作者签名：[illegible]　　签字日期：2017 年 6 月 1 日

导师签名：[illegible]　　签字日期：2017年 6 月 1 日

(C)1994-2022 China Academic Journal Electronic Publishing House. All rights reserved. http://www.cnki.net

目 录

摘 要……1
ABSTRACT……2
符号说明……4
前言……6
一 临床研究内容……8
1 研究目的……8
2 临床资料……8
3 研究方法……10
4 观察指标……11
5 疗效评定标准……11
6 安全性指标……12
7 随访……13
8 伦理学要求……13
9 技术路线图……13
二 临床研究结果……14
1 两组受试者基线水平比较……14
2 临床疗效结果……16
3 安全性指标……21
讨论……22
1 中医对老年晚期非小细胞肺癌认识……22
2 裴正学教授论治老年晚期非小细胞肺癌思想……22
3 方药分析……23
4 研究结果分析……27
结语……29
参考文献……30
附表……33
病例登记报告表……41
文献综述……56

(C)1994-2022 China Academic Journal Electronic Publishing House. All rights reserved. http://www.cnki.net

老年晚期非小细胞肺癌的中西医研究进展......56
参考文献......64
致谢......68
研究成果......69

(C)1994-2022 China Academic Journal Electronic Publishing House. All rights reserved. http://www.cnki.net

摘 要

目的：观察裴氏扶正抑瘤汤对老年晚期非小细胞肺癌(NSCLC)患者生存质量和化疗毒副作用的影响，评价裴氏扶正抑制瘤汤治疗老年晚期 NSCLC 的有效性及安全性。

方法：将 60 例符合标准的老年晚期 NSCLC 患者随机分为两组，治疗组和对照组各 30 例，治疗组给予西医 NP 方案化疗联合裴氏扶正抑瘤汤口服，对照组给予单纯西医化疗。两组治疗周期均为 3 个疗程，随访 3 月。比较两组治疗前后目标病灶大小、肿瘤标志物的变化、毒副反应的发生以及 KPS 评分的变化情况；观察两组治疗前后 ALT、AST、BUN、Cr 及尿、粪常规、心电图等指标的变化。

结果：（1）治疗组和对照组 CR 患者为 0 例，治疗组 PR 为 13 例，SD 为 11 例，对照组 PR 为 9 例，SD 为 11 例，两组瘤体稳定了率分别是 80.0%和 66.7%。治疗组有 6 例发生疾病进展，对照组有 10 例发生疾病进展。两组肿瘤瘤体变化比较，$P=0.197>0.05$；（2）治疗前后两组肿瘤标志物 CEA、CY21-1 均较大幅度降低，但两组间比较显示 $P>0.05$；（3）治疗后两组骨髓抑制相比较，治疗组骨髓抑制发生及程度低于对照组，$P<0.05$；（4）相比于对照组，治疗组可以更好的改善中医临床症状和患者的生存质量，两组比较 $P<0.05$；（5）安全性评价：比较两组患者治疗前后 ALT、AST、BUN、Cr，无统计学差异（$P>0.05$），无严重不良事件发生。

结论：裴氏扶正抑瘤汤能够较为显著的改善老年晚期 NSCLC 患者的生存质量，减缓化疗毒副作用的发生，使患者对化疗的耐受性加强，安全性良好。

关键词：裴氏扶正抑瘤汤；生存质量；毒副作用；老年晚期非小细胞肺癌

(C)1994-2022 China Academic Journal Electronic Publishing House. All rights reserved. http://www.cnki.net

ABSTRACT

Objective:

This clinical research aims to observe the effect of Pei shi fu zheng yi liu tang on improving quality of life, alleviating major clinical symptoms and Side Effects of Chemotherapy of Senior Advanced No-small cell Lung cancer, to evaluate the effectiveness and safety of Pei shi fu zheng yi liu tang objectively.

Methods:

All those 60 cases met the standards of patients with Senior Advanced No-small cell Lung cancer come from Gansu Province Tumor hospital since January to November of 2016. 60 cases were randomly divided into two groups :30 cases of treatment group and 30 cases control group. There is no significant difference in sex ,age, pathology, clinical stages, source position and transfer number between the treatment group and the control group ($P>0.05$). The two groups accepted the same supporting treatment. Meanwhile, the control group was treated with conventional chemotherapy alone, the treated group was treated with Pei shi fu zheng yi liu tang on the basis of conventional chemotherapy, last 3 period of treatment and follow-up 3 months. The therapeutic efficiency and adverse effect of the two groups were evaluated after curing 60 days, then estimated it clinical effect and safety.

Results:

All the patients in the two groups were finally finished in the research. Results : (1) The recent clinical effect: No cases reached CR equally in the two groups. The treatment group and control group tumors reached a stable rate of 80.0% and 66.7% (PR+SD) respectively. There is no notable difference between the two groups in stabilizing the tumor. 6 cases with disease progression in the treatment group and 10 cases in control group. (2) There is no significant difference to CEA and CY21-1 in the two groups before observation. After treatment, both CEA and CY21-1 in the two had obvious gone down, but the difference is not notable.($P > 0.05$). (3) Significant difference can be seen in bone marrow suppression between two groups($P < 0.05$). (4) Comparing with the control group, the treatment group can make Chinese clinical symptoms and KPS score batter.($P < 0.05$) (5)Safety evaluation: comparison between the two groups shows that there is no significant difference on renal

(C)1994-2022 China Academic Journal Electronic Publishing House. All rights reserved. http://www.cnki.net

function, urine routine and stool-routine, no serious adverse event was happened, suggesting the safety of Pei shi fu zheng yi liu decoction.

Conclusion:

Pei shi fu zheng yi liu tang combined with the chemotherapy in treating Senior advanced No-small cell Lung cancer can improve patients, quality of life and TCM symptoms significantly, reduce the levels of tumor related markers. Also it help patients endure chemotherapy by relieving the side effect resulted from chemotherapy drugs. The security is good, no obvious toxicity was observed, so it has a high value on clinical application.

Key Words： advanced NSCLC ; Pei shi fu zheng yi liu tang; chemotherapy; traditional Chinese medicine

(C)1994-2022 China Academic Journal Electronic Publishing House. All rights reserved. http://www.cnki.net

符号说明

英文缩写	英文全称	中文名称
PHC	Primary Lung Cancer	原发性肺癌
NSCLC	Non-small Cell Lung Cancer	非小细胞肺癌
ALC	Adwanced Lung Cancer	晚期肺癌
CEA	Carcinoembryonic antigen	癌胚抗原
CY21-1	Serum Cell Keratin 19 pieces	血清角质蛋白 19 片段
TNM	Tumor Node Metastasis	肿瘤分期系统
UICC	Union for International Cancer Control	国际抗癌联盟
AJCC	American Joint Committee on Cancer	美国癌症分期联合委员会
IASLC	International Association for the Study of Lung Cancer	国际肺癌研究协会
RECIST	Response Evaluation Criteria In Solid Tumors	肿瘤实体疗效评价
WHO	World Health Organization	世界卫生组织
ALT	Alanine aminotransferase	谷丙转氨酶
AST	Aspartate transaminase	谷草转氨酶
PFS	Progression-free survival	无进展生存期
KPS	Karnofsky performance status	卡式功能状态评分
ASCO	American Society of Clinical Oncology	美国临床肿瘤学会
BUN	Blood urea nitrogen	尿素氮
Cr	Creatinine	肌酐
OS	Overall survival	总生存期
QOL	Quality Of Life	生存质量
SPSS	Statistical Product and Service Solutions	统计产品与服务解决方案
CT	Computed Tomography	电子计算机断层扫描
CRF	Case Report Form	病例报告表
WBC	White blood cell	白细胞
HGB	Hemoglobin	血红蛋白

(C)1994-2022 China Academic Journal Electronic Publishing House. All rights reserved. http://www.cnki.net

NEU	Neutrophilic granulocyte	中性粒细胞
PLT	Platelet	血小板
APS	Astragalus Polysacharin	黄芪多糖
HIF-1	Hypoxia Inducible Factor-1	缺氧诱导因子-1
IL-2	Interleukin-2	白细胞介素-2
MRI	Magnetic Resonance Imaging	磁共振成像
TCM	Traditional Chinese Medicine	中医
TNF-α	Tumor Necrosis Factor-α	肿瘤坏死因子-α
VEGF	Vascular Endothelial Growth Factor	血管内皮生长因子

(C)1994-2022 China Academic Journal Electronic Publishing House. All rights reserved. http://www.cnki.net

前言

原发性肺癌(Primary Lung Cancer)是最常见的恶性肿瘤之一，严重危害人类生命及健康。世界卫生组织癌症研究署（International Agency for Research on Cancer，IARC）2014 年发布的报告显示：2012 年全球新发肺癌约 180 万，死亡约 160 万，分别占恶性肿瘤新发病例和死亡病例的 13%和 19.4%，占全部恶性肿瘤的第一位[1]。我国肿瘤登记中心于 2014 年发布了 2010 年登记的情况，显示新发肺癌患者为 60.59 万人(男性 41.63 万，女性 18.96 万)，其新发病例超过了先前多发的胃癌和肝癌，肺癌的新发病例占比达到了 19．59％(男性 23.03％，女性 14.75％)，居于首位(男性首位，女性第 2 位)。同期，死于肺癌的人数为 48.66 万(男性 33.68 万，女性 16.62 万)，在因恶性肿瘤致死的比例中达到 24.87％(男性 6.85％，女性 21.32％)。报告显示，同期我国肺癌发病率和死亡率分别是 35.23／10 万(男性 49.27／10 万，女性 21.66／10 万)和 27.93／10 万(男性 39.79／10 万，女性 16.62／10 万)[2]。非小细胞肺癌在肺癌的病理分型中占比最高，是其总数的 80~85%，在这部分患者中，约 50%的患者年龄在 65 岁以上，30~40%的患者年龄在 70 岁以上，80 岁以上者约占 10%[3]。

近年来，随着我国社会老龄化及环境污染等问题的加重，老年肺癌的发病占比逐年上升。该病起病隐匿，且老年人免疫功能低下，早期临床表现不明显，大部分肺癌经发现时已进展至中晚期，预后不佳。

目前，肺癌首选治疗方案为手术治疗。以放化疗为主的辅助治疗是老年晚期非小细胞肺癌（NSCLC）的主要治疗措施。研究表明，单药方案已经不适合目前肺癌治疗的需求，联合化疗的优势渐渐显露。肺癌患者在晚期时临床症状明显，在此基础上接受放疗、化疗，病人耐受性差，毒副反应更为明显。通过大量研究证实，在内科领域以中西医结合治疗恶性肿瘤是行之有效的模式，且中医药可以减轻放化疗的不良反应，控制病情发展，对提高生存质量有很大辅助作用。

我国著名的中西医结合专家裴正学教授重视中西医之间的互补优势，提出的“西医诊断，中医辨证，中药为主，西药为辅”的思想，为中西医结合临床实践指明了方向。裴老指出肺癌的发病以正虚为主，治疗以扶正固本为大法，正如《素问》“正气存内，邪不可干”、“ 邪之所凑，其气必虚”所论。同时兼顾正邪之间的关系，分阶段论治。裴老强调“炎症是肿瘤的好朋友”，认为肺癌的发生发展与炎症关系密切，中医应该关注脏腑气血阴阳之间的关系。认为辨证论治的结果就是治本，是“围点打援”的战略，

(C)1994-2022 China Academic Journal Electronic Publishing House. All rights reserved. http://www.cnki.net

能够围绕肿瘤解决众多周边效应，从而极大的提高患者的生存质量和延长无进展生存时间（PFS）和总生存期（OS）。扶正固本之法以健脾补肝肾为其精髓，治疗中兼顾放化疗后毒热伤阴、六淫邪气、热毒炽盛、气滞血瘀等证。用药以裴氏扶正抑瘤汤为基本方，临床随症加减，屡获良效。

裴氏扶正抑瘤汤在长期临床应用中取得了满意的疗效，得到了患者高度的评价和认可，在省内外享有一定的知名度。余跟随老师门诊，亲历其桴鼓之效，为进一步探讨裴氏扶正抑瘤汤之作用，为临床应用及更为深入的实验研究提供数据资料，故立题研究。

(C)1994-2022 China Academic Journal Electronic Publishing House. All rights reserved. http://www.cnki.net

一 临床研究内容

1 研究目的

对比裴氏扶正抑瘤汤联合 NP 方案和单纯 NP 方案治疗老年晚期 NSCLC 患者，评价裴氏扶正抑瘤汤的有效性及安全性。

2 临床资料

2.1 病例来源

研究纳入的 60 例研究对象，均来自于 2016 年 1 月至 2016 年 11 月在甘肃省肿瘤医院（三甲医院）中西医结合科就诊患者。

2.2 诊断标准

2.2.1 西医诊断标准

参照国家卫生和计划生育委员会编写的《中国原发性肺癌诊疗规范》（2015 年版）[2]中的标准制定。NSCLC 的 TNM 分期采用国际肺癌研究协会(International Association for the Study of Lung Cancer，IASLC)2009 年第七版分期标准(IASLC 2009)。（见附表 1、2）

2.1.1.1 病理学诊断标准

（1）肺手术标本经病理、组织学证实者；

（2）经肺针穿刺或纤支镜采得肺或支气管活检组织标本，经组织学诊断为原发性支气管肺癌者；

（3）颈部、腋下淋巴结，胸壁、胸膜或皮下结节等转移灶活检，组织学表现符合原发性支气管肺癌，且肺或支气管壁内有肺癌存在，临床上又能排除其他器官原发癌者。

2.1.1.2 细胞学诊断

痰液、纤支镜毛刷、抽吸、冲洗等获得细胞学标本，镜下所见符合肺癌细胞学标准者，诊断可以确立。需排外上呼吸道或食管肿瘤。

2.1.1.3 临床诊断标准

根据临床症状、体征及影像学检查，符合下列情况之一者可作为原发性肺癌的临床诊断：

（1）胸部 X 线检查发现肺部孤立性结节或肿物，有时有毛刺；

（2）肺癌高危人群，有咳嗽或血痰，胸部 X 线检查发现局限性病变，经积极抗炎或抗结核治疗（2-4 周）无效或病变增大者；

(C)1994-2022 China Academic Journal Electronic Publishing House. All rights reserved. http://www.cnki.net

（3）节段性肺炎在 2-3 个月内发展成为肺叶不张，或肺叶不张短期内发展成为全肺不张；

（4）短期内出现无其他原因的一侧增长性血性胸水，或一侧多量血性胸水同时伴肺不张者或胸膜结节状改变者；

（5）明显咳嗽、气急，胸片显示双肺粟粒样或弥散性病变，可排除粟粒型肺结核、肺转移瘤、肺真菌病者；

（6）胸片发现肺部肿物，伴有肺门或纵隔淋巴结肿大，并出现上腔静脉阻塞、喉返神经麻痹等症状，或伴有远处转移表现者。

2.2.2 中医诊断标准

中医诊断标准参照 2002 年《中药新药临床研究指导原则》[2]之原发性肺癌篇中的肺癌症候诊断标准拟定：

主症：咳嗽、咳痰、痰中带血、气喘、胸痛

次症：神疲乏力，面色白，形体消瘦，脘腹痞满，大便溏薄或干结；口干，腰膝酸软，头晕目眩，耳鸣；

舌脉：舌淡或淡胖，苔薄白或薄黄，脉细弱或细数。

以上必备主症，加次症两项，参照舌脉即可诊断。

2.3 纳入标准

(1) 符合诊断的晚期（ⅢB 期、Ⅳ期）NSCLC 患者；

(2) 符合气血亏虚，邪毒留滞证型标准；

(3) 符合化疗适应症；近 1 月未行放疗或化疗治疗；

(4) 有一个可以精确测量的病灶（病灶螺旋 CT 或 MRI＞10mm）；

(5) 年龄在 60 至 75 岁之间；

(6) KPS 评分>60 分者；

(7) 预计生存期在 3 个月以上；

(8) 愿意参加本次试验研究，详细告知其相关内容，签署知情同意书者。

2.4 排除标准

(1) 年龄<60 岁或>75 岁；

(2) 危重患者或预期生存期<3 个月的患者；

(3) 严重的心、肝、肾、脑等相关脏器疾病及骨髓造血功能疾病者；

(4) KPS 评分<60 分者；

(C)1994-2022 China Academic Journal Electronic Publishing House. All rights reserved. http://www.cnki.net

(5) 近 1 周内服用过类似药物而影响疗效者；

(6) 对多种药物过敏或者过敏体质者。

2.5 剔除标准

(1) 没有根据既定方案行规范用药，无法判定其临床疗效者；

(2) 收集的数据资料不全，影响评估者；

(3) 治疗期间因严重毒性反应停药者；

(4) 治疗期间因疾病进展更换方案者。

2.6 脱落标准

已经通过筛选，并签署知情同意书，纳入临床研究的全部合格受试者，无论其出于任何原因中途退出试验研究，只要没有完成设计方案中所规划的治疗周期者，均做脱落病例处理。

(1)纳入的合格病例要求中途退出者；

(2)试验中失访或失联者；

(3)治疗中出现严重并发症或者严重不良事件者。

3 研究方法

3.1 分组方法

将符合标准的 60 例患者，按照就诊顺序编号。用 SPSS 21.0 产生随机数字，从任意一个数开始，沿同一方向连续取 60 个随机数字，用取得的随机数字除以组数，整除者分入治疗组，有余数者分入对照组，不平衡者再做调整，得治疗组和对照组每组各 30 例患者。

3.2 试验方法

治疗组：化疗方案采用 NP 方案：长春瑞滨（法国皮尔·法伯药物研制公司，10mg/支，注册证号 H20030660） 25mg/m^2 静脉滴注，第 1 天和 8 天；顺铂（齐鲁制药有限公司，10mg/支，国药准字 H37021358） 30mg/m^2 静脉滴注，第 1、2、3 天。并进行水化、利尿、止吐等处理措施。3 周为 1 周期，共 3 个周期。治疗期间在化疗基础上加用裴氏扶正抑瘤汤中药水煎剂，日一剂，分早晚饭后温服，每次 150ml，2 次/日。

裴氏扶正抑瘤汤方药组成：

北沙参 15g	半枝莲 15g	人参须 15g	女贞子 15g	黄　芪 30g
山萸肉 15g	生　地 12g	丹　参 30g	当　归 10g	白　芍 10g

(C)1994-2022 China Academic Journal Electronic Publishing House. All rights reserved.   http://www.cnki.net

潞党参 15g 旱莲草 15g 白花蛇舌草 15g 龙 葵 15g 太子参 15g

对照组：长春瑞滨 25mg/m^2 静脉滴注第 1 天和 8 天；顺铂 30mg/m^2 静脉滴注第 1、2、3 天。并进行水化、利尿、止吐等处理措施。3 周为 1 周期，共 3 个周期。

3.3 统计学处理

采用 SPSS21.0 统计软件进行数据处理，服从正态或近似正态分布的计量资料用均值±标准差（$\bar{x}$±s）表示，两独立样本组间比较使用两独立样本 *t* 检验，配对样本组间比较使用配对样本 *t* 检验，计数资料采用卡方检验或 Fisher 确切概率法进行分析，等级资料采用非参数秩和检验进行处理，$P<0.05$ 为差异有统计学意义。

4 观察指标

4.1 疗效指标

(1) 白细胞、血红蛋白、血小板；

(2) 消化道反应；

(3) 中医临床症候积分；

(4) KPS 积分；

(5) 生存质量评分；

(6) 目标瘤体变化；

(7) 血清 CEA，CY21-1 变化。

4.2 安全指标

(1) 尿常规；

(2) 粪常规；

(3) 肝功能（ALT、AST）；

(4) 肾功能（BUN、Cr）。

5 疗效评定标准

5.1 骨髓抑制

每周检查血常规，记录白细胞、血红蛋白、血小板计数，比较治疗前后治疗组和对照组在骨髓抑制方面的差异。（化疗后骨髓抑制的分度见附表 3）

5.2 消化道反应

根据病情可以将患者的消化道反应评定为 0-4 级，评定指标：显效：积分下降≥2 分；有效：积分下降；无效：积分无变化或升高。（消化道反应分度见附表 3）

(C)1994-2022 China Academic Journal Electronic Publishing House. All rights reserved. http://www.cnki.net

5.3 中医临床症候评分

观察患者治疗前后的中医临床症候积分变化，并进行统计学分析比较，具体的积分办法参见附表 4。

积分比＝(治疗前总积分－治疗后总积分) / 治疗前总积分×100%。

显效：70%≤积分比＜100%；有效：30%≤积分比＜70%；无效：未达到有效标准者。

5.4 体力状况评分

体力状况的评定以 Karnofsky 评分为主（见附表 5），观察治疗前后 Karnofsky 评分的变化，进行统计学分析。

5.5 肺癌患者生存质量

治疗前后各评估一次。使用肺癌患者生存质量测定量表（FACT-L）评估（生存质量总积分及身体状况、社会/家庭状况、与医生的关系、情绪状况、功能状况、症状/副作用等 6 个部分积分），每个条目由低至高 0-4 分，分别计算治疗前后生存质量积分的均值，比较治疗前后生存质量变化。（见附表 6）

5.6 肿瘤客观疗效标准

根据肿瘤实体疗效评价 RECIST(Response Evaluation Criteria In Solid Tumors)标准（见附表 7）。分别在治疗前后行胸部 CT 检查，记录目标瘤体的最大径总和的变化，完全缓解（CR）：全部病灶消失维持 4 周；部分缓解（PR）：缩小 30%维持 4 周；病情稳定（SD）：非 PR/PD；病情进展（PD）增加 20%，病灶增加前非 CR/PR/SD。 进行统计处理。

有效率=[(CR+PR)/(CR+PR+NC+PD)]×100%

病灶稳定率=[(CR+PR+SD)/(CR+PR+SD+PD)]×100%

5.7 肿瘤标志物

观察治疗前后两组 CEA、CYRF21-1 数值的变化，并详细记录治疗前后检测值的变化。

(C)1994-2022 China Academic Journal Electronic Publishing House. All rights reserved. http://www.cnki.net

6 安全性指标

6.1 肝、肾功能变化

每周查肝功能和肾功能，记录 ALT、AST、BUN、Cr 的变化情况，其分度参照附表 3。

6.2 尿、粪常规变化

每周查尿、粪常规，观察并记录是否有膀胱和肠道的损害，其分度参照附表 3。

7 随访

按照计划对患者进行定期随访，记录相关情况。

8 伦理学要求

本次研究遵循其赫尔辛基宣言的原则，无论受试者进入哪一组，都可以得到较为全面的治疗，并且签署知情同意书。

9 技术路线图

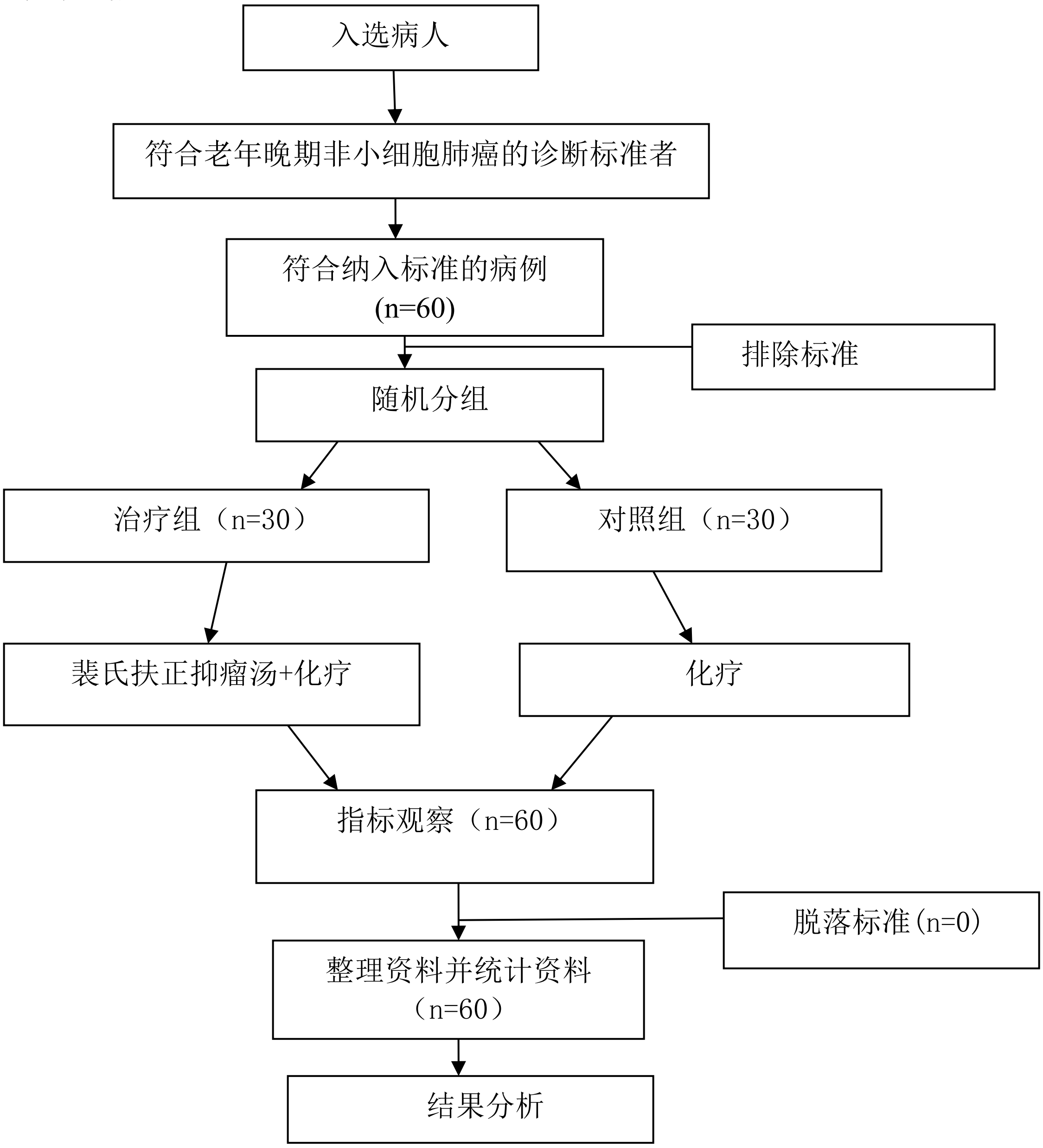

(C)1994-2022 China Academic Journal Electronic Publishing House. All rights reserved. http://www.cnki.net

二 临床研究结果

1 两组受试者基线水平比较

1.1 两组受试者性别、年龄比较

性别比较采用卡方检验，得χ^2=0.341，P=0.559，$P > 0.05$。年龄用（ $\bar{\chi}$±s ）（单位：岁）表示，比较采用 t 检验，得 t=0.591，P=0.557，$P > 0.05$，具有可比性。（表 1）

表 1　两组患者年龄及性别情况比较

组别	例数	性别		年龄（岁）
		男	女	
治疗组	30	23	7	68.00±3.051
对照组	30	21	9	68.53±3.884

注：性别：χ^2=0.341，P=0.559；年龄：t=0.591，P=0.557。

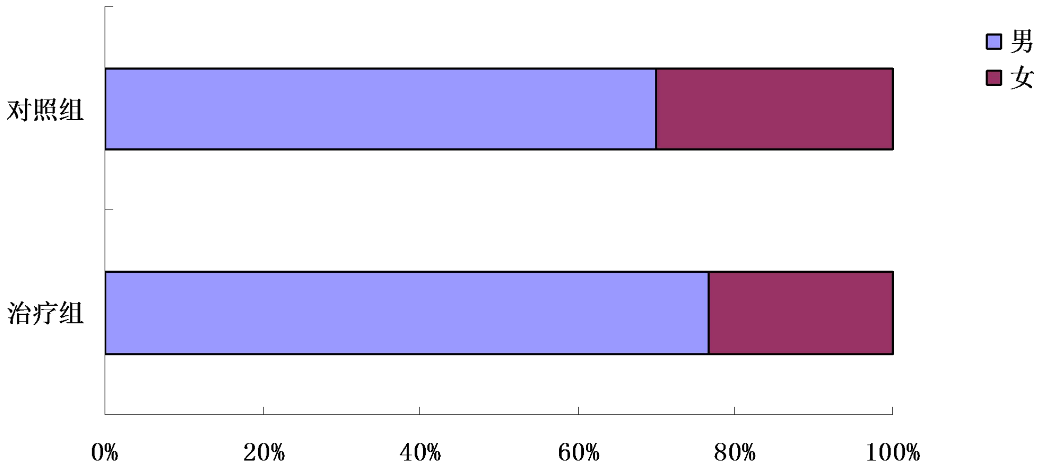

图1　两组性别比较

(C)1994-2022 China Academic Journal Electronic Publishing House. All rights reserved.　http://www.cnki.net

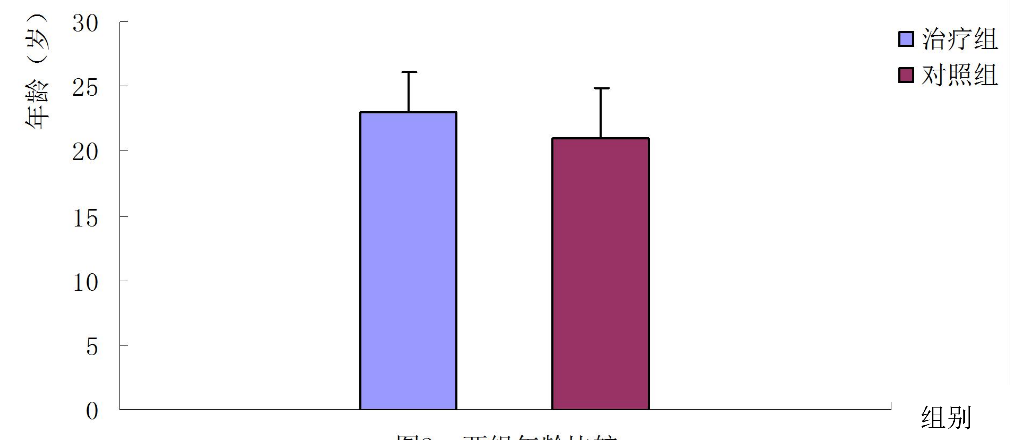

图2　两组年龄比较

1.2 两组患者病理分型及临床分期比较

病理分型构成比较采用卡方检验，得χ^2=0.067，*P*=0.795，*P*＞0.05，临床分期比较采用卡方检验，得χ^2=0.098，*P*=0.754，*P*＞0.05，两组病理分型及临床分期比较无统计差异，具有可比性。（表 2）

表 2　两组患者病理分型及临床分期比较

组别	例数	病理分型		临床分期	
		鳞癌	腺癌	IIIB 期	Ⅳ期
治疗组	30	17	13	6	24
对照组	30	16	14	7	23

注：病理分型：χ^2=0.067，*P*=0.795；临床分期：χ^2=0.098，*P*=0.754

1.3 两组患者肿瘤原发部位比较

肿瘤原发部位采用卡方检验，得χ^2=0.606，*P*=0.436，*P*＞0.05，具有可比性。（表 3）

表 3 两组患者肿瘤原发部位比较

组别	例数	中央型	周围型
治疗组	30	18	12
对照组	30	15	15

注：肿瘤原发部位：χ^2=0.606，*P*=0.436；

1.4 两组患者肿瘤转移数目比较

肿瘤转移数目比较采用卡方检验，得χ^2=0.842，*P*=0.657，*P*＞0.05，具有可比性。（表 4）

(C)1994-2022 China Academic Journal Electronic Publishing House. All rights reserved.　http://www.cnki.net

表 4 两组患者肿瘤转移数目比较

组别	例数	1 个转移灶	2 个转移灶	3 个及以上转移灶
治疗组	30	8	15	7
对照组	30	11	14	5

注： 肿瘤转移数目：χ^2=0.842，P=0.657；

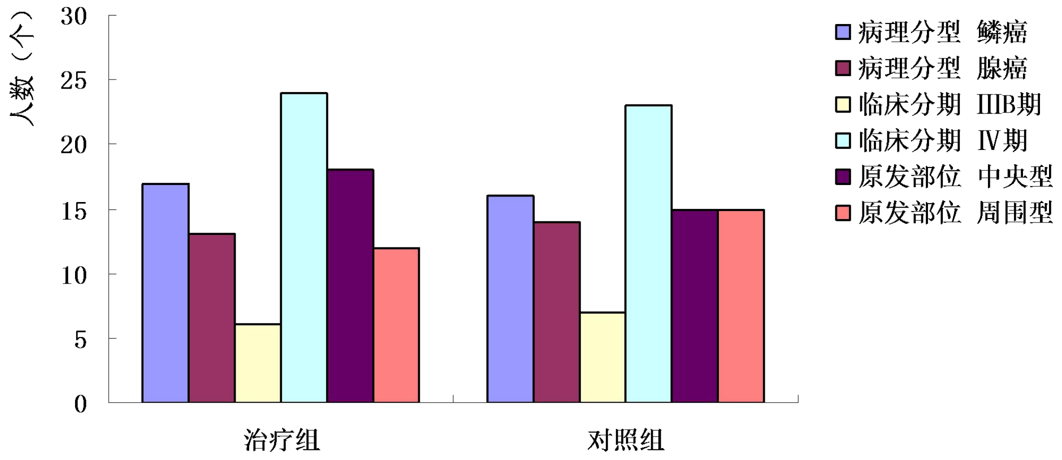

图 3 两组病理分型分期及原发部位比较

1.5 体力状况比较

治疗前 KPS 评分，用（ $\bar{\chi}$±s ）表示，比较采用 t 检验，得 t=1.046，P=0.300，P > 0.05，两组 KPS 评分基线平衡。（表 5）

表 5 两组患者 KPS 评分比较

组别	例数	Karnofsky 评分
治疗组	30	76.67±4.795
对照组	30	75.33±5.074

注： KPS 评分：t=1.046，P=0.300。

2 临床疗效结果

2.1 白细胞、血红蛋白、血小板

数据均采用（ $\bar{\chi}$±s ）表示，比较均采用 t 检验，比较两组白细胞得 t=1.107，P=0.273，比较两组血红蛋白得 t=1.030，P=0.307 比较两组血小板得 t=0.097，P=0.923。各组均 P > 0.05，具有可比性。治疗后经秩和检验，比较两组白细胞得 Z=3.590，P=0.001；比较两组血红蛋白得 Z=3.289，P=0.001；比较两组血小板得 Z=2.187，P=0.029；各组均 P

(C)1994-2022 China Academic Journal Electronic Publishing House. All rights reserved.   http://www.cnki.net

< 0.05，差异具有统计学意义。说明裴氏扶正抑瘤汤可以改善化疗引起的骨髓抑制。（表6、7）

表 6　治疗前两组患者白细胞、血红蛋白、血小板比较

组别	例数	WBC	HGB	PLT
治疗组	30	6.502±1.10	135.47±21.32	169.00±38.60
对照组	30	6.409±0.94	140.53±16.47	169.83±28.00

注：WBC：t=1.107，P=0.273 HGB: t=1.030，P=0.307 PLT: t=0.097，P=0.923

表7 治疗后两组患者白细胞、血红蛋白、血小板比较

指标	组别	0	I	II	III	IV
WBC	治疗组	18	9	3	0	0
	对照组	6	10	14	0	0
HGB	治疗组	19	10	1	0	0
	对照组	11	15	4	0	0
PLT	治疗组	16	13	1	0	0
	对照组	7	11	12	0	0

注：WBC: Z=3.590，P=0.001　HGB: Z=3.289，P=0.001　PLT: Z=2.187，P=0.029

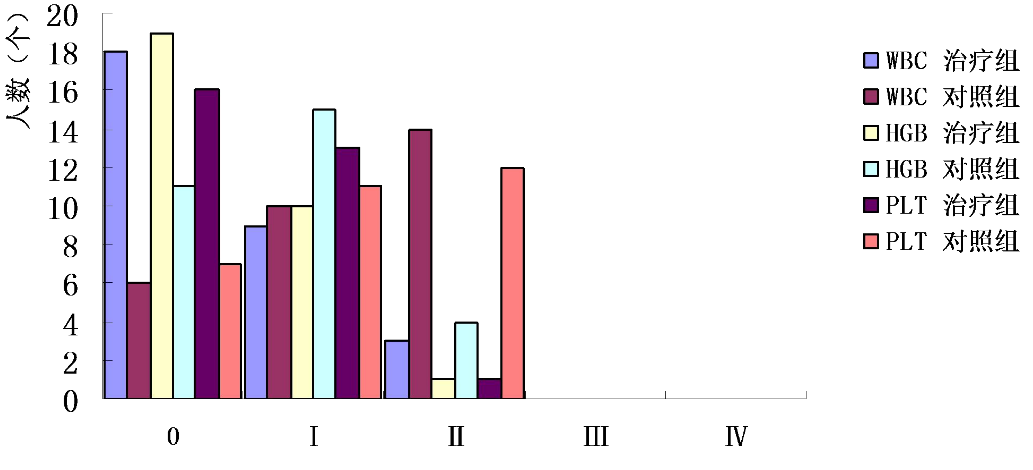

图 4治疗后两组患者白细胞、血红蛋白、血小板比较

2.2 中医症候积分

治疗前两组患者中医症候积分比较，采用t 检验得t=0.810，P=0.421，P > 0.05，差异无统计学意义。两组患者治疗后用秩和检验得Z=2.896，P=0.004，P < 0.05，两组中医症候积分治疗后差异有统计学意义。（表 8、9）

(C)1994-2022 China Academic Journal Electronic Publishing House. All rights reserved.　http://www.cnki.net

表 8　治疗前两组患者中医症候积分比较

组别	例数	中医症候积分
治疗组	30	13.63±1.974
对照组	30	13.23±1.851

注：中医症候积分：t=0.810，P=0.421

表 9 治疗后两组患者中医症候积分比较

组别	例数	显效	有效	无效
治疗组	30	16	12	2
对照组	30	7	13	10

注：中医证候积分：Z=2.896，P=0.004

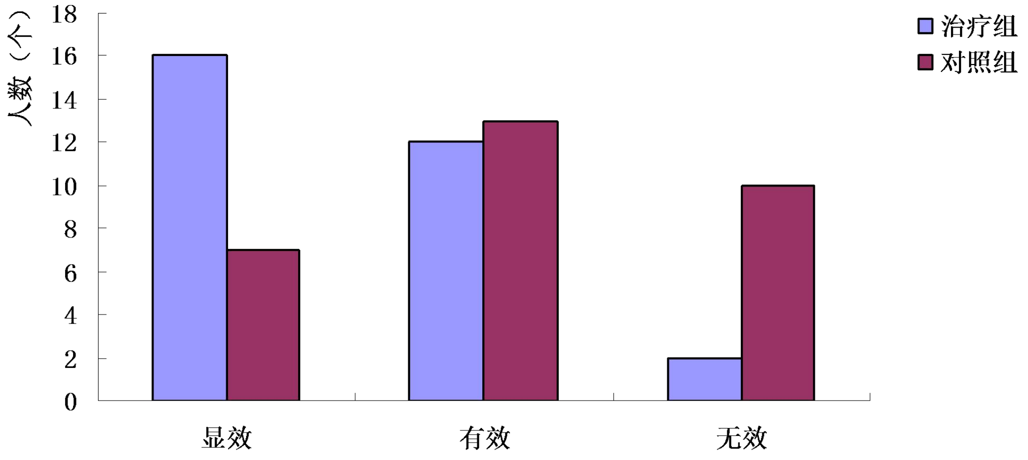

图 5 治疗后两组患者中医症候积分比较

2.3 Karnofsky 评分

治疗前后两组患者Karnofsky评分均采用t检验，治疗前比较t=0.673，P=0.527，P > 0.05，差异无统计学意义。治疗后组间比较t=5.873，P=0.001，P < 0.05，有统计学差异。治疗组前后比较：t=13.730，P < 0.001；　对照组前后比较：t=1.795，P=0.083。（表 10）

表10　两组治疗前后KPS评分比较

组别	例数	治疗前KPS评分	治疗后KPS评分
治疗组	30	76.67±4.795	85.33±5.074
对照组	30	75.33±5.074	81.00±7.120

注：治疗前组间比较：t=1.046，P=0.300；治疗后组间比较：t=2.715，P=0.009；治疗组前后比较：t=13.730，P<0.001；　对照组前后比较：t=1.795，P=0.083.

(C)1994-2022 China Academic Journal Electronic Publishing House. All rights reserved.　http://www.cnki.net

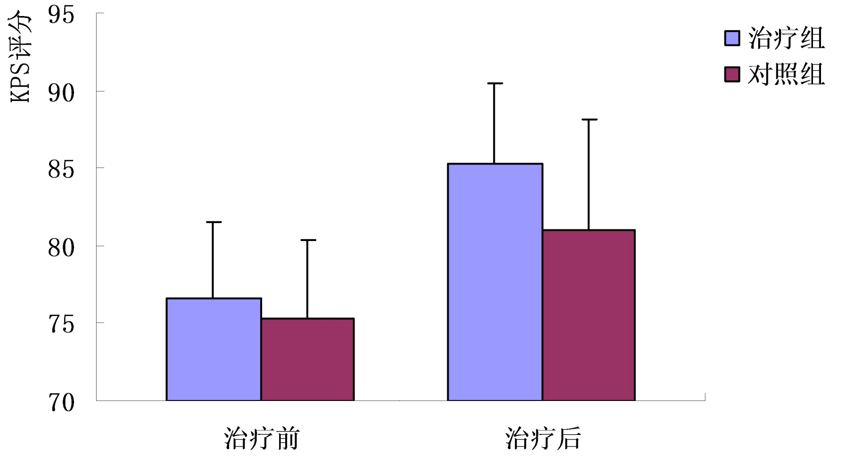

图6 两组治疗前后KPS评分比较

2.4 胃肠道反应

比较两组患者治疗后胃肠道反应情况，采用秩和检验得 Z=2.316，P=0.021，$P < 0.05$，两组差异有统计学意义。

表 11 两组患者胃肠道反应比较

指标	组别	0	I	II	III	IV
胃肠道反应	治疗组	10	14	4	2	0
	对照组	6	8	10	6	0

注：胃肠道反应：Z=2.316，P=0.021

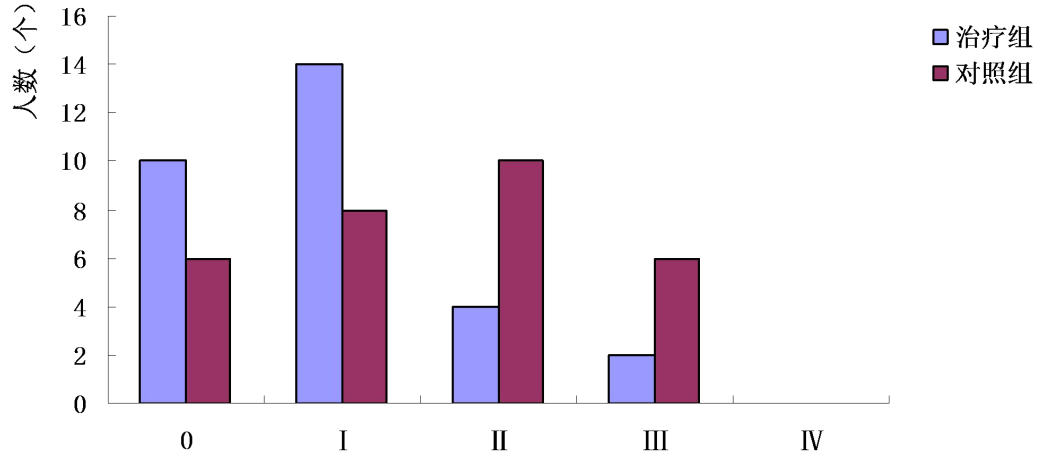

图 7 两组胃肠道反应比较

(C)1994-2022 China Academic Journal Electronic Publishing House. All rights reserved. http://www.cnki.net

2.5 患者生存质量

数据均采用（ $\bar{\chi}\pm s$ ）表示，比较均采用 t 检验。两组患者治疗前后生存质量总积分及身体状况、社会/家庭状况、与医生的关系、情绪状况、功能状况、症状/副作用等比较，无差异（P>0.05）；治疗组治疗后生存质量总积分、功能状况与对照组相比有差异，具有统计学意义（P＜0.05）；治疗组治疗后身体状况、情绪状况、症状/副作用与对照组相比差异显著，有统计学意义（P＜0.01）。治疗组治疗后社会/家庭状况、与医生的关系与对照组比较相比无差异（P＞0.05）。

表12　治疗组与对照组治疗前后生存质量总积分及各部分积分变化比较

组别	治疗组		对照组	
	治疗前	治疗后	治疗前	治疗后
身体状况	13.91±3.87	16.21±3.25**	13.84±3.95	9.88±3.92
社会/家庭状况	10.69±3.78	8.47±3.15	10.56±3.45	8.36±3.29
与医生的关系	5.38±1.43	6.59±1.47	5.41±1.28	6.62±1.69
情绪状况	9.37±2.73	11.98±2.85**	9.20±2.85	7.58±2.36
功能状况	13.42±3.49	16.32±3.60*	13.87±3.68	12.36±3.27
症状/副作用	19.52±4.13	21.07±4.59	19.39±4.61	26.18±4.88
总积分	72.29±8.91	82.36±8.54*	72.14±8.67	71.37±9.10

注：治疗后治疗组与对照组比较有差异，* $P < 0.05$，** $P < 0.01$

2.6 肺癌肿瘤标志物

两组治疗前后肿瘤标志物 CEA、CY21-1 比较均显示 P>0.05，差异无统计学意义。（表 13-14）

表 13 治疗前后 CEA 变化比较

组别	例数	治疗前CEA	治疗后CEA
治疗组	30	46.77±14.30	14.71±8.08
对照组	30	44.13±12.72	15.09±7.09

注：CEA：治疗前：t=0.755，P=0.453；治疗后：t=0.188，P=0.851

表 14 治疗前后 CY21-1 变化比较

组别	例数	治疗前CY21-1	治疗后CY21-1
治疗组	30	17.89±5.716	5.87±2.492
对照组	30	16.85±6.040	6.91±2.771

注：CY21-1：治疗前：t=0.685，P=0.496；治疗后：t=1.533，P=0.131

(C)1994-2022 China Academic Journal Electronic Publishing House. All rights reserved.　http://www.cnki.net

2.6 目标瘤体变化

比较两组患者治疗后目标瘤体的变化情况，采用秩和检验得 Z=1.290，*P*=0.197，*P* > 0.05，两组差异无统计学意义。（表 15）

表 15 目标瘤体变化比较

组别	例数	CR	PR	SD	PD
治疗组	30	0	13	11	6
对照组	30	0	9	11	10

注：目标瘤体变化：Z=1.290，*P*=0.197

3 安全性指标

两组患者治疗前后 ALT、AST、Cr、BUN 比较，均采用（ $\bar{\chi}$±s ）表示，治疗前后两组比较均示 *P* > 0.05，差异无统计学意义。（表 16-17）

表 16 ALT、AST 治疗前后比较

组别	ALT		AST	
	治疗前	治疗后	治疗前	治疗后
治疗组	25.93±6.469	28.40±6.195	26.13±6.606	31.53±6.442
对照组	28.00±6.597	30.77±6.089	27.40±6.279	32.10±5.827

注：ALT：治疗前：*t*=1.225，*P*=0.225；治疗后：*t*=1.429，*P*=0.141；AST:治疗前：*t*=0.761，*P*=0.450；治疗后：*t*=0.357，*P*=0.722

表 17 Cr、BUN 治疗前后比较

组别	Cr		BUN	
	治疗前	治疗后	治疗前	治疗后
治疗组	75.83±12.45	79.73±12.84	5.36±1.25	5.75±1.36
对照组	74.43±10.70	78.73±10.41	5.28±1.21	5.81±1.33

注：Cr：治疗前：*t*=0.467，*P*=0.642；治疗后：*t*=0.331，*P*=0.742；BUN:治疗前：*t*=0.263，*P*=0.794；治疗后：*t*=0.183，*P*=0.856

(C)1994-2022 China Academic Journal Electronic Publishing House. All rights reserved. http://www.cnki.net

讨论

原发性支气管肺癌在我国的发病呈上升态势[5]。在我国，环境污染、人口老龄化等问题已经成为助推肺癌发病的重要原因。其中多发于老年人，以 NSCLC 为[6]。目前，临床广泛采用的以手术、化放疗为主的治疗手段，并不适合于老年晚期 NSCLC 的治疗。摒弃以局部肿瘤的有无及大小评判疗效，转而以多学科综合治疗提高患者的生存质量成为当前管理老年晚期 NSCLC 的趋势。“人瘤共存”的理念，即使肺癌成为慢性病是临床的目标之一。

1 中医对老年晚期非小细胞肺癌认识

肺癌属于中医“肺积”、“ 肺花疮”、“ 肺痿”、“ 积聚”等范畴。肺癌的发生与外感邪气、饮食不调、情志不舒、久病体虚等相关，以痰、瘀、火、毒等病理产物的堆积为主，正气虚弱是其发病的基础。老年患者在机体功能、营养、合并症、心理、社会关系、药物的耐受性等方面有其特殊的病理生理特点，以手术、放化疗为主的手段在老年晚期肺癌的治疗上捉襟见肘。因此，中医药为主的综合治疗方式以其方法多样、疗效突出、低毒副作用等特点，在以提高老年晚期非小细胞肺癌患者的生活质量的目标中优势明显。

2 裴正学教授论治老年晚期非小细胞肺癌思想

2.1 正虚为老年晚期肺癌主要机制

裴正学教授认为该病的发病乃中医正虚发病学说的体现。“正气虚而后邪气踞之”，“正气虚则成岩”。因此，正气虚弱是老年晚期 NSCLC 的主要病理机制，在治疗过程中，扶正固本思想是需要贯穿始终的治疗大法。气血津液是人体重要的物质基础，正气虚弱不仅是物质基础的缺失，还有机体功能的低下。裴老认为正气是有先天之气和后天之气共同组成的，包括脾胃之气和肾气，即中气和元气，中气和吸入之清气居于胸中成为宗气，成为正气的重要组成部分[7]。中医扶正固本包括健脾益气、补气养血、温阳补肾、补益肝肾、益气养阴等丰富的内容。扶正固本方药存在着广泛的作用靶点，在减缓肿瘤的转移及复发、放化疗毒副反应，以及减少靶向药物的耐药等方面有着广泛应用。

2.2 扶正固本为大法

裴老在扶正固本的治法中强调健脾益肾，同时指出调肝是扶正固本的重要治法，调肝能够大大加强补脾益肾之功效。肝为刚脏，体阴用阳，肝主疏泄，具有调节人体气机的重要功能，肝藏血，有利于维持人体阴阳之平衡。脾为中土，喜燥恶湿，容易为湿所

(C)1994-2022 China Academic Journal Electronic Publishing House. All rights reserved. http://www.cnki.net

困，此所谓“脾胃为生痰之源”，需要肝脏的疏泄调达之功，脾胃方能运化水谷，上输于肺，灌溉四肢百骸。另外，“肝肾同源”，肝肾在生理病理上联系密切。肾主封藏，主骨生髓，精血互化，与肝的联系表现在精血同源、藏泄互用、阴阳互滋互制中。

2.3 炎瘤相长

裴老指出“炎症是肿瘤的好朋友”，肿瘤的发生和发展不仅与肿瘤本身的遗传学特异性相关，也与肿瘤所处微环境密切相关。流行病学证实，25%的肿瘤由炎症发展而来，炎症与肿瘤的发生有相关性[8]。有研究表明肿瘤与炎症的关系并非单向，一方面，通过外源性性途径，有微生物感染或无菌性炎症环境，可以促发基因突变，形成肿瘤。另一方面，人们在炎症细胞和调节因子被发现大量存在于肿瘤微环境中，这就是内源性途径，即肿瘤促进了炎症微环境的形成[9]。肿瘤的炎症微环境的形成与肿瘤的免疫逃逸机制密切相关。由于组织损伤炎性通路（NF-κB 信号通路、信号转导和转录激活因子3(STAT3) 、缺氧诱导因子(HIF))的激活,炎症的损伤和修复的持续进行加强了炎症免疫细胞（ TAMs、MDSCs、Treg）和促炎因子子 CXC4、CCL-2、肿瘤坏死因子α(TNF-α) 、IL-6、IL-2、IL-1 等在肿瘤部位的募集和释放，机体的修复和灭火机制的重塑导致了炎症免疫细胞免疫表型的转化和修饰，使这些炎症免疫细胞不但不能帮助免疫系统消灭肿瘤细胞，反而促进肿瘤的发生发展，其所分泌的免疫抑制因子如 IL-10、TGF-β、血管内皮生长因子(VEGF) 等在损伤部位的聚集，促进肿瘤的免疫逃逸、生长和转移。裴老指出以上诸多免疫表型特征的核心是正气虚弱的表现，炎症微环境是属于中医“邪毒”的范畴，治疗上需要扶正固本配合清热解毒，即固本清源之法。

2.4 辨证治本

裴老提出“辨证论治就是治本”，“ 围点打援”是治疗晚期恶性肿瘤的战略思想，为晚期肿瘤的治疗开辟了新的治疗思路。裴老指出，在晚期肿瘤的治疗中，临床的目标并不能仅仅着眼于局部，肿瘤的客观缓解可能是困难的。因此，治疗目标应该是关注患者的感受，努力改善患者的各种不适症状，以提高患者的生存质量为目标。裴老创造性的引用解放战争时的战略战术思想，形象的说明了这一治疗晚期肿瘤的思想。

3 方药分析

3.1 裴氏扶正抑瘤汤组成

裴氏扶正抑瘤汤由太子参、北沙参、潞党参、人参须、生地、山萸肉、黄芪、丹参、当归、白芍、女贞子、墨旱莲、白花蛇舌草、龙葵、半枝莲组成。

(C)1994-2022 China Academic Journal Electronic Publishing House. All rights reserved. http://www.cnki.net

3.2 裴氏扶正抑瘤汤解析

3.2.1 单味药药理研究

（1）北沙参为伞形科植物珊瑚菜的干燥根，广泛分布于我国东北和东南部省份，为常用中药，其性凉，味甘淡，归经属于肺、脾、肝、心，具有清肺润燥，益气养阴，补脾生津等功效。李建业等[10]证实北沙参提取物可以增加 BALB /c 小鼠脾脏重量，使腹腔巨噬细胞、淋巴细胞、NK 细胞的作用加强。杨宪勇[11]证实北沙参是通过增加淋巴细胞数量和 T 淋巴细胞亚群来增强免疫功能，可以使 CTX 免疫抑制模型小鼠外周血中的 $CD8^+$、$CD4^+$ 及 $CD3^+$的数量增加，Ts/Th 比值下降。

（2）太子参是石竹子科植物孩儿参之干燥块根，性平，味甘、微苦，归经属肺、脾经，有健脾益肺，养阴生津之功效。太子参含有多种微量元素、氨基酸、糖类、核苷酸、脂肪酸、磷脂类、环肽类等多种成分。具有应激抗疲劳，增强免疫功能[14]，抗氧化[15]，降糖降脂改善心肌梗死所致心衰等作用。太子参有许多内生真菌，其产生的许多次生代谢产物具有很好的抗肿瘤作用，蔡巧燕[16]等用 MTT 法检测分离太子参内生真菌 18 株，其中 6 株对肿瘤具有明显的抑制作用，3 株具有较好的抗氧化活性。

（3）人参须是五加科物人参的干燥根，是非常贵重的中药材。性温，味甘，微苦，《神农本草经》谓：人参能补五脏、安精神、定魂魄。《本经逢源》指出：人参补肺益气，使肺气旺盛，四脏之气皆受其裨，而精生形盛。由于人参的主要成分是人参皂苷，人参皂苷主要存在于人参的周皮与皮层之中，而肉质根茎含量相对较少，实验表明人参主根皂苷的含量在白参和红参中分别是 2.1%和 2.5%，而根须中分别是 9.52%和 9.74%[17]。人参具有促进造血、减缓放疗引起骨髓抑制、增强免疫、抑制肿瘤、抗疲劳、改善循环等广泛作用。尹利明等[18]证实人参总皂苷可以诱导 MSCs 向成骨细胞分化，从而促进骨髓造血。黄颖等[19]研究发现电离辐射能够导致造血干细胞内大量活性氧的释放，从而改变细胞微代谢环境，导致细胞发生氧化应激反应，而人参皂苷具有抵抗电离辐射所致造血干细胞的凋亡，减少细胞内 caspase-3，升高 Nrf-2 等多种途径来减缓这类氧化应激的发生。柯仕忠等[20]发现人参皂甙 Rg3 相比顺铂对 LLC 荷瘤小鼠肺癌具有更好的抑瘤作用，同时能够诱导肿瘤抗原特异性 CTL 和 $CD4^+$T 细胞、$CD8^+$T 细胞的增加，增加胸腺、脾脏的质量，而 LLC 荷瘤小鼠肺癌明显的免疫抑制是疗效显著的机制。

（4）潞党参为桔梗科植物党参的根。上党是党参的道地产区，“参以上党者佳”。其味甘性平，具有健脾滋胃，养血润肺之效，功殊于人参。吴君等[21]证实潞党参能够增加胸腺、脾脏的重量，对抗戊巴比妥钠的中枢抑制作用。胡建燃等[22]证实，党参多糖具有

(C)1994-2022 China Academic Journal Electronic Publishing House. All rights reserved. http://www.cnki.net

很好的抗氧化活性。且对多种肿瘤细胞有较强的抗肿瘤药理作用，其对肝癌细胞SMMC-7721有很明显的抑制作用[23]。对于免疫系统的调节，体现在使CTX所致免疫抑制模型小鼠的胸腺、脾脏指数升高，亦可对抗由CTX对遗传的诱变作用[24]。

（5）黄芪是豆科植物蒙古黄芪或膜荚黄芪的根。其性温味甘，具有补益中气，固表，托毒生肌等功效。黄素有“补药之长”之称，其所含有的黄芪多糖、三萜、黄酮等成分均有非常活跃的调节免疫、抗肿瘤、延缓衰老、抗病毒等作用。黄芪多糖在调节免疫方面作用非常多样，不仅能够激活B细胞和巨噬细胞的增生，且能够促进T细胞的增生[25]。研究显示黄芪多糖在器官免疫[26]、细胞免疫[27]、抗病毒免疫及相关基因调控方面作用突出。对肿瘤免疫主要体现在其通过调节Toll样受体（TLR），T细胞亚群，IL, IL，TNF-α， IFN-γ 及VEGF等发挥作用。武有明等[28]发现黄芪多糖能够使肺癌微环境肿中的骨髓间充质干细胞（BMSCs）的形态、增殖特性异常改变受到抑制。

（6）女贞子是木犀科植物女贞的干燥成熟果实，其性凉味甘，微苦，具有补益肝肾、乌发明目、强健腰膝等功效。女贞子主要含有萜类、黄酮类、挥发油类、糖类等成分，现代药理学表明其在调节免疫、抗炎、抗肿瘤、保肝等方面作用显著。邹慧等[29]给ICR小鼠用女贞子粗多糖灌胃14d，发现小鼠免疫器官胸腺和脾脏指数明显增加，能够显著的促进淋巴细胞的增生，血清IL-2、IL-4、IFN-γ的水平较对照组明显升高[30]。此外，女贞子还有很好的抗诱变、抗肿瘤功效。对包括肺癌、消化道癌症等多种肿瘤具有很好的抑制作用[31]。

（7）旱莲草是菊科鳢肠属植物鳢肠的干燥地上部分，其性寒味甘、酸，归经属肝、肾经。三萜皂苷类、黄酮类、噻吩类及香豆草醚类等活性成分是旱莲草的主要成分。现代药理学研究表明，旱莲草具有抑制炎症、肿瘤、抗诱变、调节免疫系统、保护肝肾脏、抗氧化与衰老等作用[32]。许小华等[33]应用旱莲草提取物旱莲草多糖的不同剂量组对正常小鼠腹腔巨噬细胞的吞噬功能的影响，且以小剂量组效果更为明显。庄晓燕等[34]用旱莲草水煎剂干预分别由CTX和氢化可的松所致小鼠胸腺凋亡模型，证实旱莲草水煎剂可以明显的抑制两种药物所致的小鼠胸腺细胞的凋亡，而对正常胸腺细胞无不良影响。丁淑敏[35]发现旱莲草提取物有较强的抑制肺癌细胞，促进肺癌细胞凋亡的作用。实验发现旱莲草提取物对A549细胞有一定抑制作用，其浓度相当于生药1000ug/ml时对该细胞24h凋亡率可达到47.55%，促凋亡蛋白Bax、cleaved-caspase8、cleaved-caspase9、cleaved-caspase3的表达均有增高。

（8）半枝莲为唇形科植物半枝莲的全草，其性寒味辛、苦，归经属于肺、肝、肾

(C)1994-2022 China Academic Journal Electronic Publishing House. All rights reserved. http://www.cnki.net

经。具有清热解毒，活血化瘀，利尿，抗肿瘤，止血之功效。研究表明半枝莲含有包括多糖、黄酮类、生物碱、萜类、挥发油等多种化学成分，有很好的抗肿瘤、抗菌、抗氧化等活性[36]。李玉凤[37]等证实半枝莲多糖能够抑制 Lewis 肺癌小鼠肿瘤细胞的增殖，相对于 CTX 组，半枝莲多糖具有更明显的抑瘤率，免疫器官指标明显升高，抑制 VEGF、EGFR、nm23 蛋白的表达是其作用机制。张梅等[38]发现半枝莲 70%乙醇水溶液提取物能够显著的促进人肺癌 A549 细胞的凋亡，测得细胞凋亡相关基因 Survivin、Caspase-3 高表达，可以诱导小鼠脾脏分泌免疫因子 TNF-α和 IL-2,使小鼠免疫功能提高，证实半枝莲在体外和小鼠体内都有较好的抑瘤效果。

（9）白花蛇舌草是茜草科耳草属植物白花蛇舌草的全草，其性凉味甘、淡，归经属于胃、大肠、小肠经，具有清热解毒，利尿，活血止痛，消痈之功效。萜类、多糖、蒽醌类、黄酮类、甾醇、环烯醚萜等是白花蛇舌草含有的主要化学成分[39]。研究表明白花蛇舌草在抗癌、抗氧化、抗炎方面疗效突出，其抗癌作用明显且广泛，在包括呼吸系统、消化系统、生殖系统、白血病、骨髓瘤等方面均有着明显的疗效[40]。赵诗云等[41]用 CTX、CTX 加复方白花蛇舌草和复方白花蛇舌草组干预荷 Lewis 小鼠，经过 13 天，免疫器官方面，CTX 及 CTX 加复方白花蛇舌草组小鼠胸腺质量均下降，复方白花蛇舌草组胸腺重量增加。瞿俊勇等[42]探讨了白花蛇舌草多糖对免疫抑制小鼠的调节作用，实验表明，白花蛇舌草能有显著的增加由环磷酰胺所致免疫抑制小鼠模型的脾脏、胸腺系数，血清溶血素、脾淋巴细胞增殖能力加强，脾脏 NK 细胞及血清 IL-2、IL-6、TNF-α 水平均显著升高，证实白花蛇舌草在调节免疫抑制小鼠的免疫功能方面疗效卓著。

（10）龙葵为茄科茄属植物龙葵的全草，其性寒味苦，具有清热解毒，利水消肿的功效，其主要含有甾体类、有机酸类、木脂素类等有效成分，有较好的抗炎、抗肿瘤作用。李丽等[43]龙葵 80%乙醇浸膏能够显著增强 Lewis 肺癌小鼠的免疫功能，减少转移的发生。陈来等[44]实验了从龙葵中提取的澳洲茄碱对肺癌的作用，证实澳洲茄碱对人类肺癌 A549 和小鼠 Lewis 肺癌细胞 LLC 细胞株均有很好的抑制作用，并探讨了其可能的机制是诱导细胞凋亡或者细胞坏死为主。

以上药物现代药理学研究表明，北沙参、太子参、人参须、潞党参、黄芪、女贞子、旱莲草、半枝莲、龙葵、白花蛇舌草均具有很好的增强肺癌患者免疫功能、抗肿瘤、抗炎、抗氧化等作用，在免疫调节方面以北沙参、太子参、人参须、潞党参、黄芪为长，半枝莲、龙葵、白花蛇舌草三者具有比较好的抑制肿瘤的侵袭性及转移的作用，为本方在临床治疗肺癌取得良好的疗效提供了支持依据。

(C)1994-2022 China Academic Journal Electronic Publishing House. All rights reserved. http://www.cnki.net

3.2.2 裴氏扶正抑瘤汤方解

裴氏扶正抑瘤汤为裴老长期临床经验用方，该方以健脾，补益肝肾为主，兼有凉血解毒，活血化瘀之功。其具体组成为：人参须、生地黄、黄芪、当归、丹参、白芍、山萸肉、女贞子、北沙参、旱莲草、白花蛇舌草、太子参、半枝莲、潞党参、龙葵。方中潞党参、太子参、人参须、北沙参补脾益气，培土生金。《素问·经脉别论篇》“饮入于胃……揆度以为常也”之论完整的论述了饮食水谷经脾胃及肺输布四肢百骸的过程，气血乃水谷精微变化而来，此补脾既能益肺之理。黄芪、丹参、当归、白芍是裴老补肝养肝之药组，肝为东方青龙，脏居人体之右而其气左升，肺肃杀之气从右降，为人体调节气机之两轮，脾胃斡旋于中，且乙癸同源，木能疏土，此肝、肾、肺、脾之机明矣。肝为风火之脏，体阴而用阳，肝主藏血，调血养血即所谓补肝养肝也。柯琴曰：“经云：心生血，肝藏血。故凡生血者，则究之与心；调血者，当求之于肝也。”当归，白芍取自四物汤，此方乃肝经调血之专剂，非心经生血之主方。黄芪补气升阳，《医宗金鉴·删补名医方论》曰：昔人有言：见血休治血，必先调其气。又云：四物汤不得补气药，不能成阳生阴长之功。丹参为活血、调血之药，四药相配伍，而补肝养肝之效备矣。生地黄、山茱萸取自六味地黄汤之三补，取补肾填精生髓之意；女贞子补肾滋阴，旱莲草养肝益肾，两药相合增强补益肝肾之功效；丹参合白花蛇舌草、半枝莲、龙葵共奏凉血解毒、活血化瘀之功。诸药并用，共奏补肝肾填精生髓，健脾益气，凉血解毒，活血化瘀之效。

4 研究结果分析

4.1 中医临床症候及体力状况

治疗前两组患者中医症候积分比较，采用 t 检验得 t=0.810，P=0.421，P > 0.05，差异无统计学意义。两组患者治疗后用秩和检验得 Z=2.896，P=0.004，P < 0.05，两组中医症候积分治疗后差异有统计学意义。治疗前后两组患者 Karnofsky 评分均采用 t 检验，治疗前比较 t=0.673，P=0.527，P > 0.05，差异无统计学意义。治疗后组间比较 t=5.873，P=0.001，P < 0.05，有统计学差异。治疗组前后比较：t=13.730，P < 0.001； 对照组前后比较：t=1.795，P=0.083。表明裴氏扶正抑瘤汤在随证加减后，能够在改善患者的临床症状方面取得较好的效果。患者咳嗽、咳血、胸痛、发热多因为肺气阴两虚，热毒内盛所致，裴氏扶正抑瘤汤包含养阴生津，清热解毒之药。患者乏力症状改善明显，裴氏扶正抑瘤汤以益气健脾，养肝补肾见长，故能很好的改善患者乏力的症状。

(C)1994-2022 China Academic Journal Electronic Publishing House. All rights reserved. http://www.cnki.net

4.2 白细胞、血红蛋白、血小板

数据均采用（ $\bar{\chi}$±s ）表示，比较均采用 *t* 检验，比较两组白细胞得 t=1.107，P=0.273，比较两组血红蛋白得 t=1.030，P=0.307 比较两组血小板得 t=0.097，P=0.923。各组均 $P > 0.05$，具有可比性。治疗后经秩和检验，比较两组白细胞得 Z=3.590，P=0.001；比较两组血红蛋白得 Z=3.289，P=0.001；比较两组血小板得 Z=2.187，P=0.029；各组均 $P < 0.05$，差异具有统计学意义。说明裴氏扶正抑瘤汤能够较好的缓解有化疗所致的骨髓抑制，方中人参、黄芪、潞党参、太子参等养阴益气类中药能够很好的上调骨髓细胞钙敏感受体（CaSR），调节巨噬细胞集落刺激因子（GM-CSF）、粒细胞集落刺激因子（G-CSF）等因子。山茱萸、生地、墨旱莲、女贞子等补肾中药能够很好的促进骨髓造血干细胞进入细胞周期，解除细胞周期阻滞，进行增殖。

4.3 胃肠道反应

比较两组患者治疗后胃肠道反应情况，采用秩和检验得 Z=2.316，P=0.021，$P < 0.05$，两组差异有统计学意义。提示裴氏扶正抑瘤汤能够有效缓解化疗所致胃肠道反应。

4.4 患者生存质量

两组患者治疗前后生存质量总积分及身体状况、社会/家庭状况、与医生的关系、情绪状况、功能状况、症状/副作用等比较，无差异（$P>0.05$）；治疗组治疗后生存质量总积分、功能状况与对照组相比有差异，具有统计学意义（$P < 0.05$）；治疗组治疗后身体状况、情绪状况、症状/副作用与对照组相比差异显著，有统计学意义（$P < 0.01$）。治疗组治疗后社会/家庭状况、与医生的关系与对照组比较相比无差异（$P > 0.05$）。说明裴氏扶正抑瘤汤能够较好的改善或者的生存质量。

综上，裴氏扶正抑瘤汤在老年晚期非小细胞肺癌的治疗中，在肿瘤的近期客观缓解率方面疗效不甚理想，可以较为满意的改善患者中医临床症状积分和体力状况，在减轻患者因化疗引起的胃肠道反应和骨髓抑制方面效果明显。无明显的毒副反应的发生，安全性良好，值得临床推广和进一步的研究。

(C)1994-2022 China Academic Journal Electronic Publishing House. All rights reserved. http://www.cnki.net

结语

1 结论

1.1裴氏扶正抑瘤汤可以改善化疗引起的胃肠道反应及骨髓抑制等严重的不良反应，使患者能够较好的耐受化疗。

1.2在改善患者中医临床症状和体力状况方面效果突出，安全性良好。

2 不足及展望

（1）本试验样本量较小，且未采用盲法可能造成试验数据的偏倚。

（2）试验周期较短，仅随访3月，缺乏对患者较长期生存期和生存质量的评价。

（3）由于试验经费的限制，未将免疫指标纳入。

（4）在条件成熟时，可望在本次试验的基础上加大样本量，采用盲法，延长观察时间，纳入更多指标，以期对裴氏扶正抑瘤汤的近期和较长期的作用进行更好的评价。

(C)1994-2022 China Academic Journal Electronic Publishing House. All rights reserved.   http://www.cnki.net

参考文献

[1]Ferlay J, Soerjomataram I, Dikshit R, et al. Cancer incidence and mortality worldwide: sources, methods and major patterns in GLOBOCAN 2012.[J]. International Journal of Cancer, 2015, 136(5):E359.

[2]支修益,石远凯,于金明.中国原发性肺癌诊疗规范(2015 年版)[J].中华肿瘤杂志，2015，37(1):67-78.

[3]Lee P N, Forey B A, Coombs K J. Systematic review with meta-analysis of the epidemiological evidence in the 1900s relating smoking to lung cancer[J]. BMC Cancer, 2012, 12(1):385.

[4]郑筱萸.中药新药临床研究指导原则:试行[M].2002.

[5]Jemal A, Bray F, Center M M, et al. Global cancer statistics[J]. Ca A Cancer Journal for Clinicians, 2015, 65(2):87.

[6]Ferlay J. Global cancer statistics, 2002.[J]. Ca A Cancer Journal for Clinicians, 2005, 55(2):74–108.

[7]裴正学,冯小荣,何红珍等.扶正固本与免疫[J].中医临床研究,2015,7(30):4-6.

[8]Hussain S P, Harris C C. Hussain SP, Harris CCInflammation and cancer: an ancient link with novel potentials. Int J Cancer 121(11): 2373-2380[J]. 2007, 121(11):2373-2380.

[9]吉登波,崔景荣.肿瘤的发生、发展及治疗与炎症的关系[J].中国新药杂志,2010(17) : 1551-1555.

[10]李建业，刘运周，张薇等.北沙参对小鼠免疫功能的影响研究[J].中国实验诊断学,2012(9):1599-1601.

[11]杨宪勇.北沙参对免疫抑制 C57BL/6J 小鼠 T 淋巴细胞亚群影响的实验研究[J].泰山医学院学报,2012, 33(4):247-249.

[12]Kenney D M, Geschwindt R D, Kary M R, et al. Detection of Newly Diagnosed Bladder Cancer, Bladder Cancer Recurrence and Bladder Cancer in Patients with Hematuria Using Quantitative RT-PCR of Urinary Survivin[J]. Tumor Biology, 2007, 28(2):57.

[14]Cai J, Xiao-Dong L I, Chen X Z. The Immune Effects of Crude Extract of Pseudostellaria Polysaccharide in Mice[J]. Journal of Fujian College of Traditional Chinese Medicine, 2005.

[15]Chen Z, Li S, Wang X, et al. Protective effects of Radix Pseudostellariae polysaccharides against exercise-induced oxidative stress in male rats.[J]. Experimental & Therapeutic Medicine, 2013, 5(4):1089-1092.

(C)1994-2022 China Academic Journal Electronic Publishing House. All rights reserved. http://www.cnki.net

[16]蔡巧燕,林珊,肖桂清等.太子参内生真菌体外抗肿瘤、抗氧化活性研究[J].福建中医药大学学报,2011,21(6):41-43.

[17]逄世峰,李亚丽,许世泉等.人参不同部位人参皂苷类成分研究[J].人参研究,2015, 27(1):5-8.

[18]尹利明,赵燕娜,杜文喜等.人参总皂苷增强成骨分化的间充质干细胞促造血作用研究[J].中国药理学通报,2015,v.31(1):45-49.

[19]黄颖,梁晓燕,李骋进等.人参皂苷对不同强度电离辐射损害人造血干细胞的缓解作用[J].中国组织工程研究,2015,19(1):124-129.

[20]柯仕忠,刘瑶,金浩杰等.人参皂甙 Rg3 抗小鼠 Lewis 肺癌的机制研究[J].免疫学杂志,2012(5):389-393.

[21]吴君,黄萍,成金乐等.党参破壁粉粒增强免疫功能及抗疲劳作用的研究[J].中国实验方剂学杂志, 2011, 17(4):179-181.

[22]胡建燃,郭阳,李平.潞党参多糖的提取及其抗氧化活性分析[J].中国食品添加剂, 2016(7):93-96.

[23]武静莲,徐强,谢亲建等.党参抗肿瘤药理作用研究[J]. 西部中医药,2016,29(8):18-21.

[24]余兰,王毅.道真洛龙党参多糖对小鼠免疫活性的影响[J].遵义医学院学报,2016, 39(1):10-13.

[25] 林梦感，杨义芳,许海燕.黄芪多糖 MAPS-5 的化学结构及其体外淋巴细胞增殖活性[J]. 中草药,2009,40(12):1865-1868.

[26]Chen R Z, Tan L, Jin C G, et al. Extraction, isolation, characterization and antioxidant activity of polysaccharides from Astragalus membranaceus[J]. Industrial Crops & Products, 2015, 77(19):434-443.

[27]Chen W, Li Y M, Yu M H. Astragalus polysaccharides inhibited diabetic cardiomyopathy in hamsters depending on suppression of heart chymase activation.[J]. Journal of Diabetes & Its Complications, 2010, 24(3):199-208.

[28]武有明,张齐,刘永琦等.黄芪多糖对肺癌微环境中 BMSCs 增殖及 TAFs 分化的影响[J].中药药理与临床,2015(6):76-79.

[29]邹慧,劳雪芬,王程凯等.富硒女贞子粗多糖对小鼠免疫功能的影响[J].中国兽医学报,2015,35(2):283-286.

[30]Ju H Y, Chen S C, Wu K J, et al. Antioxidant phenolic profile from ethyl acetate fraction of Fructus Ligustri Lucidi with protection against hydrogen peroxide-induced oxidative damage in SH-SY5Y cells[J]. Food & Chemical Toxicology, 2012, 50(3–4):492-502.

(C)1994-2022 China Academic Journal Electronic Publishing House. All rights reserved. http://www.cnki.net

[31]刘亭亭，王萌．女贞子化学成分与药理作用研究进展[J]．中国实验方剂学杂志,2014,20(14):228-234.

[32]施嫣嫣,张丽,丁安伟.墨旱莲化学成分及药理作用研究[J].吉林中医药,2011, 31 (1):68-70.

[33]许小华,郝鹏飞,杨云等.墨旱莲多糖对正常小鼠免疫功能的实验研究[J].中国实验方剂学杂志,2010,16(5):181-182.

[34]庄晓燕，杨菁，王怡薇等．墨旱莲对胸腺细胞凋亡影响的研究[J]．数理医药学杂志,2010,23(2):228-230.

[35]丁淑敏.墨旱莲防治肺癌物质基础组分结构研究[D].南京中医药大学,2014.

[36]林靖怡，刘韶松，明艳林．半枝莲化学成分及药理活性研究进展(综述)[J]．亚热带植物科学,2015(1):77-82.

[37]李玉凤,李静,王晴等.半枝莲多糖对 Lewis 肺癌小鼠 VEGF、EGFR、nm23 表达的影响[J]. 光明中医,2017,32(1).

[38]张梅,韩松洋,葛圆圆等.半枝莲提取物对人肺癌 A549 细胞的凋亡作用及其对凋亡相关基因表达的影响[J].陕西中医,2016,37(6):756-758.

[39]刘艳群,殷文杰,刘勇等.白花蛇舌草化学成分研究进展[J].山东中医杂志, 2014(8).

[40]纪宝玉,范崇庆,裴莉昕等.白花蛇舌草的化学成分及药理作用研究进展[J].中国实验方剂学杂志,2014,20(19):235-240.

[41]赵诗云,张瑾楠,吴东风等.复方白花蛇舌草治疗肺癌荷瘤小鼠的实验研究[J].实用中西医结合临床,2014,14(9):81-83.

[42]瞿俊勇，田梦，贺建华等．白花蛇舌草多糖对免疫抑制小鼠的免疫调节作用研究[J]．中药材,2015(9):1942-1945.

[43]李丽，邹明雷．龙葵 80%乙醇浸膏对 Lewis 肺癌小鼠侵袭转移的影响[J]．临床医学， 2015(3):114-115.

[44]陈来，李姗姗，金德忠等．中药龙葵提取物澳洲茄碱对肺癌细胞抑制作用[J]．时珍国医国药,2015(2):333-334.

(C)1994-2022 China Academic Journal Electronic Publishing House. All rights reserved. http://www.cnki.net

附表

附表 1　　原发性肺癌的分期标准

NSCLC 的 TNM 分期采用国际肺癌研究协会(International Association for the Study of Lung Cancer，IASLC)2009 年第七版分期标准(IASLC 2009)。

TNM 分期符号说明

TNM 分期	符号说明
T-原发病灶	Tx: 原发肿瘤不能评价；或痰、支气管冲洗液找到癌细胞，但影像学或支气管镜没有可视肿瘤。<br>T0:无原发肿瘤证据<br>T1: 肿瘤最大径≤3 cm，周围被肺或脏层胸膜所包绕，支气管镜下肿瘤侵犯没有超出叶支气管近端以上位置（即没有累及主支气管）<br>T1a 肿瘤最大径≤2 cm<br>T1b 肿瘤最大径>2 cm 但≤3 cm。<br>T2: 肿瘤>3 cm 但≤7 cm 或者肿瘤具有以下任一特征：<br>累及主支气管，但距隆突≥2 cm<br>侵犯脏层胸膜<br>伴有扩展到肺门的肺不张或阻塞性肺炎，但未累及全肺<br>T2a 肿瘤最大径>3 cm 但≤5 cm<br>T2b 肿瘤最大径>5 cm 但≤7 cm<br>T3: 肿瘤>7 cm 或肿瘤已直接侵犯了下述结构之一者：胸壁（包括肺上沟瘤）、膈肌、膈神经、纵隔胸膜、心包壁层；或肿瘤位于距隆突 2 cm 以内的主支气管，但尚未累及隆突；或伴有累及全肺的肺不张或阻塞性肺炎或原发肿瘤同一叶内

(C)1994-2022 China Academic Journal Electronic Publishing House. All rights reserved.　http://www.cnki.net

	出现分散的单个或多个卫星结节。<br>T4: 任何大小的肿瘤已直接侵犯了下述结构之一者：纵隔、心脏、大血管、气管、喉返神经、食管、椎体、隆突；同侧非原发肿瘤所在叶的其他肺叶出现分散的单个或多个肿瘤结节
N-区域淋巴结	Nx:区域内淋巴结不能评价<br>N0:无淋巴结转移<br>N1: 转移至同侧支气管旁淋巴结和/或同侧肺门淋巴结，和肺内淋巴结，包括直接侵犯<br>N2 转移至同侧纵隔和/或隆突下淋巴结<br>N3 转移至对侧纵隔淋巴结、对侧肺门淋巴结、同侧或对侧斜角肌或锁骨上淋巴结
M-远处转移	Mx:远处转移不能测定<br>M0:无远处转移<br>M1:有远处转移<br>M1a：对侧肺叶出现分散的单个或多个瘤结节；胸膜结节或恶性胸腔（或心包）积液<br>M1b：远处转移

(C)1994-2022 China Academic Journal Electronic Publishing House. All rights reserved.   http://www.cnki.net

附表 2　　肺癌分期标准

分期		TNM
隐性肺癌		TxN0M0
原位癌 0 期		TisN0M0
Ⅰ期	ⅠA 期	T1a、bN0M0
	ⅠB 期	T2aN0M0
Ⅱ期	ⅡA 期	T2bN0M0
		T1a、bN1M0
		T2aN1M0
	ⅡB 期	T2bN1M0
		T3N0M0
Ⅲ期	ⅢA 期	T3N1M0
		T1a、bN2M0
		T2a、bN2M0
		T3N2M0
		T4N0M0
		T4N1M0
	ⅢB 期	T4N2M0
		T4N3M0
		任何 T，N3，M0
Ⅳ期		任何 T，任何 N，M1a、b

(C)1994-2022 China Academic Journal Electronic Publishing House. All rights reserved. http://www.cnki.net

附表 3　　WHO 制定的《抗癌药物急性与亚急性毒副反应的表现及分度标准》

器官或系统	0 度	Ⅰ度	Ⅱ度	Ⅲ度	Ⅳ度
血液系统					
血红蛋白（g/L）	≥110	95～109	80～94	65～79	<65
白细胞（$\times 10^9$/L）	≥4.0	3.0～3.9	2.0～2.9	1.0～1.9	<1.0
粒细胞（$\times 10^9$/L）	≥2.0	1.5～1.9	1.0～1.4	0.5～0.9	<0.5
血小板（$\times 10^9$/L）	≥100	75～99	50～74	25～49	<25
出血	无	瘀点	轻度出血	严重出血	导致衰竭
胃肠道					
胆红素	≤1.25×N	1.26～2.50×N	2.6～5.0×N	5.1～10.0×N	>10×N※
谷丙转氨酶	≤1.25×N	1.26～2.50×N	2.6～5.0×N	5.1～10.0×N	>10×N
碱性磷酸酶	≤1.25×N	1.26～2.50×N	2.6～5.0×N	5.1～10.0×N	>10×N
口腔	无异常	红斑，疼痛	红斑，溃疡，可进食	溃疡，只能进流食	不能进食
恶心呕吐	无	恶心	暂时性呕吐	呕吐，需治疗	难控制的呕吐
腹泻	无	短暂（<2 天）	能忍受(>2 天)	不能忍受，需治疗	血性腹泻
肾、膀胱					
尿素氮 血尿素	≤1.25×N	1.26～2.50×N	2.6～5.0×N	5.1～10.0×N	>10×N
肌　酐	≤1.25×N	1.26～2.50×N	2.6～5.0×N	5.1～10.0×N	>10×N
蛋白尿	无	+，<0.3 克 /100 毫升	++，+++，.3˜1.0/100 毫升	++++，>1.0 克 /100 毫升	肾病综合征
血　尿	无	镜下血尿	严重血尿	严重血尿，带血块	泌尿道梗阻
肺	无症状	症状轻微	活动后呼吸困难	休息时呼吸困难	需完全卧床
发热（药物性）	无	<38℃	38～40℃	>40℃	发热伴低血压
过　敏	无	水肿	支气管痉挛，不	支气管痉挛，需注	过敏反应

(C)1994-2022 China Academic Journal Electronic Publishing House. All rights reserved.　http://www.cnki.net

			需注射治疗	射治疗	
皮　肤	无	红斑	干性脱皮，水疱、搔痒	湿性皮炎，溃疡	剥脱性皮炎、坏死，需手术
头　发	无	轻度脱发	中度，斑状脱发	完全脱发，可再生	脱发，不能再生
感染（特殊部位）	无	轻度感染	中度感染	重度感染	重度感染伴低血压
节率	正常	窦性心动过速，休息心率>100 次/分	单灶性 PVC，房性心律失常	多灶性 PVC	室性心律不齐
心功能	正常	有异常心脏征象	短暂心功不足，不需治疗	心功不足，治疗有效	心功不足，治疗无效
心包炎	无	有心包积液，无症状	有症状，不需抽积液	心包填塞，需抽积液	心包填塞，需手术治疗
神经系统					
神志	清醒	短暂时间嗜睡	嗜睡时间不及清醒的 50%	嗜睡时间超过清醒的 50%	昏迷
周围神经	正常	感觉异常或腱反射　减退	严重感觉异常或轻度无力	不能忍受的感觉异常或显著运动障碍	瘫痪
便秘	无	轻度	中度	腹胀	腹胀，呕吐
疼痛（非肿瘤引起）	无	轻度	中度	严重	难控制

※N=正常值上限。急性和亚急性毒副反应系 1970 年 WHO 公布的表现和分度标准。

(C)1994-2022 China Academic Journal Electronic Publishing House. All rights reserved.   http://www.cnki.net

附表 4　肺癌中医临床症候评分表

中医临床症候评分参照卫生部药政司颁布的《中药新药临床研究指导原则》选取咳嗽、咳血、胸闷、气短、乏力、发热为主要观察症候，设定症状分级评分及疗效评定标准。常见症状根据临床观察分为 4 级:计分方法是无症状计 0 分、轻度计 1 分、中度计 2 分、重度计 3 分。

症状	无症状	轻度	中度	重度
积分	0	1	2	3
咳嗽	无	偶有	间断咳嗽	咳嗽频作
咳血	无	痰中血丝	痰中血丝	咳血
胸痛	无	偶有	胸痛时发	经常发作
气短	无	稍有气短	动则气短	静息气短
乏力	无	能体力劳动	日常工作	不能工作
发热	无	＞37.5℃	＞38.5℃	＞39.5℃

附表 5　肺癌患者生活质量 Karnofsky 评分法 (KPS，百分法)标准

100　正常，无症状及体征，无疾病证据

90　能正常活动，但有轻微症状及体征

80　勉强可进行正常活动，有某些症状或体征

70　生活可自理，但不能维持正常生活或工作

60　有时需人扶助，但大多数时间可自理，不能从事正常工作

50　需要一定的帮助和护理，以及给予药物治疗

40　生活不能自理，需特别照顾及治疗

30　生活严重不能自理，有住院指征，尚不到病重

20　病重，完全失去自理能力，需住院给予积极支持治疗

10　病危，临近死亡

0　死亡

(C)1994-2022 China Academic Journal Electronic Publishing House. All rights reserved.　http://www.cnki.net

附表 6　　FACT-L 生活质量评分量表

最近 7 天内：	一点也不	有一点点	有几分	颇有一些	非常多
身体状况					
1.我缺乏精力	0	1	2	3	4
2.我有呕吐	0	1	2	3	4
3.我不能胜任家庭的日常生活	0	1	2	3	4
4.我有疼痛	0	1	2	3	4
5.我被因治疗引起的毒性反应所困扰	0	1	2	3	4
6.通常我很虚弱	0	1	2	3	4
7.我不得不卧床	0	1	2	3	4
社交/家庭生活					
1.我得到了朋友的亲近	0	1	2	3	4
2.我从家庭中得到了精神支持	0	1	2	3	4
3.我得到了朋友的支持	0	1	2	3	4
4.我的家庭接受我的疾病	0	1	2	3	4
5.我对和家庭间关于病情的交流感到满意	0	1	2	3	4
6.我觉得和我的伴侣很亲近（或是我认为最重要的人）	0	1	2	3	4
7.撇开您现在的性欲活动，请您回答下面的问题，如果有不愿意回答，请在下面做个标记					
□ 回答下一个环节					
我满意我的性生活	0	1	2	3	4
情绪					
1.我很悲伤	0	1	2	3	4
2.我很自豪我能面对疾病	0	1	2	3	4
3.在于疾病斗争中，我感到失望	0	1	2	3	4
4.我感到紧张	0	1	2	3	4
5.我害怕死亡	0	1	2	3	4
6.我担心我的情况会变得更坏	0	1	2	3	4

(C)1994-2022 China Academic Journal Electronic Publishing House. All rights reserved.　http://www.cnki.net

活动能力					
1.我能工作(包括在家里进行的工作)	0	1	2	3	4
2.我工作得很充实(包括在家里工作)	0	1	2	3	4
3.我此时此刻还十分享受生活	0	1	2	3	4
4.我能接受我的疾病	0	1	2	3	4
5.我睡眠好	0	1	2	3	4
6.我进行以前的休闲活动	0	1	2	3	4
7.我目前很关心我的生活质量	0	1	2	3	4
症状/副作用					
1.我感到气短.	0	1	2	3	4
2.我的体重在下降.	0	1	2	3	4
3.我思维清晰	0	1	2	3	4
4.我有咳嗽	0	1	2	3	4
5.我为脱发而烦恼..	0	1	2	3	4
6.我的食欲很好	0	1	2	3	4
7.我感到胸闷	0	1	2	3	4
8.我呼吸通畅	0	1	2	3	4
9.您曾吸烟吗?	0	1	2	3	4
不☐ 是☐ 如果是请回答：					
我对吸烟感觉后悔	0	1	2	3	4

附表 7　　RECIST 肿瘤实体疗效评价标准

疗效	RECIST (最长径总和变化)
CR	全部病灶消失维持 4 周
PR	缩小 30%维持 4 周
SD	非 PR/PD
PD	增加 20%,病灶增加前非 CR/PR/SD

(C)1994-2022 China Academic Journal Electronic Publishing House. All rights reserved.　http://www.cnki.net

裴氏扶正抑瘤汤配合化疗治疗老年晚期非小细胞肺癌临床观察

病例登记报告表

（Case Report Form，CRF）

患 者 姓 名：____________________

受试者拼音缩写：□ □ □ □

研究 开始 日期：____________________

研究 结束 日期：____________________

(C)1994-2022 China Academic Journal Electronic Publishing House. All rights reserved. http://www.cnki.net

临床研究受试者知情同意书

尊敬的　　　　　女士/先生：

我们邀请您参加一项名称为“裴氏扶正抑瘤汤配合化疗治疗老年晚期非小细胞肺癌临床观察”的临床研究。请您仔细阅读下列试验观察的内容，如果您同意参加该项临床试验，请您在知情同意书上签字。

1 主要研究者

甘肃中医药大学中西医结合临床研究生

2 研究药物

本课题的研究过程在甘肃省肿瘤医院中西医结合科进行，所使用的处方裴氏扶正抑瘤汤是裴正学教授临床应用多年的治疗肺癌效方，其药物组成为：太子参、北沙参、潞党参、人参须、生地、山萸肉、黄芪、丹参、当归、白芍、女贞子、墨旱莲、白花蛇舌草、龙葵、半枝莲，此方具有扶正固本、破积消癥、活血化瘀、清热解毒的功效。

3 研究目的

本研究以客观评价裴氏扶正抑制瘤汤对老年晚期非小细胞肺癌的临床疗效及用药安全为出发点，主要观察裴氏扶正抑制瘤汤对老年晚期非小细胞肺癌患者目标瘤体近期客观疗效及肿瘤标志物等指标以及患者生存质量和化疗毒副作用的影响，为在临床更好的应用该方及进一步的科学研究提供依据。

4 安全性

本课题研究采用的检查方法是安全的，所采取的检查措施是临床已经应用的安全的方法，若在试验过程及试验后发生的与本研究有关的不良反应可获得及时、必要的治疗。

5 收到伤损后的补偿

如果您的健康确因参加这项研究而发生与研究相关的损害，请立即通知研究医生，他们将负责对您采取适当的治疗措施。我们将承担治疗费用及按国家有关规定对您给予相应的经济补偿。对因医疗事故或因未遵循研究方案程序而导致的损伤，申办者不予补偿。

6 自愿原则

本研究采取自愿参加的原则，您有权利选择参加或不参加本试验。我们保证您在任何时间退出本试验的自由，对你的正常治疗将不会受到您所做决定之影响。

(C)1994-2022 China Academic Journal Electronic Publishing House. All rights reserved.　http://www.cnki.net

7 保密原则

您所有的记录信息我们会专门保存在医院资料室，任何有关本项研究结果的公开报告将不会披露您的个人信息。我们将在法律允许的范围内，尽一切努力保护您个人医疗资料的隐私。

在试验过程中请您严格按照医嘱进行检查和治疗，使试验结果真实有效。

患者承诺：

我已详细了解本次研究的目的、意义及具体的研究方法、可能的获益及风险，同意参加本次研究，并会按照研究要求与研究人员配合，按时复诊，及时报告不适。

研究者签名：　　　　联系电话：　　　　日期：

受试者签名：　　　　联系电话：　　　　日期：

(C)1994-2022 China Academic Journal Electronic Publishing House. All rights reserved. http://www.cnki.net

入组选择表

请回答以下问题

**纳入标准**	是	否	无关
1. 符合诊断的晚期（IIIB 期、Ⅳ期）NSCLC 患者	☐	☐	☐
2. 符合气血亏虚，邪毒留滞症候诊断标准	☐	☐	☐
3. 符合化疗适应症；近 1 月未行放疗或化疗治疗	☐	☐	☐
4. 有一个可以精确测量的病灶（病灶螺旋 CT 或 MRI＞10mm）	☐	☐	☐
5. 年龄在 60 至 75 岁之间	☐	☐	☐
6. KPS 评分≥60 分者	☐	☐	☐
7. 预计生存期在 3 个月以上	☐	☐	☐
8. 自愿参加本研究，签署知情同意书者	☐	☐	☐

上述任何一项原则“否”时，不纳入试验。

**排除标准**	是	否	无关
1. 年龄＜60 岁或＞75 岁	☐	☐	☐
2. 危重患者或预期生存期＜3 个月的患者	☐	☐	☐
3. 严重的心、肝、肾、脑等相关脏器疾病及骨髓造血功能疾病者	☐	☐	☐
4. KPS 评分＜60 分者	☐	☐	☐
5. 近 1 周内服用过类似药物而影响疗效者	☐	☐	☐
6. 对多种药物过敏或者过敏体质者	☐	☐	☐

上述任何一项原则“是”时，该患者不被纳入试验

(C)1994-2022 China Academic Journal Electronic Publishing House. All rights reserved. http://www.cnki.net

受试者基本情况表

日 期：201 年 月 日

性别	男口 女口		体重：____kg； 身高：____cm	
年龄	________岁	住院情况	住院号:________________	
组别	对照组 □		治疗组 □	
生命体征	血压：____/___mmHg，心 率 ：____次/分，呼吸：____次/分，体温：____℃			
诊断及分期				
既往史				
过敏史				
症候积分				
卡氏评分				
生存质量				
目标瘤体				
血常规	项目		单位	临床意义判定
	WBC		$\times10^9$/L	1*口 2*口 3*口 4*口
	HGB		g/L	1*口 2*口 3*口 4*口
	PLT		$\times10^9$/L	1*口 2*口 3*口 4*口
肿瘤标志物	CEA		μg/L	
	CY21-1		μg/L	1*口 2*口 3*口 4*口
肝功	ALT		U/L	
	AST		U/L	
肾功	BUN		mmol/L	1*口 2*口 3*口 4*口
	Cr		μmol//L	1*口 2*口 3*口 4*口
粪常规	如异常，请描述：			1*口 2*口 3*口 4*口
尿常规	如异常，请描述:			1*口 2*口 3*口 4*口

注：临床意义判定：1*正常，2* 异常但无临床意义，3*异常且有临床意义，4*未查

(C)1994-2022 China Academic Journal Electronic Publishing House. All rights reserved. http://www.cnki.net

中医症状记录评分表【中药新药临床研究指导原则】

项目	正常（0 分）	轻度（1 分）	中度（2 分）	重度（3 分）	治疗前	治疗后
咳嗽	无	偶有	间断咳嗽	咳嗽频作		
咳血	无	痰中血丝	痰中血丝	咳血		
胸痛	无	偶有	胸痛时发	经常发作		
气短	无	稍有气短	动则气短	静息气短		
乏力	无	体力劳动	日常工作	不能工作		
发热	无	＞37.5℃	＞38.5℃	＞39.5℃		
纳呆	无	饮食无味	食欲差	无食欲		
嗳气	无	偶有，声轻	较频，声较响	频作，声响		
恶心呕吐	无	偶有恶心，欲吐	常有恶心，呕吐每日 2~4 次	恶心不息，呕吐频作，每日 4 次以上		
大便溏泄	无	大便稀软不成形，日行 2~3 次	烂便、溏便，日行 4~5 次或稀便日行 1~2 次	稀水样便，日行 3 次以上		
心烦	无	偶有心烦	时有心中懊侬	常常心中烦如焚		
情绪抑郁	无	情绪低落，言语减少	忧郁寡言，表情淡漠	悲观失望，沉默不语		
面色晦暗	无	萎暗不泽	黯黑无光	黧黑干枯		
		便干，1	便干，便	便干，便		

(C)1994-2022 China Academic Journal Electronic Publishing House. All rights reserved. http://www.cnki.net

大便干结	无	次/日	难，1 次/2 日	难，1 次/3 日以上		
溲赤	无	发黄	黄而少	黄赤不利		
失眠	无	睡而不稳，晨醒过早	日睡眠不足 4 小时	彻夜难眠		
盗汗	无	寐则汗微出	寐则汗出，但不湿衣	寐则汗如水，湿衣		

(C)1994-2022 China Academic Journal Electronic Publishing House. All rights reserved. http://www.cnki.net

随访记录 1：

日期：201 年 月 日

性别	男口 女口		体重：____kg； 身高：____cm	
年龄	________岁	住院情况	住院号:________________	
组别	对照组 □		治疗组 □	
生命体征	血压：____/___mmHg，心 率 ：____次/分，呼吸：____次/分，体温：____℃			
诊断及分期				
既往史				
过敏史				
症候积分				
卡氏评分				
生存质量				
目标瘤体				
血常规	项目		单位	临床意义判定
	WBC		$\times 10^9/L$	1*口 2*口 3*口 4*口
	HGB		g/L	1*口 2*口 3*口 4*口
	PLT		$\times 10^9/L$	1*口 2*口 3*口 4*口
肿瘤标志物	CEA		μg/L	
	CY21-1		μg/L	1*口 2*口 3*口 4*口
肝功	ALT		U/L	
	AST		U/L	
肾功	BUN		mmol/L	1*口 2*口 3*口 4*口
	Cr		μmol//L	1*口 2*口 3*口 4*口
粪常规	如异常,请描述：			1*口 2*口 3*口 4*口
尿常规	如异常，请描述:			1*口 2*口 3*口 4*口

注：临床意义判定：1*正常，2*异常但无临床意义，3*异常且有临床意义，4*未查

(C)1994-2022 China Academic Journal Electronic Publishing House. All rights reserved. http://www.cnki.net

随访记录 2：

日期：201　年　月　日

性别	男口　　女口		体重：____kg；　身高：____cm	
年龄	________岁	住院情况	住院号:________________	
组别	对照组　□		治疗组　□	
生命体征	血压：____/___mmHg，心率：____次/分，呼吸：____次/分，体温：____℃			
诊断及分期				
既往史				
过敏史				
症候积分				
卡氏评分				
生存质量				
目标瘤体				
血常规	项目		单位	临床意义判定
	WBC		×10^9/L	1*口 2*口 3*口 4*口
	HGB		g/L	1*口 2*口 3*口 4*口
	PLT		×10^9/L	1*口 2*口 3*口 4*口
肿瘤标志物	CEA		μg/L	
	CY21-1		μg/L	1*口 2*口 3*口 4*口
肝功	ALT		U/L	
	AST		U/L	
肾功	BUN		mmol/L	1*口 2*口 3*口 4*口
	Cr		μmol//L	1*口 2*口 3*口 4*口
粪常规	如异常,请描述：			1*口 2*口 3*口 4*口
尿常规	如异常，请描述:			1*口 2*口 3*口 4*口

注：临床意义判定：1*正常，2* 异常但无临床意义，3*异常且有临床意义，4*未查

(C)1994-2022 China Academic Journal Electronic Publishing House. All rights reserved.　http://www.cnki.net

随访记录 3：

日期：201　年　月　日

性别	男口　女口		体重：____kg；身高：____cm	
年龄	________岁	住院情况	病历号（住院口/门诊口）:____________	
组别	对照组　□		治疗组　□	
生命体征	血压：____/___mmHg，心率：____次/分，呼吸：____次/分，体温：____℃			
诊断及分期				
既往史				
过敏史				
症候积分				
卡氏评分				
生存质量				
目标瘤体				
血常规	项目		单位	临床意义判定
	WBC		×10^9/L	1*口 2*口 3*口 4*口
	HGB		g/L	1*口 2*口 3*口 4*口
	PLT		×10^9/L	1*口 2*口 3*口 4*口
肿瘤标志物	CEA		μg/L	
	CY21-1		μg/L	1*口 2*口 3*口 4*口
肝功	ALT		U/L	
	AST		U/L	
肾功	BUN		mmol/L	1*口 2*口 3*口 4*口
	Cr		μmol//L	1*口 2*口 3*口 4*口
粪常规	如异常,请描述：			1*口 2*口 3*口 4*口
尿常规	如异常，请描述:			1*口 2*口 3*口 4*口

注：临床意义判定：1*正常，2*异常但无临床意义，3*异常且有临床意义，4*未查

(C)1994-2022 China Academic Journal Electronic Publishing House. All rights reserved.　http://www.cnki.net

依从性情况

依从性	☐ 良好　　　☐ 不好（用药后第____日起出现依从性差）
原　因	☐ 不按时用药　☐自用其他药物　　☐拒绝检验<br>☐ 其他______________________

试验完成情况总结

患者末次用药日期：　　　☐☐☐☐ 年☐☐月☐☐日<br>该患者试验期间是否有不良事件发生：☐是　☐否<br>如果有不良事件，是否均已解决：　☐是　☐否<br>如否，应监测不良事件直到稳定或解决<br>患者的随机号是否被揭盲：　☐是　☐否<br>患者是否按方案完成了临床试验　☐是　☐否<br>如否，请填写以下项目：
患者中止试验日期为：☐☐☐☐ 年☐☐月☐☐日<br>最后使用药物时间为：☐☐☐☐ 年☐☐月☐☐日<br>最后复诊时间为：☐☐☐☐ 年☐☐月☐☐日<br>首先提出中止试验的是：（选择一个）☐患者<br>☐试验研究者<br>☐其它<br>若为其它，请指明________________________<br>中止试验的主要原因：（选择一个）<br>☐不良事件（已填写不良事件表）；<br>☐耐药者；<br>☐疗效不佳；<br>☐违背试验方案；<br>☐失访（包括受试者自行退出）；<br>☐患者撤回知情同意书；

(C)1994-2022 China Academic Journal Electronic Publishing House. All rights reserved.　http://www.cnki.net

□其他；

合并用药

该受试者治疗期间是否合并用药：□ 否 □ 是（若“是”，请填写下表）			
药 物 名 称	剂量	开始日期	结束日期/末次就诊时仍用
		____年__月__日	____年__月__日
		____年__月__日	____年__月__日
		____年__月__日	____年__月__日
		____年__月__日	____年__月__日

研究者签名________________ 日期： ___________年_____月____日

(C)1994-2022 China Academic Journal Electronic Publishing House. All rights reserved. http://www.cnki.net

不良事件记录

试验期间是否发生不良事件：□否　　□是；如是，请填下表：

症状	轻(1)	中(2)	重(3)	发生日期	结束日期	转归			与研究药物关系				
						消失	缓解	持续	有关	可能有关	可能无关	无关	无法评定
	□	□	□	□□年<br>□□月<br>□□日	□□年<br>□□月<br>□□日	□	□	□	□	□	□	□	□
	□	□	□	□□年<br>□□月<br>□□日	□□年<br>□□月<br>□□日	□	□	□	□	□	□	□	□
	□	□	□	□□年<br>□□月<br>□□日	□□年<br>□□月<br>□□日	□	□	□	□	□	□	□	□
	□	□	□	□□年<br>□□月<br>□□日	□□年<br>□□月<br>□□日	□	□	□	□	□	□	□	□
	□	□	□	□□年<br>□□月<br>□□日	□□年<br>□□月<br>□□日	□	□	□	□	□	□	□	□
	□	□	□	□□年<br>□□月<br>□□日	□□年<br>□□月<br>□□日	□	□	□	□	□	□	□	□
	□	□	□	□□年<br>□□月<br>□□日	□□年<br>□□月<br>□□日	□	□	□	□	□	□	□	□

(C)1994-2022 China Academic Journal Electronic Publishing House. All rights reserved. http://www.cnki.net

	□	□	□	□□年<br>□□月<br>□□日	□□年<br>□□月<br>□□日	□	□	□	□	□	□	□	□

注：

是否为严重不良事件：　□是　□否　如“是”，请填写 SAE 表

是否由此终止治疗：　□是　□否

对不良事件的处理：　□否　□对症处理　□需住院治疗　□其他

对受试药品采取的措施：□无　□减少用药次数或短暂停用　□永久停用

研究者签名_______________　日期□□□□年□□月□□日

(C)1994-2022 China Academic Journal Electronic Publishing House. All rights reserved.　http://www.cnki.net

审 核 声 明

本人作为该试验负责人特此声明：经审核，此病例报告表中所有项目的记录都是真实、完整和准确的。

试验负责人签字：______________

□□□□年□□月

(C)1994-2022 China Academic Journal Electronic Publishing House. All rights reserved. http://www.cnki.net

文献综述

老年晚期非小细胞肺癌的中西医研究进展

原发性肺癌（后文简称肺癌）是人民生命及健康的第一大肿瘤[1]。近年来，随着我国人口老龄化问题的逐渐加重，老年肺癌的发病率上升趋势更加明显[2]。肺癌起病过程隐匿，无特征性症状，且老年人对于疾病症状敏感性大大下降，大部分肺癌在发现时已经处于肺癌进展后期，失去了利用手术的方法去根治的机会，只有通过化放疗等其他治疗方法进行姑息性的治疗，且预后比较差。肺癌已经成为影响老年人健康的重要因素，关于老年肺癌的研究应该受到更多的重视，关于老年肺癌的研究也已成为一个热点 [3]

1 肺癌的流行病学

世界卫生组织癌症研究署（International Agency for Research on Cancer, IARC）2014年发布的报告显示：2012 年全球新发肺癌约 180 万，死亡约 160 万，分别占恶性肿瘤新发病例和死亡病例的 13%和 19.4%，占全部恶性肿瘤的第一位[4]。我国肿瘤登记中心于 2014 年发布了 2010 年登记的情况，显示新发肺癌患者为 60.59 万人(男性 41.63 万，女性 18.96 万)，其新发病例超过了先前多发的胃癌和肝癌，肺癌的新发病例占比达到了 19.59％(男性 23.03％，女性 14.75％)，居于首位(男性首位，女性第 2 位)，同期，死于肺癌的人数为 48.66 万(男性 33.68 万，女性 16.62 万)，在因恶性肿瘤致死的比例中达到 24.87％(男性 6.85％，女性 21.32％)。报告显示，同期我国肺癌发病率和死亡率分别是 35.23／10 万(男性 49.27／10 万，女性 21.66／10 万)和 27.93／10 万(男性 39.79／10 万，女性 16.62／10 万)。非小细胞肺癌在肺癌的病理分型中占比最高，是其总数的 80-85%，在这部分患者中，约 50%的患者年龄在 65 岁以上，30-40%的患者年龄在 70 岁以上，80 岁以上者占约 10%[5]。

2 现代医学对肺癌发病机制的认识

3.1.1 吸烟：研究[6]已经对于肺癌与吸烟的关系进行了比较精细的分析，证实肺癌与吸烟的关联性强，且对所有肺癌类型都是一致的，与计量的相关性和对协变量调整不敏感，进一步强调了这种因果关系。且肺癌的发病危险程度与吸烟的支数成正比例关系，被动吸烟也是重要的危险因素[7]。吸烟量的多少在肺癌发生后，对肺癌患者的实验指标也有很大影响[8]。

3.2.2 环境污染：国际癌症研究机构（IARC）已经将室外空气污染和颗粒物质（PM）归为致癌的空气污染，来自欧洲和北美的高质量流行病学研究数据一致显示肺癌与 PM

(C)1994-2022 China Academic Journal Electronic Publishing House. All rights reserved. http://www.cnki.net

暴露以及其他空气污染指标的正相关性，这些指标在对重要的肺癌风险因素进行调整后依然存在，我国的城市污染已经相当严重，这种相关性在我国为数不多的流行病学报告中也有显示。[9-11]由于室内的燃烧有机物、煤炭等以及室内的装修物散发的致癌物质也是一类重要的导致肺癌高发的原因[12]。

3.2.3 职业因素：缺乏有效保护的职业暴露是导致肺癌的又一重要原因。目前广泛认可的是暴露于氡、砷及石棉中，其中氡的暴露在肺癌发生中是仅次于吸烟的危险因素[13-15]。同时，如火电工人所接触的煤焦油、暴露于石棉中的矿工、石油开采工人、铺路工人接触的沥青、砷、致癌性碳氢化合物等也是肺癌高发的诱发因素，其中未分化小细胞癌和鳞癌的发病率较高。[16-17]

3.2.4 肺部慢性疾患：肺部慢性疾患的存在在肺癌的发生中扮演了非常重要的角色，研究表明[18-19]慢性阻塞性肺疾病是肺癌发生的一把推手，其可能的机制是由于长期的肺气肿，炎症所致的氧化应激及抑制 DNA 损伤的修复增加了基因突变的几率。

3.2.5 个体因素：个体的遗传特性[20]、饮食、免疫力、用药等因素也是肺癌发生的重要原因。

3 对老年人的认识

在非小细胞肺癌中的发病人群中，老年人占比超过 50%，加之老年人有其特殊的生理病理特点，在诊治过程中必须更加个体化的考虑临床所采取治疗措施，这就需要对老年人有一个比较广泛接受的认定。中医很早就关注了老年人的病理生理特点，《黄帝内经》就已经提出了关于“老”的年龄界限，如《灵枢·卫气失常》曰“人年五十已上为老”。目前国际上对老年人群的划分尚无统一的标准，发达国家和发展中国家对老年人的定义有所不同。WHO1982 年“老龄问题世界大会”关于老年人的界定，考虑到发展中国家的实际情况，以 60 岁为老年认的起点，规定：60-74 岁为年轻老人；75-89 岁为老年人：90 岁以上为非常老的老年人或长寿老年人。

4 与肺癌相关之老年患者特点

总结各报道文献[21-27]，老年肺癌患者具有以下基本特点：

2.1 老年人更多的危险因素的暴露，更高的吸烟率；

2.2 症状较为隐匿，发现时多已界中晚期；

2.3 病理类型以鳞癌较多，腺癌次之，病情发展较为缓慢；

2.4 老年患者普遍脏器功能较为低下，多合并多系统慢性疾病，对于手术及放化疗耐受性较为低下；

(C)1994-2022 China Academic Journal Electronic Publishing House. All rights reserved. http://www.cnki.net

2.5 老年人的营养及睡眠是影响生存质量的重要因素，需要关注；

2.6 心理较为脆弱，需要更多的照顾；

2.7 受教育程度偏低，需要更耐心的解释。

5 祖国医学对肺癌的认识

传统中医并无“肺癌”的诊断名词，然而历代中医名家对于肺癌的临床表现已有诸多认识。如：《素问·奇病论》中关于“息积”的论述，谓“…，气逆，二、三岁不已，…”其次《难经》关于息贲的描述，如：“在右胁下”、“ 令人洒淅寒热”、“ 喘咳，发肺壅”是适合肺癌临床表现的。后世医书如《济生方》、《圣惠方》等书亦描述了与肺癌相似的症状。综上，可将肺癌归属于中医肺积、息贲、咯血、虚劳等范畴。

5.1 肺癌的中医病因病机

祖国医学认为肺癌的发生是体内外多因素共同作用的结果。张星星等[28]在总结建国以来发表在中国知网上的文献，以“肺癌”、“ 中医”为检索词，综合文献提出中医关于肺癌病因病机的研究主要从正气虚损论、邪毒侵肺论、痰瘀内聚论、其它学说 4 个方面开展。张怡等[29]基于因子和聚类分析认为肺癌为本虚标实，虚实夹杂之病，脏腑涉及肺、脾、肾三脏，正气虚弱，痰湿、气滞、血淤久聚是发病的主要原因。

5.2 肺癌的中医辨证分型

辨证论治是中医学在整体观外又一重要思想，辨证论治使得中医学可以及时的了解疾病发展在特定时间里比较全面的情况。孙青等[30]认为在肺癌中单证气虚、阴虚、血淤、痰证出现的几率较大，而单证的出现在肺癌的病理分型及分期的比较中无统计学意义，复合证型的出现比较复杂，建议以单证的辨识与组合作为肺癌辨证分型的有效方法。

5.3 肺癌中医辨证与治疗相关

张越等[31]总结 31 例肺癌患者，发现证型在化疗前后会发生由实证向虚证的转化，指出化疗前多以实证为主，化疗后以虚证为主，其中以气虚和气阴两虚最为常见。屠洪斌等[32]认为，靶向药物的治疗可使肺癌的中医辨证分型发生变化，靶向药物治疗后可以使肺阴虚证的患者增加。

5.4 肺癌的中医辨证分型与体质相关

肺癌发生发展、辨证分型及预后亦与中医体质与存在相关性。孟博等[33]认为体质是影响肺癌辨证分型和预后的重要因素，提出肺癌的体质以气虚质和阳虚质为多见，特禀质和血瘀证少见，体质的偏性以虚证为主，与中医正虚发病的发病说一致，在具体的体质与证型分布上因地域的不同会有所差异。

(C)1994-2022 China Academic Journal Electronic Publishing House. All rights reserved. http://www.cnki.net

6 肺癌的中医治疗

6.1 对老年晚期肺癌治疗前的中西医结合评估

老年人在机体功能、营养、合并症、心理及社会关系、对药物的耐受性等方面有其特殊的病理生理特点，加之肺癌已进展至晚期，因此应该在治疗前对病人各个方面的状况进行中西医结合的评估，以便后期的治疗能够尽可能的照顾到各个方面的差异，围绕病人的生存质量，采取更为个体化的治疗措施。刘杰等[34]认识到不能对老年和青年人仅仅以年龄数字上的变化加以简单的区分，而是一个多维、综合性的评价以更合理的定出老年人的“生物学年龄”，应当通过实验室检查、影像学、KPS 评分、运动情况、合并症、营养、抑郁情况等方面进行综合评估。中医通过传统的望闻问切等诊断手段，从脏腑、气血、阴阳等角度进行评估。

6.2 肺癌的中西医结合治疗

当前肺癌经典的治疗普遍存在着缺陷和不足，治疗所产生的毒副作用，较为严重的后遗症在老年晚期肺癌患者尤其明显，靶向治疗和生物治疗在存在前述问题的同时，其治疗所需的高昂费用也令老年患者望而却步，这使得上述治疗在老年晚期肺癌患者的应用范围减小。中医药在肺癌的治疗中获得一定效果。加之中医药现代化的快速发展，中医药的剂型改进，使得中医药的接受度更广，老年晚期肺癌以中医药为主要治疗手段的治疗思路逐渐形成。中医药相对于西医，能够更好的调剂患者机体的反应性。肺癌是以局部癌肿为中心的全身病变，因此，中西医结合治疗发挥各自的优势，在临床上已经成为治疗肺癌的共识。

6.2.1 单纯中医药治疗　当肿瘤进展至终末期阶段，任何严重损伤机体的治疗都已不再适合。而此时，中医药依然能够发挥一定的作用，可以在患者生命的最后缓解部分不适。中医药以专方专药和辨证论治的治疗在老年晚期肺癌的治疗上能够取得比较满意的效果。 兰万成等[35]总结著名中医专家程剑华教授治疗老年晚期肺癌的经验，指出在肺癌的治疗中中医药要全程参与，要辨证与辨病相结合。同时，在具体的治疗上提出了“要本乎肺，又要远乎肺的观点”，强调要从观念上改变过来，要把肺癌作为一种慢性病来治疗，在人瘤共存中努力提高患者的生存质量。沈丽萍等[36]采用前瞻性研究，将 88 例非小细胞肺癌患者分为两组，治疗组用以扶正固本为主的辨证论治维持治疗，对照组用单药化疗，治疗两周后治疗组相对于对照组在社会功能、认知功能、总健康状况得分及症状均有统计学差异。治疗组疾病控制率在 60.53%，对照组在 30.77%，两者差异具有统计学意义。刘莹[37]治疗 40 例肺癌患者，治疗组用单纯中医辨证论治，对照

(C)1994-2022 China Academic Journal Electronic Publishing House. All rights reserved. http://www.cnki.net

组用 NP 方案化疗，治疗后观察两组 Karnofsky 的变化，治疗组的改善优于对照组。

6.2.2 中医药配合手术　手术治疗是目前唯一有可能根治肺癌的治疗方式。大部分情况下，对于能够完整切除且没有禁忌症者，原则上尽可能选择手术。虽然老年患者因机体功能较差、合并症较多等问题，并不适宜手术干预[38]，然而手术依然可以在减轻局部肿瘤负荷及压迫症状和延缓肿瘤进展方面发挥作用。肺癌术后配合中医药在患者的恢复方面有积极价值。李远静等[39]观察 114 例肺癌术后患者，对照组用 GP 方案化疗，观察组在对照组的基础上加用中药康艾注射液和八珍颗粒，治疗 4 个疗程后对比两组患者，观察组 1 年、2 年、3 年生存率分别是 91.67%、68.75%和 31.25%，对照组的 1 年、2 年、3 年生存率分别是 77.27%、46.97%和 27.27%，1、2 年生存率有统计学差异。另外在有效率、生活质量的改善、不良反应的发生及机体的免疫功能方面亦效果显著。郑爱红等[40]观察 60 例非小细胞肺癌术后患者，证实扶正抗癌汤合并化疗能够明显改善术后肺癌患者的生活质量，减轻化疗带来的毒副反应。

6.2.2 中医药配合放疗

放疗是目前治疗非小细胞肺癌的重要手段[41]，放疗对于不能耐受手术或拒绝手术治疗的患者是一种重要的治疗措施，对于肺癌术后的患者是一个重要的减少复发转移的辅助治疗，对于肺癌晚期的患者是非常重要的姑息治疗措施。[42]随着科学技术尤其是影像学的进步，放射治疗方式也进行着日新月异的变化，精准放疗逐渐进入临床，出现了三维适形调强、束流调强和立体定向放射等先进的治疗手段，使得放射治疗时肿瘤细胞的到最大的放射剂量，同时减少了对周围正常组织损伤，大大的增强了放射治疗的增益比。因此，放射治疗的适应症进一步扩大，放射治疗所引起的毒副反应减轻。然而，放疗的毒副作用依然不可忽视。有学者在放疗的同时，联合中药制剂，能够减轻放疗的毒副作用，取得一定进展。胡慧[43]认为联合中药制剂可以减轻放疗的不良反应、改善临床症状、提高生活质量、保护骨髓等。对比 40 例非小细胞肺癌患者，发现联合参芪扶正注射液较单纯 3DCRT 有效率增加，前者为有效率 95%，后者有效率 75%，有统计学差异。联合组在改善症状如胸痛、血痰、气短及上腔静脉综合征方面有优势，其改善分别为 80.0%、70.0%、75.5%、85%，对照组为 30.0 %、25.0%、20.0%、40.0%，差异有统计学意义。其急性放射性肺炎、骨髓抑制发生率是 5.0%、10.0%对比 20.0%、30.0%，差异有统计学意义。赵亚娟等[44]联合艾迪注射液和三维适行放疗（3DCRT）治疗 40 例非小细胞肺癌患者，比较治疗前后血清中白介素-6（IL-6）、肿瘤坏死因子-α（TNF-α）和转化生长因子—β（TGF-β）水平变化及放射性肺炎（RF）发生率的差异具有统计学意

(C)1994-2022 China Academic Journal Electronic Publishing House. All rights reserved.　http://www.cnki.net

义。因此，艾迪注射液可以有效的缓解放射性肺炎的发生。

6.2.3 中医配合化疗 虽然晚期肿瘤是不可治愈的，但是化疗提供了一种有效的缓解办法。新辅助化疗可以使得癌瘤局限化，从而使肿瘤的手术后复发和转移的机会下降，也可以使一部分不能手术的患者增加手术切除的可能性。辅助治疗是肺癌手术后患者的常规治疗，其在延缓肺癌的复发转移方面效果卓著。然而化疗药物严重的毒副反应导致试图运用化疗取得更一步的疗效变得困难，在老年患者尤其如此，这使得一部分患者不能选择或者中途放弃化疗。中医药在改善患者身体状况，减缓化疗毒副反应等方面效果明显，近年来的研究在此方面取得一定进展。陆烨等[45]对比生脉散加味联合吉西他滨+顺铂化疗和单纯化疗，结果实验组治疗后患者 T 细胞亚群及细胞因子水平（$CD3^+$、$CD3^+CD8^+$、NK、NKT 和 Treg）显著升高，$CD3^+CD4^+$水平显著降低，对照组治疗后 $CD3^+$水平升高，$CD3^+$ $CD4^+$、$CD3^+$ $CD8^+$、NK、NKT 和 Treg 水平均显著降低，两组差异有统计学意义。试验组患者治疗后 IFN-γ、TNF-α、IL-2 水平升高，对照组治疗后 IFN-γ、TNF-α、IL-2 水平均显著降低，两组比较有统计学差异。王娜等[46]治疗 127 例晚期非小细胞肺癌患者，将其分为中西医结合治疗组和化疗组，前者按中医辨证分型分为五个证型，分别给予中医处方，再次基础上联合化疗药物奈达铂联合多西他赛，后者仅给予化疗治疗，结果中西医结合组患者完全缓解(CR) 3 例，部分缓解(PR) 31 例，疾病稳定(SD)20 例，客观有效率为占 52.3%，明显优于化疗组的 33.9% ($\chi^2 = 4.393$，$P = 0.027$) 。中西医结合组患者的疾病控制率为 83.1%，明显优于化疗组的 58.1%($\chi^2 = 9.616$，$P = 0.002$) 。中西医结合组对于 Karnofsky 评分和血细胞的改善及消化道的改善均优于对照组。（$P<0.05$）

6.2.4 中医药配合靶向治疗 虽然手术是目前能够根治肺癌的唯一有效手段，然而肺癌发现时 80%已处于中晚期，而放化疗的疗效差强人意，同时其严重的毒副反应也使患者望而却步，因此不得不求助于内科治疗。一项总结了 2014 年以前的肺癌靶向药物的研究显示，在 NSCLC 的治疗中，靶向药物已经取得了非常明显的进步，人们逐渐通过新一代测序比较全面的了解肺癌中基因组的改变，以及肺癌对靶向药物耐药的机制，同时，免疫检测点抑制剂的早期结果也取得了令人鼓舞的进步[47]。近年来发展出的靶向治疗以其靶向性、安全性等优势逐渐为临床认识，各类靶向治疗方式层出不穷，例如以表皮生长因子受体（epidermal growth factor receptor，EGFR）为靶点的药物、以 EML4-ALK 融合基因为靶点的药物、以 ROS1 融合基因为靶点的药物、以 RET 融合基因为靶点的药物、以 MET 基因扩增为靶点的药物、以抑制肿瘤血管新生为靶点的药物等[48]。同时，

(C)1994-2022 China Academic Journal Electronic Publishing House. All rights reserved. http://www.cnki.net

靶向药物导致的皮疹、腹泻、手足皮肤反应、高血压、间质性肺炎等也不容忽视。另外，获得性耐药已经成为限制靶向药物取得更好疗效的瓶颈，李凤珍等[49]、张小瑞等[50]、刘浩[51]证实中医药能够改善靶向药物毒副反应及获得性耐药，使靶向药物的临床疗效更进一步。

6.2.5 肺癌的生物治疗　肿瘤的生物治疗经过多年的发展已经有了自己较为成熟的理论与技术系统。众多新型生物标志物的发现及其机制的进一步明确，使得 LC 的诊断更加准确[52]。研究发现[53]细胞外基质蛋白（ECM）和中间丝蛋白（IMF）两类蛋白的失调和破坏可能是恶性变化的一部分，其可能在肺癌的诊断中具有巨大潜力。与胃肠癌、肺癌、前列腺癌等的比较中，发现肺癌中 ECM 和 IMF 的阳性预测值更高。肿瘤生物治疗可以更为主动的调动机体的反应性。从而在肿瘤的治疗中充分发挥机体自身的抗病能力。抗癌免疫应答和肿瘤免疫抑制机制的进展使预苗和靶向药物进入肺癌的治疗，过去对于肺癌对免疫相对耐受的观点受到质疑[54]。肺癌的生物治疗开辟了肺癌全新治疗模式的同时也丰富了肿瘤生物治疗范围[55]。肿瘤疫苗治疗在肺癌主动免疫治疗中扮演着重要的角色。肺癌治疗的疫苗主要包括树突状细胞(dendritic cell，DC) 疫苗，失活的自体或同种异体肿瘤细胞疫苗，相关肿瘤抗原疫苗(mRNA/DNA、蛋白、肽)和病毒疫苗[56]。主要通过回输具有特异性或非特异性抗瘤活性的生物活性物质被称为被动免疫治疗即过继性免疫治疗，这种回输的生物活性物质在患者体内直接发挥抗肿瘤效应。越来越多的证据表明，过继性免疫治疗可减少恶性肿瘤的复发率和转移率。

7 老年非小细胞肺癌治疗展望

非小细胞肺癌的高发率和致死率，以及其多发于老年人，临床早期不易被发现等特点，使目前常用的治疗方式，包括手术、放疗、化疗、介入、靶向、生物治疗等，在临床应用时都显得捉襟见肘。精准医学的提出使得肺癌的治疗走向更加个体化的治疗方向，这就需要从肺癌的诊断做起，做好肺癌高危人群的筛查工作，通过检测肺癌相关的肿瘤驱动基因，及早的发现肺癌，研究显示已知 60%～70% 的肺癌患者能找到驱动基因，针对这些驱动基因可进行靶向治疗。甚至更多分子表型的检测使得腺癌和大细胞癌以及组织分型不明的非小细胞肺癌的诊断更加确切，虽然鳞癌并无特殊的分子表型，然而通过检测知道明确的组织分型，更加优化化疗方案选择使鳞癌的生存期取得一定的延长也是事实，这都为肺癌的精准治疗或个体化治疗提供了很大的帮助。其次如何根据临床情况选择合理化综合治疗方案，在临床决策时根据患者自身的情况及病情的发展，兼顾病人的经济及家庭情况等方面，在提高治疗疗效的同时准确地评估预后。因此，在肺

(C)1994-2022 China Academic Journal Electronic Publishing House. All rights reserved.　http://www.cnki.net

癌的诊断和治疗时，要求临床医生尽可能的运用当前的最新的发现及循证医学的结果，极为耐心的关注病人的各个方面的状况来审慎的制定临床决策是今后发展的方向。

(C)1994-2022 China Academic Journal Electronic Publishing House. All rights reserved. http://www.cnki.net

参考文献

[1]Ferlay J, Soerjomataram I, Dikshit R, et al. Cancer incidence and mortality worldwide: sources, methods and major patterns in GLOBOCAN 2012.[J]. International Journal of Cancer, 2015, 136(5):E359.

[2]Smith B D, Smith G L, Hurria A, et al. Future of cancer incidence in the United States:burdens upon an aging, changing nation.[J]. Journal of Clinical Oncology Official Journal of the American Society of Clinical Oncology, 2009, 27(17):2758.

[3]Heist R S, Minokenudson M, Sequist L V, et al. FGFR1 amplification in squamous cell carcinoma of the lung[J]. Journal of Thoracic Oncology, 2012, 7(12):1775-1780.

[4]Ferlay J, Soerjomataram I, Dikshit R, et al. Cancer incidence and mortality worldwide: sources, methods and major patterns in GLOBOCAN 2012.[J]. International Journal of Cancer, 2015, 136(5):E359.

[5]支修益,石远凯,于金明.中国原发性肺癌诊疗规范(2015年版)[J].中华肿瘤杂志, 2015,37(1):67-78.

[6]Lee P N,Forey B A,Coombs K J.Systematic review with meta-analysis of the epidemiol- ogical evidence in the 1900s relating smoking to lung cancer[J]. BMC Cancer, 2012,12 (1):385.

[7]Proctor R N. The history of the discovery of the cigarette–lung cancer link: evidentiary traditions, corporate denial, global toll[J]. Tobacco Control, 2012, 21(2):87-91.

[8]周岩.吸烟指数与肺癌患者凝血指标的相关性[J].中国肿瘤临床与康复,2016 (10):1160-1162.

[9]Loomis D,Huang W,Chen G.The International Agency for Research on Cancer (IARC) e- valuation of the carcinogenicity of outdoor air pollution:focus on China.[J].Chinese Jour- nal of Cancer,2014, 33(4):189-196.

[10]Liu S, Zhou M, Wang L, et al. [Burden of disease attributable to ambient particulate matter pollution in 1990 and 2010 in China].[J]. Zhonghua yu fang yi xue za zhi [Chinese journal of preventive medicine], 2015, 49(4):357-363.

[11]涂庆,王宇,黄莉.PM2.5与肺癌相关关系及防治策略研究[J].重庆学,2016,45(8):1118-1121.

[12]3Rd H H, Wei H, Sapkota A, et al. Household coal use and lung cancer: systematic review and meta-analysis of case-control studies, with an emphasis on geographic variation.[J]. International Journal of Epidemiology, 2011, 40(3):719.

(C)1994-2022 China Academic Journal Electronic Publishing House. All rights reserved. http://www.cnki.net

[13]Lantz P M, Mendez D, Philbert M A. Radon, smoking, and lung cancer: the need to refocus radon control policy.[J]. American Journal of Public Health, 2013, 103(3):443.

[14]Lantz P M,Mendez D,Philbert M A.Radon, smoking,and lung cancer:the need to refocus radon control policy.[J]. American Journal of Public Health,2013,103(3):443.

[15]Nielsen L S,Bælum J,Rasmussen J,et al.Occupational asbestos exposure and lung cancer-a systematic review of the literature.[J]. Archives of Environmental & Occupational Health, 2014, 69(4):191-206.

[16]王思华. 焦炉作业工人职业暴露估测及其端粒损伤生物标探索[D]. 郑州大学, 2016.

[17]李媛秋, 么鸿雁. 肺癌主要危险因素的研究进展[J]. 中国肿瘤, 2016, 25(10):782-786.

[18]Brenner D R,Boffetta P, Duell E J,et al.Abstract 4826: International Lung Cancer Consortium: Pooled analysis of previous lung diseases and lung cancer risk[J].Cancer Research,2011,70(8 Supplement): 4826-4826.

[19]Durham A L, Adcock I M. The relationship between COPD and lung cancer.[J]. Lung Cancer, 2015, 90(2):121-127.

[20]Lan Q, Chao A H, Matsuo K,et al.Genome-wide association analysis identifies new lung cancer susceptibility loci in never-smoking women in Asia[J]. Nature Genetics,2012,44(12):1330-5.

[21]王勇, 姚艺玮, 钱小军等. 青年与老年肺癌患者临床特征回顾性分析[J]. 临床肺科杂志, 2016, 21(12):2150-2153.

[22]吴晓旭. 肺癌患者心理状况及相关因素分析[D]. 中国医科大学, 2013.

[23]吴慧娟, 吴红波, 邹宏志等. 老年晚期非小细胞肺癌的临床特点及对化疗的耐受性和疗效[J]. 中国老年学, 2016, 36(3):632-634.

[24]聂晓红, 张剑, 罗立. 慢性阻塞性肺疾病合并肺癌临床特点及肺功能分析[J]. 临床肺科杂志, 2016, 21(8):1478-1481.

[25]刘亚红, 熊添, 郭丽娟, 等. 老年肺癌晚期癌性疼痛处理[J]. 中国疼痛医学杂志, 2016, 22(1):77-79.

[26]杨红美, 沈玉. 老年肺癌患者围化疗期营养评估及护理干预[J]. 齐鲁护理杂志, 2016, 22(16):40-41.

[27]龚晓桃. 老年肺癌病人放弃化疗状况及相关因素分析[J]. 全科护理, 2016, 14(7):70 6-708.

[28]张星星, 李泽庚. 肺癌中医病因病机探讨[J]. 中华中医药杂志, 2015(10):3447-3449.

[29]张怡, 于明薇, 王笑民等. 基于因子和聚类分析的肺癌方药规律研究[J]. 中华中医药杂志, 2013(12):3750-3752.

(C)1994-2022 China Academic Journal Electronic Publishing House. All rights reserved. http://www.cnki.net

[30]孙青,夏莹,王景等.中晚期肺癌中医辨证分型的初步探讨[J].中华中医药杂志,2010(10):1702-1704.

[27]张越,杨焱,马伊锐.化疗前后肿瘤患者的证候规律[J].中国老年学,2015(10):2724 -2726.

[32]屠洪斌,董志毅,王少墨等.原发性肺癌中医证型规律与化疗及靶向治疗的相关性研究[J].世界科学技术-中医药现代化,2014(9):1997-2001.

[33]孟博,李杰.基于中医体质学说对肺癌的认识及预后分析[J].辽宁中医杂志,2015(6):1370-1373.

[34]刘杰,林洪生.中医治疗老年肺癌的思路与方法[J].中医杂志,2011,52(2):104-107.

[35]兰万成,朱德才,吴晓新.程剑华治疗肺癌的中医学术思想[J].辽宁中医杂志,2011(3):418-419.

[36]沈丽萍,刘苓霜,姜怡等.中医综合方案维持治疗晚期非小细胞肺癌患者改善生活质量的临床疗效研究[J].中华中医药学刊,2014(7):1677-1681.

[37]刘莹.中医治疗肺癌 40 例临床观察[J].实用中医内科杂志,2012(9S):21-22.

[38]于正伦,张曙光,张其刚等.影响肺癌手术患者生存因素研究[J].中国卫生统计,2016,33(3):466-467.

[39]远静,骆学新,孟小鹏等.非小细胞肺癌患者术后化疗结合中医辅助治疗的中期疗效观察[J].中华中医药学刊,2013(11):2564-2566.

[40]郑爱红,吴国清, 薛骞等.扶正抗癌汤对肺癌术后的临床疗效观察[J].中华中医药学刊,2013(3):698-700.

[41]Jr W J C,Paulus R,Langer C J, et al. Sequential vs Concurrent Chemoradiation for Stage III Non–Small Cell Lung Cancer:Randomized Phase III Trial RTOG 9410[J]. Journal of the National Cancer Institute, 2011, 103(19):1452.

[42]Ishikura S.Optimal radiotherapy for non-small-cell lung cancer: current progress and future challenges[J]. General Thoracic and Cardiovascular Surgery, 2012, 60(3):127-131.

[43]胡慧.参芪扶正注射液联合 3DCRT 应用于晚期非小细胞肺癌患者的疗效[J].实用药物与临床,2013,16(12):1133-1135.

[44]赵亚娟,李青山,刘兰芳等.艾迪注射液对肺癌放疗患者细胞因子表达的影响[J].中国实验方剂学杂志,2013,19(8):323-325.

[45]陆烨,王晨洁,余蓉.生脉饮加味联合化疗治疗晚期非小细胞肺癌临床研究[J].中医学报,2014(10):1400-1402.

(C)1994-2022 China Academic Journal Electronic Publishing House. All rights reserved. http://www.cnki.net

[46]王娜,朱晓燕,郭婷等.中医辨证施治配合奈达铂联合多西他赛方案治疗晚期非小细胞肺癌的疗效[J].中国肿瘤临床与康复,2015(2):171-174.

[47]Morgensztern D, Campo M J, Dahlberg S E, et al. Molecularly Targeted Therapies in Non–Small-Cell Lung Cancer Annual Update 2014[J]. Journal of Thoracic Oncology Official Publication of the International Association for the Study of Lung Cancer, 2015, 10(1):1-63.

[48]Blanco R, Iwakawa R, Tang M, et al. A gene-alteration profile of human lung cancer cell lines.[J]. Human Mutation, 2009, 30(8):1199.

[49]李凤珍,崔廷宝.对中医药治疗肿瘤靶向药物不良反应的研究与分析[J].辽宁中医药大学学报,2012(6):183-184.

[50]张小瑞,赵远红.非小细胞肺癌靶向药物相关皮肤毒性反应的中医辨治[J].中医杂志,2015,56(12):1065-1066.

[51]刘浩.扶正解毒法改善肺癌分子靶向治疗获得性耐药[J].中华中医药学刊,2016(7):1563-1565.

[52]王会中,任成山,金发光.肿瘤生物标志物在肺癌患者检测中的临床意义及研究进展[J].中华肺部疾病杂志电子版,2016,9(3):329-333.

[53]Willumsen N, Bager C L, Leeming D J, et al. Serum biomarkers reflecting specific tumor tissue remodeling processes are valuable diagnostic tools for lung cancer[J]. Cancer Medicine, 2014, 3(5):1136.

[54]Iyengar P, Gerber D E. Locally Advanced Lung Cancer: An Optimal Setting for Vaccines and Other Immunotherapies[J]. Cancer Journal, 2013, 19(3):247-262.

[55]胡春燕,何凤.肺癌生物治疗的研究进展[J].医学综述,2013,19(23):4265-4268.

[56]Winter H, Engel N K V D, Rusan M, et al. Active-specific immunotherapy for non-small cell lung cancer[J]. Journal of Thoracic Disease, 2011, 3(2):105

(C)1994-2022 China Academic Journal Electronic Publishing House. All rights reserved. http://www.cnki.net

致谢

时光荏苒，转眼三年的研究生学习已经结束，期间老师的关心，家人的支持，同学的帮助使我的学习生活充实快乐，在此表示感谢！

首先，非常感谢蒲朝晖主任，三年的学习中给了我很多指导和教育，从论文的选题、数据调查到论文撰写、修改和完成，均渗透着导师的引导。导师的渊博、严谨是我学习的榜样。

其次，特别感谢著名中西医结合专家裴正学先生！裴老先生是我的授业恩师，裴老学问博大，人情练达。裴老提出的“西医诊断，中医辨证，中药为主，西药为辅”的发展中医的“十六字方针”给我指明了努力的方向。三年来目睹裴老“老牛自知夕阳短，不用扬鞭自奋蹄”的勤奋努力，“蚕僵丝尽亦堪用，入药三分称妙丹”的无私奉献，“恬淡虚无，世无不爱”的从容豁达，这些都是裴老给我的最为宝贵的精神，是我一生享用不尽的财富。三年的陪伴，从学习生活，做人作文裴老无微不至的关怀着我。学生愚钝，然“近朱得赤”，我将钻坚仰高，继续发扬裴老的学术思想，再次衷心的感谢恩师裴老！

感谢甘肃省肿瘤医院中西医结合科薛文翰主任、万强主任、张桂琼医师、冯永笑医师、鲁维德医师、张丑丑及其他医师，感谢他们在临床上给予的指导和帮助以及生活上的关心！

感谢甘肃中医药大学给我提供的学习平台，再此我度过了美好的八年时光，见证了自己的成长，也见证了学校的发展壮大，衷心感谢研究生院的领导和老师给予我的指导和关心！

非常感谢其他师兄师姐、师弟师妹们三年来给予我的帮助和支持！

此外，感谢我的父母，在我多年的求学路上，他们毫无怨言，默默地为我付出。谢谢你们！

最后，衷心感谢百忙中参加论文评审和答辩的各位专家和老师！

(C)1994-2022 China Academic Journal Electronic Publishing House. All rights reserved. http://www.cnki.net

研究成果

攻读硕士研究生期间发表的论文和参编书目：

1. 吕鹏强, 蒲朝晖, 张顺贤,等. 裴正学教授辨治鼻咽癌组方思路浅析[J]. 亚太传统医药, 2016, 12(18):99-100.
2. 参编书目：

（1）《中国著名中西医结合专家裴正学健康微博》第四辑；

（2）《裴正学医学讲座集》。

(C)1994-2022 China Academic Journal Electronic Publishing House. All rights reserved. http://www.cnki.net
