# Supplementary material for: Benefit-risk evaluation of Fuzheng Yiliu Decoction combined with chemotherapy for treating non-small cell lung cancer using multicriteria decision analysis
Source: Front Oncol. 2025 Sep 23;15:1627904. doi: 10.3389/fonc.2025.1627904 (PMC12500661; doi:10.3389/fonc.2025.1627904)
Supplement: Supplementary file 2 [file Supplementaryfile2.zip › supplementary files/Included literature.zip!/literature/纳入文献/7张薇薇.pdf]

DOI:10.16305/j.1007-1334.2007.06.009

扶正抑瘤方对晚期非小细胞肺癌化疗增效减毒作用及TSGF的影响

张微微 陈 越 张庆荃

上海中医药大学附属岳阳中西医结合医院肿瘤科 (上海 200437)

【摘 要】 目的 观察扶正抑瘤方对晚期非小细胞肺癌化疗毒副反应的减毒作用及其对肿瘤特异性生长因子(TSGF)水平的影响。方法 将42例患者随机分为对照组(20例,采用单纯化疗治疗)和综合组(22例,化疗结合扶正抑瘤方治疗),21 d为1个周期,两组均连续治疗2个周期。比较两组患者化疗通过率,毒副反应,生活质量,血清TSGF水平。结果 综合组化疗通过率略高于化疗组,生活质量明显高于化疗组,化疗毒副反应小于化疗组,TSGF下降水平大于化疗组。结论 自拟扶正抑瘤方能够改善晚期肺癌患者生活质量,能在一定程度上降低化疗的毒副反应,并协同化疗起到抑瘤的作用。

【关键词】 扶正抑瘤方;晚期非小细胞肺癌;肺瘤特异性生长因子;化疗

【中图分类号】 R256.1;R734.2 【文献标识码】 A 【文章编号】 1007-1334(2007)06-0018-02

Effects of "Fuzheng Yiliu Decoction" on Chemotherapeutic Reactions and TSGF of Advanced Non-small Cell Lung Cancer

ZHANG Wei-wei CHEN Yue ZHANG Qing-quan

Yueyang Hospital of Integrative Chinese & Western Medicine Affiliated to Shanghai University of Traditional Chinese Medicine

Abstract:

To observe the clinical effects of "Fuzheng Yiliu Decoction" on non-small cell lung cancer and TSGF. Methods Forty-two subjects were randomized into two groups: control group in which 20 cases were treated simply by chemotherapy and treatment group in which 22 cases were treated with chemotherapy plus "Fuzheng Yiliu Decoction", with two courses. The chemotherapeutic effects, adverse reactions, quality of life and TSGF were observed. Results More patients in treatment group could endure chemotherapy; the quality of life was better, adverse reactions less and TSGF lower in treatment group than in control group. Conclusion "Fuzheng Yiliu Decoction" can improve the quality of life and relive adverse reactions in patients with advanced non-small cell lung cancer.

Key Words:

"Fuzheng Yiliu Decoction"; non-small cell lung cancer; TSGF; chemotherapy

吉西他滨联合顺铂(GP方案)化疗是目前治疗中晚期非小细胞肺癌(Ⅲ~Ⅳ期)的首选方案之一。而中医药亦是肿瘤综合治疗的重要手段,对于合理的中医治疗与西医治疗配合可以明显地提高肿瘤治疗的疗效,目前已形成共识。近年来,我们根据晚期非小细胞肺癌的特点,运用自拟扶正抑瘤方配合GP方案化疗,取得一定疗效,现总结如下。

1 资料与方法

1.1 一般资料 患者来源为本院肿瘤科2003年11月至2006年3月期间收治的原发性非小细胞肺癌患者。纳入标准:经CT、MRI等影像学检查及细胞学、组织学确诊为腺癌、鳞癌或腺鳞癌;临床分期均为Ⅳ期,卡氏评分≥60分,预计生存期在3个月以上。排除标准:伴有严重心、肝、肾病变和血液系统疾病者,严重感染患者,严重胃部疾病不能口服药物者不列入本观察。病例总数为42例,年龄52~81岁;随机分为化疗组20例、综合组22例。两组性别、年龄、病理类型、分期以及治疗前化疗疗程等一般情况无显著性差异($P>0.05$),具有可比性。

1.2 治疗方法 两组患者均予以国产吉西他滨(泽菲)联合顺铂(GP方案)化疗:泽菲1.0 g/m² 静脉滴注30 min滴完,d1、d8;顺铂30 mg/m² d1-3静脉滴入加水化。21 d为1周期,3~4周期为1个疗程,化疗周期内每周复查血象、肝肾功能及心电图。如在正常范围内则按期进行化疗,否则停药加用G-CSF治疗,并延期化疗。非血液学毒副反应的症状除恶心呕吐外,未达到WHO规定的Ⅲ级以上毒副反应者不予特殊处理,恶心呕吐反应不作为化疗药物的减量或停药标准。综合组则同时予以扶正抑瘤方(太子参、黄芪、白术、白芍药、茯苓、白花蛇舌草、开金锁、石上柏、鱼腥草、石见穿、炙枇杷叶、象贝母、黄芩、灵芝等)加减治疗,每日1剂,分早晚2次口服。

1.3 观察指标 心肝肾及造血系统的损害,生活质量(卡氏评分、体重),化疗通过率,恶性肿瘤特异性生长因子(TSGF)水平(每周期化疗前均检测肿瘤标志物TSGF的表达量)。

[作者简介] 张微微(1970-),女,浙江青田人,大学本科,主治医师,主要从事中西医结合肿瘤临床。

(C)1994-2023 China Academic Journal Electronic Publishing House. All rights reserved. http://www.cnki.net

1.4 评价方法 ①血象(包括白细胞总数、中性粒细胞数、血小板、血红蛋白)按 WHO 急性、亚急性毒性标准(五级)。②TSGF 测定:应用生化比色法检测(福建新大陆生物技术有限公司专项药盒),按诊断试剂盒中所附的说明书,严格按规程操作。TSGF 含量<64×10³ U/L 为阴性,64×10³~71×10³ U/L 为可疑阳性,>71×10³ U/L 为阳性。

1.5 统计学方法 统计分析将采用 SPSS 11.5 统计分析软件进行计算。采用配对 t 检验比较组内前后差异,两组治疗前后的变化采用方差分析进行比较,两组治疗前后的变化采用 χ^2 检验或非参数检验。

2 结果

2.1 化疗通过率 两组患者第 1 周期化疗顺利通过率均为 100%,第 2 周期综合组化疗顺利通过率为 90.9%,略高于化疗组(90.0%),但无统计学意义($P>0.05$)。

2.2 生活质量 卡氏评分(体力状况计分)在第 1 周期时,综合组类似于化疗组($P>0.05$);在第 2 周期末综合组明显优于化疗组($P<0.05$)。两组在第 1、2 周期的体重基本没有变化($P>0.05$)(表 1)。

表 1 两组生活质量比较

时间	综合组		化疗组	
	卡氏评分	体重(kg)	卡氏评分	体重(kg)
1 周期初	80.14±8.90	62.82±11.30	79.24±8.60	65.56±13.72
1 周期末	77.72±9.61	62.81±12.28	75.54±8.98	64.79±12.06
2 周期初	78.63±8.50	62.83±12.30	76.02±8.78	65.07±14.64
2 周期末	77.17±8.75*	62.92±11.22	71.05±9.84	65.01±13.50

注:与化疗组同期比较,*$P<0.05$。下同。

2.3 化疗毒性反应 对血象影响:综合组与化疗组比较,血红蛋白第 1 周期与第 2 周期均有显著差异($P<0.05$),即综合组优于化疗组;白细胞、血小板两组无显著性差异($P>0.05$)。化疗急性及亚急性毒性分度:急性及亚急性毒性分度比较,两组无显著差异($P>0.05$)。

2.4 对 TSGF 水平的影响 两组治疗后 TSGF 水平均有所下降,但第 1 周期两组无统计学差异($P>0.05$),第 2 周期末综合组下降幅度显著大于化疗组($P<0.05$)(表 2)。

表 2 两组 TSGF 水平变化比较

时间	综合组(U/L)	化疗组(U/L)
1 周期初	71.63±12.02	70.73±12.45
1 周期末	66.96±11.26	67.25±11.87
2 周期初	67.14±11.98	67.83±11.71
2 周期末	60.03±11.31*	66.92±11.46

3 讨论

中药配合肿瘤化疗应用可以减轻化疗毒性,提高化疗完成率,增加疗效,这已在肿瘤的综合治疗中形成共识。但辅助方案制定要注重科学性、可重复性、可操作性并保持一定的灵活性。中医治疗的特点是将中医的治则治法应用于中医临床诊疗全过程,是联系辨证与用药之间的枢纽,应有相对固定的内容。制定规范化治疗方案抓住了这一环节就可以做到“方以法立,随证加减”,即抓住了研究的最佳切入点[1]。从中医理论分析,肺癌的发生、发展与机体的肾精亏损、癌毒久羁、外结内困等因素密切相关,出现‘毒、虚、瘀’并存的病理状态。化疗是目前中晚期肿瘤及术后治疗的主要方法。随着新的化疗药物不断问世,应用化疗治疗肺癌越来越广泛,而患者化疗后副作用常常使病者‘虚虚相加’,甚至因严重毒副作用导致死亡。故在化疗期间,应以补气养血、健脾和胃、滋补肝肾为原则以减少化疗毒性,提高化疗完成率,增加化疗疗效[1]。注重扶正固本,祛邪抑瘤,能调整患者机体的阴阳、气血、脏腑功能的平衡,可减轻其他治疗的毒副作用[2]。根据肺癌辨治特点自拟了用于辅助肺癌化疗的扶正抑瘤方,以求解毒祛瘀、扶正固本、调节机体各项机能,扶正而不助邪,消积而不伤正,攻补结合,促使阴平阳秘,提高肺癌化疗疗效。从我们临床疗效观察来看,通过该方辅助治疗,可以提高患者的卡氏评分,在一定程度上避免化疗引起的患者体重下降,以提高化疗患者的生活质量。同时对化疗引起的贫血及骨髓抑制有一定保护作用,提示扶正抑瘤方对化疗有一定的增效减毒作用。

此外,TSGF 为 20 世纪 80 年代发现的一种广谱肿瘤标志物[3],可用于肿瘤早期诊断和预后检测,具有较高的灵敏性和特异性,是肿瘤生长过程中产生的一组特殊物质,能促进肿瘤生长及周边毛细血管的大量增生,因此逐渐为临床所应用。鲁露等[4]研究表明,TSGF 可作为肺癌化疗有效的参考标志物之一。而本研究亦表明,晚期非小细胞肺癌患者化疗后,TSGF 水平有所下降。同时亦显示配合服用扶正抑瘤方的患者 TSGF 水平下降较单纯化疗组更明显。说明扶正抑瘤方对肺癌作用的可能机制之一是抑制 TSGF 产生。中医药针对恶性肿瘤特异性生长因子的疗法,可望成为进一步提高肺癌等恶性肿瘤患者的生存率、生存质量的新辅助疗法,对发展新的抗肿瘤治疗策略具有一定的临床意义。

参考文献:

[1] 林洪生. 建立中医肿瘤规范化治疗方案[J]. 癌症进展杂志,2005,11(6):524.

[2] 张庆荃. 扶正抑瘤方结合介入化疗治疗晚期乳腺癌 15 例[J]. 上海中医药杂志,2005,(9):26-27.

[3] 陈江谊,刘钧澄,陈普祺. 恶性肿瘤特异性生长因子对恶性肿瘤临床诊断价值[J]. 中国肿瘤临床,2002,29(8):583.

[4] 鲁露,李慧,吴峻,等. 吉西他滨联合顺铂治疗 40 例晚期非小细胞肺癌[J]. 肿瘤学杂志,2006,12(3):229.

编辑:肖元春

收稿日期:2007-02-16

(C)1994-2023 China Academic Journal Electronic Publishing House. All rights reserved. http://www.cnki.net
