# Supplementary material for: Benefit-risk evaluation of Fuzheng Yiliu Decoction combined with chemotherapy for treating non-small cell lung cancer using multicriteria decision analysis
Source: Front Oncol. 2025 Sep 23;15:1627904. doi: 10.3389/fonc.2025.1627904 (PMC12500661; doi:10.3389/fonc.2025.1627904)
Supplement: Supplementary file 2 [file Supplementaryfile2.zip › supplementary files/Included literature.zip!/literature/纳入文献/孔祥应.pdf]

扶正抑瘤汤治疗晚期非小细胞肺癌患者化疗相关性疲乏的临床观察△

孔祥应 黄 军 汪 琳 赵明丹 龙 秋 黄桂军（贵州省黔南州中医医院肿瘤科 都匀 558000）

摘要：目的：探讨扶正抑瘤汤治疗晚期非小细胞肺癌患者化疗相关性疲乏的临床疗效。方法：60 例晚期非小细胞肺癌患者随机分成观察组 32 例和对照组 28 例，根据病情采用 NP 方案化疗。观察组化疗第一天开始口服扶正抑瘤汤，100mL/次，3 次/d，连服 21d 为 1 个疗程，连续服用 2 个疗程；对照组不给予中药。分别于化疗前，化疗 7、21、42d 采用癌性疲乏量表进行评分，观察化疗后两组患者骨髓抑制程度及重组人粒细胞集落刺激因子的用量情况。结果：化疗 21、42d 后，观察组情感、认知、躯体疲乏评分和总分均较化疗前下降（$P<0.05$ 或 $P<0.01$）；对照组情感、认知、躯体疲乏评分和总分均较化疗前无明显变化（$P>0.05$）。观察组下降幅度高于对照组（$P<0.05$）。观察组骨髓抑制率和重组人粒细胞集落刺激因子注射液使用总剂量与对照组比较差异无统计学意义（$P>0.05$）。结论：扶正抑瘤汤能明显减轻晚期非小细胞肺癌患者化疗相关性疲乏，提高患者的生存质量。

关键词 扶正抑瘤汤 晚期非小细胞肺癌 化疗相关性疲乏 癌性疲乏量表

中图分类号：R734.2 文献标识码：B 文章编号：1672-8351（2016）06-0085-02

肺癌大约 70%~80%为非小细胞肺癌，多数患者就诊时肺癌已属晚期，化疗是临床上治疗肺癌的主要方法之一。肺癌患者化疗后容易产生疲乏，严重影响肺癌患者的生存质量。扶正抑瘤汤是我院院内制剂，具有清热利湿、补中益气、扶正固本、解毒散结的作用[1~2]。为了观察扶正抑瘤汤治疗晚期非小细胞肺癌患者化疗相关性疲乏的临床疗效，2013 年 8 月~2015 年 11 月，我们治疗 30 例，现报道如下：

1 资料与方法

1.1 一般资料：选择 2013 年 8 月~2015 年 11 月在贵州省黔南州中医医院肿瘤科住院经组织学或细胞学病理确诊为晚期非小细胞肺癌患者。符合标准晚期非小细胞肺癌诊断标准，年龄≥18 岁，文化程度小学以上，无意识障碍，无认知障碍，预计生存期≥6 个月，患者依从性好，签署知情同意书。排除病情严重无法完成量表者，2 个月内接受化疗者，患有心、肝、肾和造血系统疾病，患有精神疾病者或不愿意配合者，依从性差者。共收集 60 例，随机分成两组，观察组 32 例，男性 20 例，女性 12 例；年龄 48~75 岁，平均年龄（58.67±10.39）岁；功能状态评分 0 分 5 例，1 分 19 例，2 分 8 例；病理类型为腺癌 22 例；鳞癌 10 例；临床分期 IIIA 6 例，IIIB 9 例，Ⅳ17 例；病程 4~11 个月，平均病程（5.78±1.52）个月；既往化疗次数<3 次 22 例，3~4 次 10 例。含铂两药化疗方案：紫杉醇+顺铂/（紫杉醇）+卡铂 19 例，培美曲赛+顺铂/培美曲赛+卡铂/培美曲赛+奥沙利铂 13 例；血红蛋白含量 105.12~132.14g/L，平均血红蛋白含量（119.54±13.48）g/L；红细胞计数 3.31~4.72×10^12/L，平均红细胞计数（3.76±0.49）×10^12/L；

2.1 两组综合疗效比较：见表 1。

表 1 两组综合疗效比较

组别	n	显效	有效	无效	总有效（%）
观察组	50	27	18	5	45（90）1)
对照组	50	11	23	16	34（68）

注：1)与对照组比较，$P<0.05$。

2.2 两组肝功能疗效比较：见表 2。

表 2 两组治疗后肝功能指标改善情况比较

组别	时间	ALT（u/l）	AST（u/l）	TBIL（μmol/l）
观察组	治疗前	240±154	180±120	53.2±10.4
n=50	治疗后	46±24 1)2)	44±23 1)2)	21.6±9.0 1)2)
对照组	治疗前	248±125	178±118	52.7±10.6
n=50	治疗后	60±21 1)	74±24 1)	32.5±11.2 1)

注：1)与治疗前比较，$P<0.01$；2)与对照组比较，$P<0.05$。

2.3 两组患者肝纤维化血清学指标改善情况：见表 3。

表 3 两组治疗后肝纤维化血清学指标改善情况比较

组别	时间	LN（μg/mL）	HA（μg/mL）	IV-C（μg/mL）	PCIII（μg/mL）
观察组	治疗前	201.39±66.46	214.31±56.25	160.63±55.19	163.35±36.85
n=50	治疗后	100.19±31.25 1)2)	128.68±50.28 1)2)	100.25±32.8 1)2)	101.0±31.96 1)2)
对照组	治疗前	200.98±70.19	204.55±56.36	160.31±56.11	150.12±31.29
n=50	治疗后	120.53±32.98 1)	178.69±66.59 1)	132.56±41.99 1)	112.33±31.87 1)

注：1)与治疗前比较，$P<0.01$；2)与对照组比较，$P<0.05$。

2.4 两组治疗前后肝脏瞬时弹性成像检查肝脏硬度值比较：见表 4。

表 4 两组治疗前后肝脏瞬时弹性成像检查肝脏硬度值比较 （kPa，$\bar{x}\pm s$）

组别	n	治疗前	治疗后
观察组	50	9.7±3.4	5.3±2.4 1)2)
对照组	50	9.6±3.6	6.6±7.4 1)

注：1)与治疗前比较，$P<0.01$；2)与对照组比较，$P<0.05$。

2.5 不良反应观察：两组治疗期间均未见明显不良反应。

3 讨 论

本研究结果表明：在抗乙肝病毒、护肝治疗基础上加用安络化纤丸，即将抗乙肝病毒药恩替卡韦与抗肝纤维化中药强强联合，对于慢性乙型肝炎后早、中期肝硬化的疗效是目前最佳最优的，基本上能达到《慢性乙型肝炎防治指南》中指出的“最大限度地长期抑制乙肝病毒的复制，减轻肝细胞炎症坏死及肝纤维化，延缓和减少肝脏失代偿、肝硬化、肝细胞癌及其并发症的发生，从而改善生活质量和延长存活时间”的目的，延缓或阻止这部分“无毒”病例向肝硬化进展甚或逆转。显示出中医药在抗肝纤维化方面的优势。慢性乙型病毒性肝硬化，是因病毒而致肝纤维化的。现代医学抗病毒的效果中医药是无法比的，但其在抗纤维化方面到目前为止还没有理想的治疗方法和有效的药物研究出来；中医药在抗肝纤维化方面有显著的优势和特点，但抗病毒方面目前还没有与西药抗衡的方药出现。如果能使抗病毒与抗纤维化有机联合，中西合璧、互补其短、分取其长，既能实现临床“无毒”肝病患者客观存在的实际需求，也能体现出“慢性乙型病毒性肝硬化”君臣佐使选方配伍的治疗原则。如果有长期、大样本、多中心、随机双盲的中西优化结合治疗方案的研究，这一世界级难题可能会有所突破。

参考文献

[1]中华医学会肝病学分会，中华医学会感染病学分会.慢性乙型病毒性肝炎防治指南（2010 年版）[J].中华肝脏病杂志，2011，19（1）：13-24.

[2]吴勉华，王新月.中医内科学[M].北京：中国中医药出版社，2014：243-272.

[3]李兰娟，任红.传染病学[M].第 1 版，北京，人民卫生出版社，2013：17-43.

(C)1994-2023 China Academic Journal Electronic Publishing House. All rights reserved. http://www.cnki.net

白细胞计数 2.65~5.57×10^9/L，平均白细胞计数（3.97±0.43）×10^9/L。对照组 28 例，男性 18 例，女性 10 例；年龄 49~78 岁，平均年龄（59.37±11.78）岁；功能状态评分 0 分 4 例，1 分 17 例，2 分 7 例；病理类型为腺癌 20 例，鳞癌 8 例；临床分期 IIIA 4 例，IIIB8 例，Ⅳ16 例；病程 4~10 个月，平均病程（5.69±1.85）个月；既往化疗次数<3 次 20 例，3~4 次 8 例。含铂两药化疗方案：紫杉醇+顺铂/（紫杉醇）+卡铂 18 例，培美曲赛+顺铂/培美曲赛+卡铂/培美曲赛+奥沙利铂 10 例；血红蛋白含量 106.18~131.17g/L，平均血红蛋白含量（118.99±14.67）g/L；红细胞计数 3.35~4.89×10^{12}/L，平均红细胞计数（3.79±0.53）×10^{12}/L；白细胞计数 2.70~5.59×10^9/L，平均白细胞计数（3.92±0.68）×10^9/L。两组患者一般资料比较差异无统计学意义（$P>0.05$），具有可比性。

1.2 治疗方法：两组患者根据病情采用 NP 方案化疗。采用长春瑞宾 25mg/m^2，静脉注射 d1、d8；顺铂（NP）25mg/m^2，静脉滴注 d1、d2、d3，21d 为 1 个周期，共治疗 2 个周期。观察组化疗第一天开始口服扶正抑瘤汤（黄芪 15g、党参 20g、白术 10g、茯苓 15g、枳壳 10g、厚朴 9g、绿萼梅 10g、半枝莲 20g、虎杖 15g、生薏苡仁 20g、白花蛇舌草 30g、砂仁 10g、炒谷芽 15g 等煎取汁，由黔南州中医医院制剂科加工生产，真空包装，每包 100ml），100mL/次，3 次/d，连服 21d 为 1 个疗程，连续服用 2 个疗程；对照组不给予中药。化疗后出现Ⅱ度以上骨髓抑制根据病情使用重组人粒细胞集落刺激因子注射液（国药准字 S200100631，执行标准 YBS00302007，协和发酵麒麟株式会社，规格 0.3mL:75μg，0.6mL:150μg，1.2mL:300μg）皮下注射至血象正常。

1.3 观察指标：①癌性疲乏量表评分：分别于化疗前，化疗 7、21、42d 采用癌性疲乏量表进行评分。癌性疲乏量表[3]专门用于癌症患者疲劳症状的调查。共 3 个维度：情感疲乏、认知疲乏和躯体疲乏，每个维度 5 个条目，每一条目均采用 1~5 级评分，总分为 0~60 分。分数越高表明疲乏症状越严重。②骨髓抑制程度：参照文献评定两组化疗结束后骨髓抑制程度，骨髓无抑制为 0 度。骨髓抑制分为Ⅰ~Ⅳ度。分级越高表示化疗后骨髓抑制越严重。骨髓抑制率（%）=骨髓抑制病例数/总病例数×100%。③重组人粒细胞集落刺激因子注射液使用情况，计算化疗后使用的总剂量。

1.4 统计学方法：采用 SSPS 统计学软件 17.0 进行分析，计数资料采用 X^2 检验，计量资料采用 t 检验，$P<0.05$ 为差异有统计学意义。

2 结 果

2.1 脱落情况：观察组脱落 2 例，对照组脱落 1 例，总脱落率为 5.00%。最终统计病例数为 57 例，其中观察组 30 例，对照组 27 例。

2.2 两组癌性疲乏量表评分比较：化疗 21、42d 后，观察组情感、认知、躯体疲乏评分和总分均较化疗前下降（$P<0.05$ 或 $P<0.01$）；对照组情感、认知、躯体疲乏评分和总分均较化疗前无明显变化（$P>0.05$）。观察组下降幅度高于对照组（$P<0.05$），见表 1。

表 1 两组癌性疲乏量表评分比较（分，$\bar{x}\pm s$）

组别	n	时段	情感疲乏	认知疲乏	躯体疲乏	总分
观察组	30	化疗前	11.47±3.16	10.26±4.13	17.35±5.31	39.05±11.76
	30	化疗 7d	10.35±4.65	9.57±3.84	16.24±6.01	36.16±12.45
	30	化疗 21d	8.63±4.31☆	7.24±5.32☆	14.32±6.17☆	30.19±12.27★
	30	化疗 42d	7.02±2.81★△	6.56±2.15★△	12.13±4.31★△	25.47±11.43★▲
对照组	27	化疗前	11.89±3.23	10.98±4.56	17.68±5.42	39.97±11.65
	27	化疗 7d	11.23±2.77	10.21±3.86	16.54±5.62	37.98±11.01
	27	化疗 21d	10.65±3.71	9.83±4.24	16.37±4.28	36.85±12.61
	27	化疗 42d	10.17±2.97	9.49±3.76	16.63±4.74	36.29±12.33

注：与本组化疗前比较，☆$P<0.05$，★$P<0.01$；与对照组比较，△$P<0.05$，▲$P<0.01$。

2.3 两组骨髓抑制程度比较：化疗后，观察组骨髓抑制 21 例，骨髓抑制率为 70.00%；对照组骨髓抑制 23 例，骨髓抑制率为 85.19%。两组比较，差异无统计学意义（X^2=1.861，$P>0.05$），见表 2。

表 2 两组骨髓抑制程度比较 [例]

组别	n	0 度	Ⅰ度	Ⅱ度	Ⅲ度	Ⅳ度
观察组	30	9	9	7	3	2
对照组	27	4	7	8	5	3

2.4 两组重组人粒细胞集落刺激因子注射液使用总剂量比较：化疗后，观察组重组人粒细胞集落刺激因子注射液使用总剂量为 3375μg，对照组为 3675μg。两组比较，差异无统计学意义（X^2=0.571，$P>0.05$），见表 3。

表 3 两组重组人粒细胞集落刺激因子注射液使用总剂量比较（支）

组别	n	75μg/支	150μg/支
观察组	30	19	13
对照组	27	17	16

3 讨 论

癌性疲乏在恶性肿瘤患者中发生率 70%~100%，不仅发生在抗肿瘤治疗中，而且在抗肿瘤治疗结束后持续数月，甚至数年，严重影响患者的生活质量[4-5]。化疗相关性疲乏是由于化疗引起的疲乏，属癌性疲乏，目前以综合治疗为主，未见单一有效的治疗方法。化疗的副作用如肝功能损害、胃肠道反应、骨髓抑制等是导致化疗相关性疲乏的原因[6]。扶正抑瘤汤方中黄芪、党参、白术益气健脾，茯苓、生薏苡仁渗湿健脾，炒谷芽消食健胃，可减轻化疗的不良反应，达到益气补虚的目的。本文结果显示，观察组情感、认知、躯体疲乏评分和总分均较化疗前下降；对照组情感、认知、躯体疲乏评分和总分均较化疗前无明显变化，观察组下降幅度高于对照组，提示采用扶正抑瘤汤能减轻化疗相关性疲乏症状，晚期非小细胞肺癌患者服用扶正抑瘤汤能明显加快疲劳缓解速度，改善患者的生活质量，本文观察时间较短，疲劳缓解程度仅为近期缓解，远期的缓解程度尚未观察，今后我们将进行临床观察，以期准确评价扶正抑瘤汤减轻化疗相关性疲乏的临床疗效。

参考文献

[1]黄桂军，王华，陈朝宽，等.扶正抑瘤汤对晚期前列腺癌患者生存期和生活质量的影响[J].江苏中医药，2010，42（6）：18-20.

[2]黄军，孔祥应，汪琳，等.扶正抑瘤汤联合恩度治疗晚期非小细胞肺癌的疗效观察[J].现代中西医结合杂志 2015，24（9）：955-957.

[3]Okuyama T，Akechi T，Kugaya A，et al. Development and validation of the cancer fatigue scale：A brief，three-dimensional，self- rating scale for assessment of fatigue in cancer patients[J].J Pain Symptom Manage，2000，19（1）：5-14.

[4]王琦，李峻岭.癌因性疲乏的相关因素及发病机制[J].癌症进展，2011，9（1）：85-88.

[5]解婧，李睿，钱建新，等.恶性肿瘤患者癌因性疲乏的质性研究[J].中国肿瘤，2010，19（3）：171-174.

[6]章璐，曹勇.癌因性疲乏的中医辨证论治[J].四川中医，2009，27（3）：41-42.

△基金项目：贵州省中医药管理局项目（GZYY2011078）

(C)1994-2023 China Academic Journal Electronic Publishing House. All rights reserved. http://www.cnki.net
