# Supplementary material for: Benefit-risk evaluation of Fuzheng Yiliu Decoction combined with chemotherapy for treating non-small cell lung cancer using multicriteria decision analysis
Source: Front Oncol. 2025 Sep 23;15:1627904. doi: 10.3389/fonc.2025.1627904 (PMC12500661; doi:10.3389/fonc.2025.1627904)
Supplement: Supplementary file 2 [file Supplementaryfile2.zip › supplementary files/Included literature.zip!/literature/纳入文献/李日良.pdf]

扶正抑瘤汤治疗气虚血瘀型非小细胞肺癌化疗患者的临床观察

李日良

烟台有色金属诊所　山东　烟台　264002

[摘要] 目的：观察气虚血瘀型非小细胞肺癌化疗患者经扶正抑瘤汤治疗的临床疗效。方法：抽选 2019 年 4 月至 2020 年 4 月间 48 例非小细胞癌患者，随机分组，所有患者均采取 NP 方案化疗，观察组患者给予扶正抑瘤汤治疗，对照组未给予中药。结果：观察组疗效 95.83% 优于对照组 75.00%，P ＜ 0.05；观察组行为状态评分 KPS 优于对照组，P ＜ 0.05。结论：非小细胞肺癌化疗患者经中药扶正抑瘤汤治疗，疗效显著，可有效改善行为状态评分，提升患者生存质量。

[关键词] 非小细胞肺癌；气虚血瘀型；扶正抑瘤汤；临床疗效

非小细胞肺癌主要包括腺癌与鳞癌，约为整体肺癌患者 70%，此类疾病发病较为隐匿，且具有一定复发性，预后不佳，发病后 5 年生存率低于 15%，若未及时诊治，生存期在 7–15 月间。目前对于非小细胞肺癌，临床常选取化疗、放疗、手术治疗等方式干预，可延缓病情发展，其中 NP 化疗方案应用较广，可有效延长患者生存期，但化疗后可能发生毒复反应，因此需辅以有效药物减缓副作用 [1]。中医理论中，认为癌灶多与正气亏虚、内侵瘀毒有关，结合患者病症特征可归于气虚血瘀证，常选用扶正抑瘤汤治疗。本文围绕 48 例非小细胞肺癌患者开展研究，报告如下。

1 资料和方法

1.1 资料

于 2019 年 4 月至 2020 年 4 月间非小细胞肺癌患者中抽选 48 例，随机分组。观察组，13 例男性、11 例女性，年龄 38–74 岁，均值（51.73 ± 4.38）岁；对照组，12 例男性、12 例女性，年龄 39–75 岁，均值（51.82 ± 4.43）岁。48 例患者中，36 例鳞癌、11 例腺癌、1 例透明细胞癌。伦理委员会批准。非小细胞癌患者自愿参与，分析患者基线数据，P ＞ 0.05。

1.2 治疗方法

化疗方法：研究样本选取 NP 方案治疗，静脉注射长春瑞宾，剂量为 25mg/m²，静脉滴注顺铂，剂量为 25mg/m²。1 周期为 21d，持续给药 2 周期。

对照组未给予中药，观察组给予扶正抑瘤汤治疗，药方如下：白花蛇舌草 30g，党参、半枝莲、生薏苡仁各 20g，黄芪、茯苓、炒谷芽、虎杖各 15g，白术、绿萼梅、枳壳、砂仁各 10g，厚朴 9g。上述各味中药以水煎至 100ml，每日服用 3 次，1 疗程为 21d，持续给药 2 疗程。对于化疗后骨髓抑制超过Ⅱ度患者，结合实际情况给予重组人粒细胞集落刺激因子注射液，给药方式为皮下注射。

1.3 观察指标

对比组间疗效，依据《中药新药临床研究指导原则》，将疗效分为显效、有效及无效三档 [2]；对比组间 KPS 评分变化，满分 100 分，分数高提示患者行为状态恢复良好。

1.4 统计学研究

非小细胞肺癌化疗期间各项数据以 SPSS 33.0 处理，选取 % 形式代表计数资料，选取 $\bar{x}$ ± s形式代表计量资料，X^2 或 t 验证统计差异。P ＜ 0.05，存在对比意义。

2 结果

2.1 非小细胞肺癌疗效分析

扶正抑瘤汤干预后，观察组疗效达 95.83%，相较于对照组 75.00%，P ＜ 0.05。如表 1。

表 1 非小细胞肺癌患者疗效分析表（n,%）

组别名称	显效	有效	无效	有效率
观察组（n=24）	16（66.67）	7（29.17）	1（4.17）	95.83
对照组（n=24）	14（58.33）	4（16.67）	6（25.00）	75.00
X^2	–	–	–	4.1812
P	–	–	–	＜ 0.05

2.2 非小细胞肺癌 KPS 评分分析

选取扶正抑瘤汤治疗后，观察组 KPS 评分（84.67 ± 5.11）分显著优于对照组（76.24 ± 4.68）分，P ＜ 0.05；药物干预前，分析组间 KPS 评分，P ＞ 0.05。如表 2。

表 2 非小细胞肺癌患者 KPS 评分分析表（$\bar{x}$ ± s）

组别名称	给药前	给药后
观察组（n=24）	72.43 ± 4.25	84.67 ± 5.11
对照组（n=24）	72.46 ± 4.27	76.24 ± 4.68
t	0.0244	5.9600
P	＞ 0.05	＜ 0.05

3 讨论

结合中医理论分析，非小细胞肺癌可归于"肺积"、"虚劳"范畴，其发病机制主要与机体正气亏虚、邪气入侵有关，此外，患者情志不遂、饮食不洁导致气机阻滞也可诱发本病。机体内可行气则可行血，气行受阻则血运阻滞，久滞可成瘀，若未及时诊治则形成肿块引发肺癌，因此血瘀为肺癌患者根本病机。中医辩证非小细胞肺癌，症型繁多，且尚未统一，本次研究旨在探析气虚血瘀型患者，结合患者病症表现给予益气、活血、化瘀治疗，在西医化疗基础上给予扶正抑瘤汤治疗 [3]。扶正抑瘤汤方中苦杏仁、桔梗、山慈菇与浙贝母等药物，能够宣发肺气、润肺化痰，进而缓解肺癌患者咳嗽、咳痰等病症；方中太子参、黄芪、茯苓、炒白术与当归等药物，能够补血、益气，可缓解肺癌患者乏力、气短等病症；方中香附、鸡血藤与地龙等药物，能够化瘀活血，可缓解胸痛不适等病症；方中鸡内金与焦山楂能够调节脾胃，可缓解便秘，提升患者食欲，改善消化系统病症 [4]。结合本次研究，观察组疗效达 95.83% 优于 75.00%，且 KPS 评分优于对照组，提示扶正抑瘤汤能够调节阴阳、平衡脏腑，进而益气活血，缓解肺癌患者不适，改善患者生存质量。此外，扶正抑瘤汤还可预防骨髓抑制，缓解恶心、呕吐等不良反应，且本次研究内未见肝肾功能不良影响，提示扶正抑瘤汤安全可行。

综上所述，对于气虚血瘀型非小细胞肺癌患者，化疗基础上给予扶正抑瘤汤治疗，中西医结合，可有效降低化疗毒副反应，缓解肺癌引发临床症状，进而改善患者生存质量，延长生存期。因此，扶正抑瘤汤可作为化疗非小细胞肺癌患者首选干预药物。

参考文献：

[1] 李燕，刘怀民，蒋立峰，et al. 血府逐瘀汤加减对高凝状态气虚血瘀型非小细胞肺癌患者生存质量的影响 [J]. 社区医学杂志，2018, 16(010):47−48.

[2] 叶知锋，黄挺，杨雪飞，等．晚期非小细胞肺癌肿瘤相关抑郁状态中医证型分布研究 [J]. 江苏中医药，2018, 50(001):35−38.

[3] 张一乐，陈麒，张炜．扶正抗岩方联合吉非替尼治疗气虚血瘀型非小细胞肺癌（Ⅳ期）的前瞻性、平行随机、安慰剂对照临床研究 [J]. 上海中医药杂志，2019, 53(03):37−41.

[4] 杨杨．探讨晚期非小细胞肺癌患者气虚血瘀证的研究进展 [J]. 医药前沿，2018, 008(023):14−15.

"健身气功·八段锦"对大学生部分生理指标以及体质的影响分析

李文娟　李圆圆　宫晓静　徐彬彬　杜正阳

青岛黄海学院　山东　青岛　266427

[摘要] 目的：分析大学生经过"健身气功·八段锦"练习后的生理指标及体质指标的变化。方法：以青岛某民办高校的 60 名不爱运动的大学生作为研究对象，所有大学生进行为期 3 个月的八段锦运动练习，以成分指标、身体机能指标、身体素质指标为观察指标，通过观察运动前、运动后的各指标的变化，分析"健身气功·八段锦"对大学生身体素质的影响。结果：经过八段锦运动练习后大学生所测体成分指标均较运动前的各项指标值有所下降，且体脂率、脂肪、体重与运动前有显著性差异，P<0.05；大学生在八段锦运动后的心率较运动前要低，肺活量较运动前高，且运动前和运动后有显著性差异，P<0.05；经八段锦运动后大学生的立定跳远和仰卧起坐项目的能力较运动前均有所改善，P<0.05，运动前后有显著性差异，差异均具有统计学意义。结论："健身气功·八段锦"能有效改善大学生的体成分指标、身体机能指标、身体素质指标，有利于大学生身体健康。

[关键词] 健身气功·八段锦；大学生；身体素质；生理指标

健身气功·八段锦作为我国传统的养生项目之一，具有悠久的历史，其在中医理论的基础上不断发展，并形成了南北两个派系，八段锦简单易学、舒展大方的招式不仅能促进身体的平衡和肢体协调，还可以改善我们的生活心态，促进身体的新陈代谢，达到养生的目的 [1–2]。大学生是我国未来的希望，随着文化素养的不断提升，但身体素质却堪忧。本文将以大学生作为研究对象，观察八段锦对大学生的生理指标和体质的影响。

1 资料与方法

1.1 一般资料

于青岛某民办高校选取 60 名不爱运动的大学生作为研究对象，年龄段分布为 18~22 岁，平均年龄处于 20.1 ± 0.76 岁，男生占比 45%，女生占比 55%。本次研究对象均自愿参加，并签署同意书。

1.2 方法

所有大学生进行为期 3 个月的八段锦运动练习，主要为以下内容：由专门的八段锦培训老师向大学生讲解整套动作的技术要领，共包括预备、一式、二式、三式、四式、五式、六式、七式、八式、结束动作，直至所有大学生全部学会所有动作。每周练习 5 次八段锦的整套动作，每次练习时长达 40 分钟，其中包括热身运动 5 分钟。在整个运动练习的时间里，控制心率为 110–130 次 / 分。

1.3 观察指标

观察指标主要为以下：①体成分指标：BMI 指数、体脂率、脂肪含量、蛋白质含量、体重；②身体机能指标：心率、肺活量；③身体素质指标：立定跳远、仰卧起坐、坐位体前屈。

1.4 统计学方法

采用 SPSS20.0 软件对所有大学生的各项指标值进行统计学处理，各项资料属于计量资料，采用 t 检验，表示方式为均数 ± 标准差，若 P ＜ 0.05，代表具备统计学意义。

2 结果

2.1 大学生体成分指标比较

由表 1 可知，经过八段锦运动练习后大学生所测体成分指标均较运动前的各项指标值有所下降，且体脂率、脂肪、体重与运动前有显著性差异，P<0.05，差异均具有统计学意义。

表 1 大学生体成分指标比较（$\bar{x}$ ± s）

组别	例数	BMI 指数	体脂率	脂肪 /kg	蛋白质 /kg	体重 /kg
运动前	60	22.31 ± 2.56	26.73 ± 4.13	15.98 ± 4.01	8.54 ± 0.98	59.45 ± 2.31
运动后	60	22.14 ± 2.31	25.22 ± 3.62	14.63 ± 3.35	8.23 ± 0.94	57.66 ± 1.67
t		0.3819	2.1297	2.0013	1.7683	4.8643
P		0.7032	0.0353	0.0477	0.0796	0.0000

2.2 大学生身体机能指标比较

由表 2 可知，大学生在八段锦运动后的心率较运动前要低，肺活量较运动前高，且运动前和运动后有显著性差异，P<0.05，差异均具有统计学意义。

表 2 大学生身体机能指标比较（$\bar{x}$ ± s）

组别	例数	心率 (c/min)	肺活量 ml
运动前	60	70.44 ± 9.78	2456.60 ± 500.30
运动后	60	66.95 ± 8.43	2652.60 ± 433.50
t		2.0937	2.2934
P		0.0384	0.0236

2.3 大学生身体素质指标比较

由表 3 可知，经八段锦运动后大学生的立定跳远和仰卧起坐项目的能力较运动前均有所改善，P<0.05，运动前后有显著性差异，差异具有统计学意义。

表 3 大学生身体素质指标比较（$\bar{x}$ ± s）

组别	例数	立定跳远 cm	仰卧起坐 c/min
运动前	60	155.40 ± 18.70	29.40 ± 6.89
运动后	60	168.50 ± 15.60	34.20 ± 8.42
t		4.1668	3.4174
P		0.0001	0.0009

3 讨论

"健身气功·八段锦"属于运动负荷小、低强度的有氧耐力运动，男女老少均适合，已有研究表明，八段锦能降低安静心率，改善大学生身体素质 [3]。不仅如此，连许多中老年人都热爱这项运动，目前该运动项目在学校还未得到推广和普及。本研究以大学生为研究对象，体成分指标、身体机能指标、身体素质指标为观察指标，结果均证实了"健身气功·八段锦"对于当代大学生来说，是一种良好的锻炼与健身方式，是一种有利于大学生身体健康、提高身体素质的运动项目。本项目研究周期仅为三个月，后期进一步研究将延长研究周期和增加量化指标。

综上所述，"健身气功·八段锦"能有效改善大学生的体成分指标、身体机能指标、身体素质指标，有利于大学生身体健康，值得各学校大力推广。

参考文献

[1] 臧宝斌．健身气功·八段锦的发展特征分析 [J]. 拳击与格斗，2019,34(14):137.

[2] 何丽芊，张旭，宝林．健身气功·八段锦对大学生体质健康的影响研究 [J]. 文体用品与科技，2017,40(24):47−48.

[3] 王凤杰．健身气功·八段锦练习对大学女生身体意象的影响 [J]. 武术研究，2019,4(4):95−97.

课题来源：院级课题"八段锦对民办高校大学生身心健康效应的实验研究"

作者简介：李文娟（1988−），女，硕士，青岛黄海学院，助教，主要研究方向为中医、康复。
