# Supplementary material for: Benefit-risk evaluation of Fuzheng Yiliu Decoction combined with chemotherapy for treating non-small cell lung cancer using multicriteria decision analysis
Source: Front Oncol. 2025 Sep 23;15:1627904. doi: 10.3389/fonc.2025.1627904 (PMC12500661; doi:10.3389/fonc.2025.1627904)
Supplement: Supplementary file 2 [file Supplementaryfile2.zip › supplementary files/tables.zip!/tables/Table 1 Search strategy_20250904120901.pdf]

Table 1 Search strategy

Data base	Search strategy
PubMed	(FuzhengYiliutang[Title/Abstract])
Web of Science	TS=(FuzhengYiliutang) AND (NSCLC OR non-small cell lung cancer) AND (Cisplatin-resistant)
CNKI	(FuzhengYiliutang) AND (NSCLC OR non-small cell lung cancer) AND (Cisplatin-resistant)
Wan Fang	(FuzhengYiliutang) AND (NSCLC OR non-small cell lung cancer) AND (Cisplatin-resistant)
VIP	(FuzhengYiliutang) AND (NSCLC OR non-small cell lung cancer) AND (Cisplatin-resistant)

Table 2 Benefit-risk indicator weights, optimal values, and worst values for multicriteria decision analysis(MCDA)

Classification	Weight %	Indicator	Weight %	Optimal Value	Worst Value
Benefit	75	KPS score	80	11	2
		CA211	60	6	3
		CEA	60	12	6
		Cancer Fatigue Scale score	100	18	0
Risk	25	gastrointestinal adverse reactions	100	0	1

Table 3 Basic information of included literature. ①KPS sores ②CA211 ③ CEA ④ Cancer Fatigue Scale score ⑤ gastrointestinal adverse reactions

Literature	E\C	Control	Experimental	Outcome
ZhangWei[29]	45/45	chemotherapy	combined chemotherapy with Fuzheng Yiliu Decoction	④
Sun Ailin[30]	29/28	chemotherapy	combined chemotherapy with Fuzheng Yiliu Decoction	①②③⑤
Lv Pengqiang[31]	30/30	chemotherapy	combined chemotherapy with Fuzheng Yiliu Decoction	①②③⑤
Zhang Wei- wei[32]	20/20	chemotherapy	combined chemotherapy with Fuzheng Yiliu Decoction	①
Kong Xiangying[33]	30/30	chemotherapy	combined chemotherapy with Fuzheng Yiliu Decoction	④
Li Riliang[34]	24/24	chemotherapy	combined chemotherapy with Fuzheng Yiliu Decoction	①

Table 4 Merger results of Experimental and control of each indicator

Primary indicator	Secondary indicator	Experimental		Control	
		RCT	Combined results(95%CI,P<0.0001)	RCT	Combined results (95%CI,P<0.0001)
Benefit	KPS score	4	-9.38 （-10.91, -7.86）	4	-3.63 (-5.35, -1.92)
	CA211	2	4.98 (4.24, 5.72)	2	3.89 (3.19, 4.60)
	CEA	2	9.96 (8.68, 11.25)	2	7.58 (6.50, 8.67）
	Cancer Fatigue Scale score	2	14.58 [11.17, 17.98]	2	3.70 (0.29, 7.11)
Risk	gastrointestinal adverse reactions	2	0.44[0.31,0.57]P=0.36	2	0.76[0.62, 0.86]P<0.001

Table 5 Weight and weight score of each indicator for alone and combined

Classification	Weight%	Indicator	Weight%	Benefit value / risk value		Relative weight %
				Experimental	control	
Benifit	75	KPS score	80	82	18	20
		CA211	60	67	33	15
		CEA	60	75	25	15
		Cancer Fatigue Scale score	100	66	36	25
Overall benefit value				72	29	
Risk	25	gastrointestinal adverse reactions	100	56	24	25
Overall risk value				56	24	
Overall benefit-risk value				68	27	
