# Supplementary material for: Benefit-risk evaluation of Fuzheng Yiliu Decoction combined with chemotherapy for treating non-small cell lung cancer using multicriteria decision analysis
Source: Front Oncol. 2025 Sep 23;15:1627904. doi: 10.3389/fonc.2025.1627904 (PMC12500661; doi:10.3389/fonc.2025.1627904)
Supplement: Supplementary file 2 [file Supplementaryfile2.zip › supplementary files/cover letter.docx]

Dear Editors,

We are pleased to submit our manuscript entitled "**Benefit-risk evaluation of Fuzheng Yiliu Decoction combined with chemotherapy for treating non-small cell lung cancer using multicriteria decision analysis (MCDA)**" for consideration for publication in **STATISTICS IN MEDICINE**. We confirm that this manuscript has not been published elsewhere and is not under consideration by another journal.

In this study, we utilized multi-criteria decision analysis (MCDA) to evaluate the benefits and risks of combining traditional Chinese medicine, specifically Fuzheng Yiliu Decoction, with chemotherapy for the treatment of non-small cell lung cancer (NSCLC). Our objective was to integrate traditional Chinese medicine with conventional chemotherapy to improve clinical outcomes for NSCLC patients. Through a comprehensive literature review, we identified five randomized controlled trials (RCTs) comparing chemotherapy alone with chemotherapy combined with Fuzheng Yiliu Decoction.

Our findings revealed that combination therapy resulted in significantly higher benefit values compared to chemotherapy alone, indicating enhanced therapeutic efficacy and reduced adverse effects of chemotherapy. The MCDA model showed a notable benefit-risk value for combination therapy, suggesting it as a promising treatment strategy for NSCLC. This study provides valuable insights for improving treatment approaches and clinical decision-making in the management of NSCLC.

All authors have approved the manuscript and agree with its submission to STATISTICS IN MEDICINE. We have no conflicts of interest to disclose. We believe that our manuscript would be of interest to the journal's readers due to its innovative approach in evaluating treatment strategies for NSCLC and its relevance to the field of statistics in medicine.

Thank you for considering our manuscript for publication in STATISTICS IN MEDICINE.

Sincerely,

*Corresponding Author:*

Dr. Jianjun Wu

Email: wjj@gszy.edu.cn
